# Precursor peptide-targeted mining of more than one hundred thousand genomes expands the lanthipeptide natural product family

**DOI:** 10.1186/s12864-020-06785-7

**Published:** 2020-06-03

**Authors:** Mark C. Walker, Sara M. Eslami, Kenton J. Hetrick, Sarah E. Ackenhusen, Douglas A. Mitchell, Wilfred A. van der Donk

**Affiliations:** 1 Department of Chemistry and Chemical Biology, University of New Mexico, 346 Clark Hall, 300 Terrace St. NE, Albuquerque, NM 87131 USA; 2 Department of Chemistry, University of Illinois at Urbana-Champaign, Roger Adams Laboratory, 600 S. Mathews Ave, Urbana, IL 61801 USA; 3 Carl R. Woese Institute for Genomic Biology, University of Illinois, Urbana, IL 61801 USA; 4 Department of Microbiology, University of Illinois at Urbana-Champaign, 601 S. Goodwin Ave, Urbana, IL 61801 USA; 5 Howard Hughes Medical Institute, University of Illinois at Urbana-Champaign, 600 S. Mathews Ave, Urbana, IL 61801 USA

**Keywords:** Lanthipeptide, RiPP, RODEO, Natural product, Genome-mining, Antibiotic

## Abstract

**Background:**

Lanthipeptides belong to the ribosomally synthesized and post-translationally modified peptide group of natural products and have a variety of biological activities ranging from antibiotics to antinociceptives. These peptides are cyclized through thioether crosslinks and can bear other secondary post-translational modifications. While lanthipeptide biosynthetic gene clusters can be identified by the presence of genes encoding characteristic enzymes involved in the post-translational modification process, locating the precursor peptides encoded within these clusters is challenging due to their short length and high sequence variability, which limits the high-throughput exploration of lanthipeptide biosynthesis. To address this challenge, we enhanced the predictive capabilities of Rapid ORF Description & Evaluation Online (RODEO) to identify members of all four known classes of lanthipeptides.

**Results:**

Using RODEO, we mined over 100,000 bacterial and archaeal genomes in the RefSeq database. We identified nearly 8500 lanthipeptide precursor peptides. These precursor peptides were identified in a broad range of bacterial phyla as well as the Euryarchaeota phylum of archaea. Bacteroidetes were found to encode a large number of these biosynthetic gene clusters, despite making up a relatively small portion of the genomes in this dataset. A number of these precursor peptides are similar to those of previously characterized lanthipeptides, but even more were not, including potential antibiotics. One such new antimicrobial lanthipeptide was purified and characterized. Additionally, examination of the biosynthetic gene clusters revealed that enzymes installing secondary post-translational modifications are more widespread than initially thought.

**Conclusion:**

Lanthipeptide biosynthetic gene clusters are more widely distributed and the precursor peptides encoded within these clusters are more diverse than previously appreciated, demonstrating that the lanthipeptide sequence-function space remains largely underexplored.

## Background

**Fig. 1:**
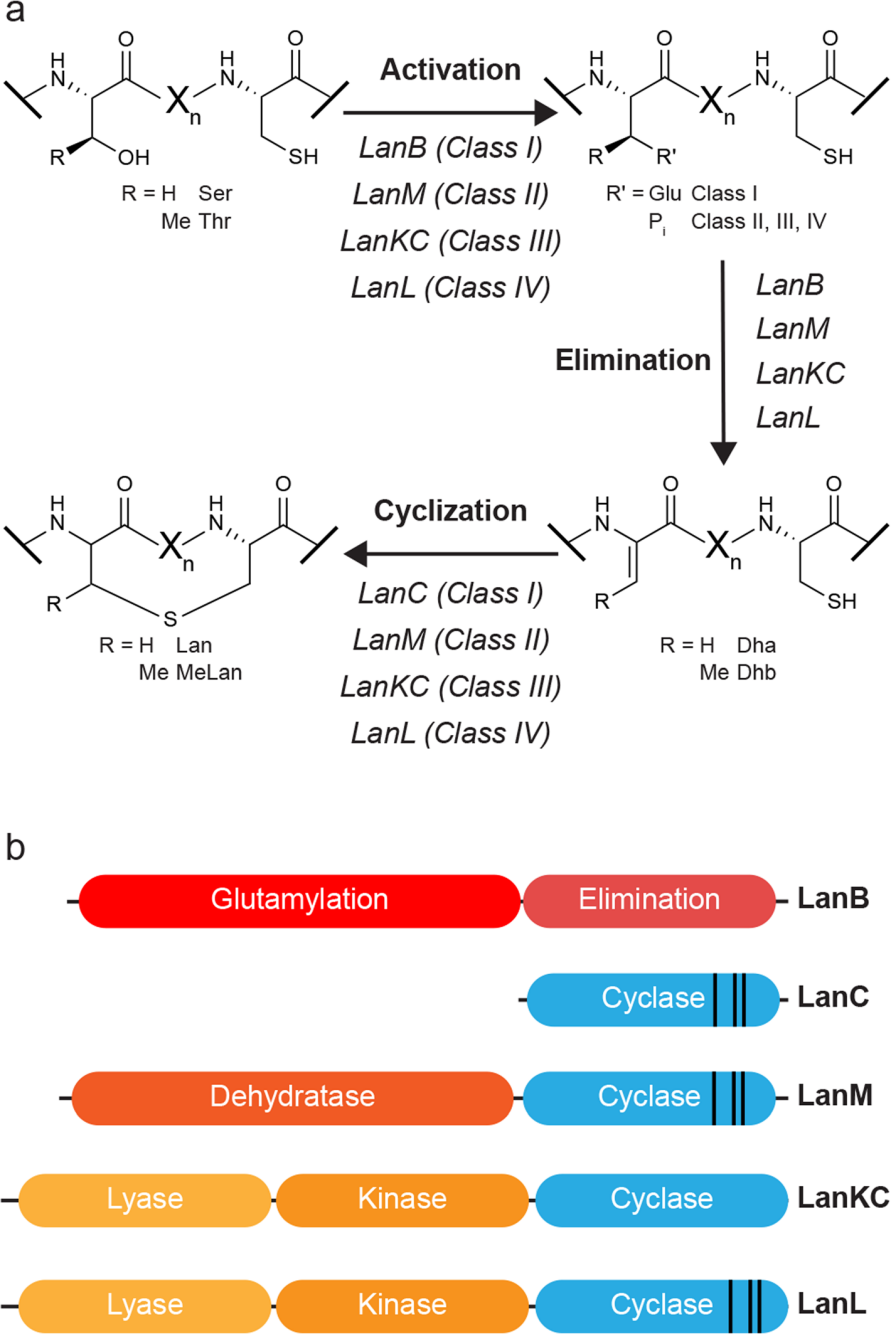
Biosynthesis of lanthipeptides. **a** Installation of lanthionine or methyllanthionine thioether crosslinks in the four different classes of lanthipeptides. Dha: dehydroalanine, Dhb: dehydrobutyrine, Lan: lanthionine, MeLan: methyllanthionine. **b** Domain structure of the enzymes that install the thioether crosslinks in the different classes of lanthipeptides. The cyclase domains shared between the classes belong to the Pfam family PF05147. The black lines in the cyclase domains represent the location of the zinc-binding residues

Ribosomally synthesized and post-translationally modified peptides (RiPPs) are an expanding group of natural products [1]. Lanthipeptides are among the most studied RiPPs and have a diverse array of structures and biological activities, including antibiotic [2–5], anti-fungal [6], anti-HIV [7, 8], and antinociceptive [9, 10] activities. Recent studies have demonstrated important roles of lanthipeptides produced by the human microbiome in disease and disease prevention [11, 12]. These peptidic natural products are characterized by the presence of macrocycles formed via thioether crosslinks between amino acid residue side chains, termed lanthionines or methyllanthionines [13]. Lanthipeptides are synthesized from a genetically encoded precursor peptide, generically named LanA, which can be divided into two portions; an N-terminal leader region, involved in recognition by the biosynthetic machinery, and a C-terminal core region, which is post-translationally modified. The essential enzymes in lanthipeptide biosynthesis dehydrate select serine and threonine residues in the core region to form dehydroalanine (Dha) and dehydrobutyrine (Dhb) residues, respectively, and then catalyze the conjugate addition of cysteine thiols onto the resulting alkenes to form the lanthionine or methyllanthionine crosslinks (Fig. 1a). Lanthipeptides can be divided into four classes based on the essential biosynthetic enzymes [13]. In class I lanthipeptides, separate proteins carry out the dehydration (LanB) [14] and cyclization (LanC) [15] reactions. LanB enzymes activate serine and threonine residues by glutamylation in a tRNA-dependent manner and produce the dehydrated residues through beta-elimination of glutamate. In classes II-IV, a single protein carries out dehydration and cyclization (LanM, LanKC, and LanL, respectively) (Fig. 1b) [13]. The C-terminal cyclization domains of LanMs, LanKCs, and LanLs are homologous to LanC cyclases; however, the LanKC cyclization domain lacks the zinc-binding residues that are conserved in the other cyclases [16]. The LanM dehydratase domain is related to lipid kinases and has acquired phosphate elimination activity in the kinase active site [17] whereas the LanKC and LanL proteins catalyze dehydration using dedicated kinase [18] and lyase domains. LanL proteins are related to OspF, a phosphothreonine lyase from certain pathogenic proteobacteria (InterPro entry IPR003519) [19]. Beyond dehydratases and cyclases, lanthipeptide biosynthetic gene clusters (BGCs) often encode transporters and proteases to remove the leader peptide (LanT/LanP) and sometimes additional enzymes that further decorate lanthipeptides with secondary modifications [13, 20].

Genome-mining studies based on these enzymes have revealed that lanthipeptide BGCs are distributed widely across bacterial phyla [21–32]. Despite the success in bioinformatically identifying likely lanthipeptide BGCs, it has been an outstanding challenge to perform high-throughput analysis of the precursor peptides encoded in these gene clusters. Because of the short length of the genes encoding LanAs, they are often not annotated as genes and their variability renders identifying new precursors through homology searching challenging. To address this problem, we have expanded Rapid ORF Description & Evaluation Online (RODEO) [33] to predict lanthipeptide precursor peptides and mined the bacterial and archaeal genomes in the RefSeq database for new lanthipeptide natural products.

## Results

### Identification of potential lanthipeptide biosynthetic gene clusters

Potential lanthipeptide BGCs were identified by searching the non-redundant RefSeq database (release 93) with the LANC_like (PF05147) hidden Markov model (HMM) from the Protein family (Pfam) database [34], as this domain is shared among currently known classes of lanthipeptides (Fig. 1b). This search resulted in 12,705 proteins with LanC-like domains. The genomic context of these proteins was then examined to assign the clusters to the four separate lanthipeptide classes. If any of the proteins encoded in the seven genes upstream or downstream from the LanC-domain containing protein matched the Pfam HMMs for a LanB (PF04738 and PF14028), the cluster was categorized as class I. If the encoded proteins matched the Pfam HMM for the dehydratase domain of a LanM (PF13575), the cluster was categorized as class II. If the protein containing the LanC-like domain also matched the Pfam HMM for a protein kinase (PF00069), the cluster was categorized as class III or class IV. Classes III and IV were then separated using custom HMMs to distinguish LanKCs (class III) from LanLs (class IV). If none of the encoded proteins matched with these Pfam HMMs, the cluster was categorized as unclassified. This sorting resulted in 2753 putative class I lanthipeptide BGCs, 3708 class II BGCs, 2377 class III BGCs, 815 class IV BGCs, and 3052 unclassified sequences. With the exception of 33 putative class II BGCs from Archaea, lanthipeptide BGCs were exclusively identified in bacteria. Of the unclassified proteins, 1279 are likely not within lanthipeptide BGCs as these proteins are more similar to other proteins, such as endogluconases [15]. In another 381 cases of unclassified proteins, the gene encoding the protein is within 3 kb of the beginning or end of a sequencing contig, suggesting incomplete data on the BGC. Intriguingly, a number of the remaining 1392 unclassified proteins are located within BGCs that encode proteins often associated with RiPP biosynthesis, such as ABC transporters and proteases, suggesting these clusters are potentially involved in the biosynthesis of an as-of-yet uncharacterized class of RiPP.

### Identification of precursor peptides

Having identified potential lanthipeptide BGCs, we set out to identify the cognate precursor peptide(s). The DNA sequence encompassing seven genes downstream to seven genes upstream of the LanC-like domain-containing protein was searched for potential open reading frames (ORFs) beginning with an ATG, TTG, or GTG start codon. Potential ORFs that encoded peptides within the expected length range for LanAs (30–120 amino acids) and not located entirely within an annotated ORF were identified for scoring. A random subset consisting of 20% of the BGCs for each class were manually examined and the identified peptides were annotated as a precursor peptide or not based on characteristics such as similarity to lanthipeptide precursor Pfam families, being encoded immediately upstream or downstream and on the same strand as the class-defining modification enzyme, and the prevalence of Ser, Thr, and Cys residues at the C-terminus. If a precursor peptide could not be unambiguously identified in the BGC, all of the potential peptides from that cluster were set aside. Next, 2458 features were calculated for the peptides deemed to be lanthipeptide precursors (Supplementary Figure S1, Additional File 1) and ANOVA was used to identify the features that were most significantly different (*p*-value < 0.05) between high-confidence precursor peptides and likely peptides arising from translation of noncoding regions. These features were then calculated for the entire set of potential precursor peptides for each class, and the manually annotated peptides were used as a training set for support vector machine (SVM) classification of the peptides as precursor or not. The SVM classification, the presence of sequence motifs in the leader peptide, and other features were used in the RODEO framework to identify potential precursor peptides for the entire RefSeq database (Supplementary Tables S1, S2, S3, S4, Additional File 1). These improvements have been incorporated into the web tool and command line versions of RODEO and are publicly accessible (http://ripp.rodeo).

This approach resulted in the identification of 8405 precursor peptides (Additional File 2). Of these putative LanAs, 2698 (32% of the total) were from class I BGCs, 3002 (36%) were from class II BGCs, 2304 (27%) were from class III BGCs, and 401 (5%) were from class IV BGCs. Based on the number of times their cognate modifying enzymes are encoded in the genome data set, these precursors represent approximately 30,000 redundant lanthipeptides. Approximately 24% of class I precursors, 17% of class II precursors, 55% of class III precursors, and 86% of class IV precursors were not annotated as genes in the database. The majority of precursor peptides in class I (62%), class III (57%), and class IV (83%) BGCs are the only predicted precursor peptide in the cluster. Precursors in class II BGCs are roughly equally split between BGCs with a single precursor peptide (37%) and those with two precursor peptides (39%). Notable exceptions to this distribution are a class I BGC from *Tumebacillus flagellates* that encodes 10 distinct precursor peptides, a class II BGC from *Herbidospora mongoliensis* with 6 distinct precursor peptides, and a class III cluster from *Bacillus cereus* with 13 identical precursor peptides. The most abundant, ungapped sequence motifs from the leader and core regions of each class were identified using Multiple Em for Motif Elicitation (MEME) (Supplementary Figure S2, Additional File 1) [35]. None of the leader peptide motifs were shared among the four lanthipeptide classes, which was expected given the differences in the respective lanthionine biosynthetic proteins. Interestingly, the most abundant core peptide motifs from each class were also restricted to that class. For example, the nisin/gallidermin lipid II-binding motif SxxxCTP(G/S) C [36] is only found in class I precursors and the mersacidin lipid II-binding motif TxTxEC [37, 38] is only found in class II precursors. Examining these sequence motifs also reveals that in addition to the long-recognized FxLD sequence motif in the leader peptides of class I LanAs [39], a number of class I LanAs from Bacteroidetes have an LxLxKx_5_L motif instead. Many of the leader peptides that contain this motif end with a Gly-Gly sequence, and a C39-family Cys protease that removes leader peptides at GG sites [40, 41] is often encoded in the corresponding clusters. This GG leader motif has previously only been observed in class II [42] and III LanAs [43]. With the identification of these class I LanAs, approximately one third of all LanAs have a GG motif at the end of the leader peptide. Double-Gly motif leader peptides are also a common occurrence in other RiPP classes [1, 44]. Other frequently observed leader peptide sequence motifs are the (E/D − 8)(L/M − 7) motif in class II [45] and the LxLQ motif in class III lanthipeptide precursors (Supplementary Figure S2) [46]. Additional less frequent motifs that have not been experimentally investigated are depicted in Supplementary Figure S2.

### Comparison with other genome mining tools

To explore the effectiveness of RODEO to predict precursor peptides, these results were compared to other genome mining packages. To achieve this comparison, 5240 genome records encoding identified lanthipeptide BGCs were submitted to antiSMASH 5.0 [47, 48]. AntiSMASH identified a similar number of BGCs as the above analysis, which is to be expected as both approaches utilize Pfam HMMs to identify the clusters, although antiSMASH does not distinguish between class III and class IV BGCs. AntiSMASH identified 55% of class I, 70% of class II, and 47% of class III or IV precursor peptides that were identified by RODEO. On the other hand, RODEO identified 93% of class I, 38% of class II, and 93% of class III or IV precursor peptides that were identified by antiSMASH. The majority of class II precursor peptides predicted by antiSMASH and not by RODEO appear to be false positives as 68% of those peptides are encoded in BGCs with at least one other precursor peptide identified by both tools. These putative false positive peptides have leader peptides that share neither similarity with the peptide identified by both tools nor with each other (when three or more putative precursor peptides were predicted in a BGC). This lack of leader peptide homology calls into question whether these peptides would be modified by the same set of enzymes as most examples of experimentally verified BGCs that contain multiple precursor peptides show high sequence identity in their leader peptides [41, 49, 50]. Next, ten randomly selected genome records encoding each class of lanthipeptide BGC were selected and submitted to the web server BAGEL4 [29]. BAGEL4 identified 70, 90, 60, and 70% of class I-IV BGCs, respectively, and identified the precursor peptides as open reading frames, but typically did not predict which open reading frame in the BGC was the precursor peptide. Thus, the improvements in RODEO for lanthipeptide precursor peptide annotation described here provide both more information and higher confidence predictions.

**Fig. 2:**
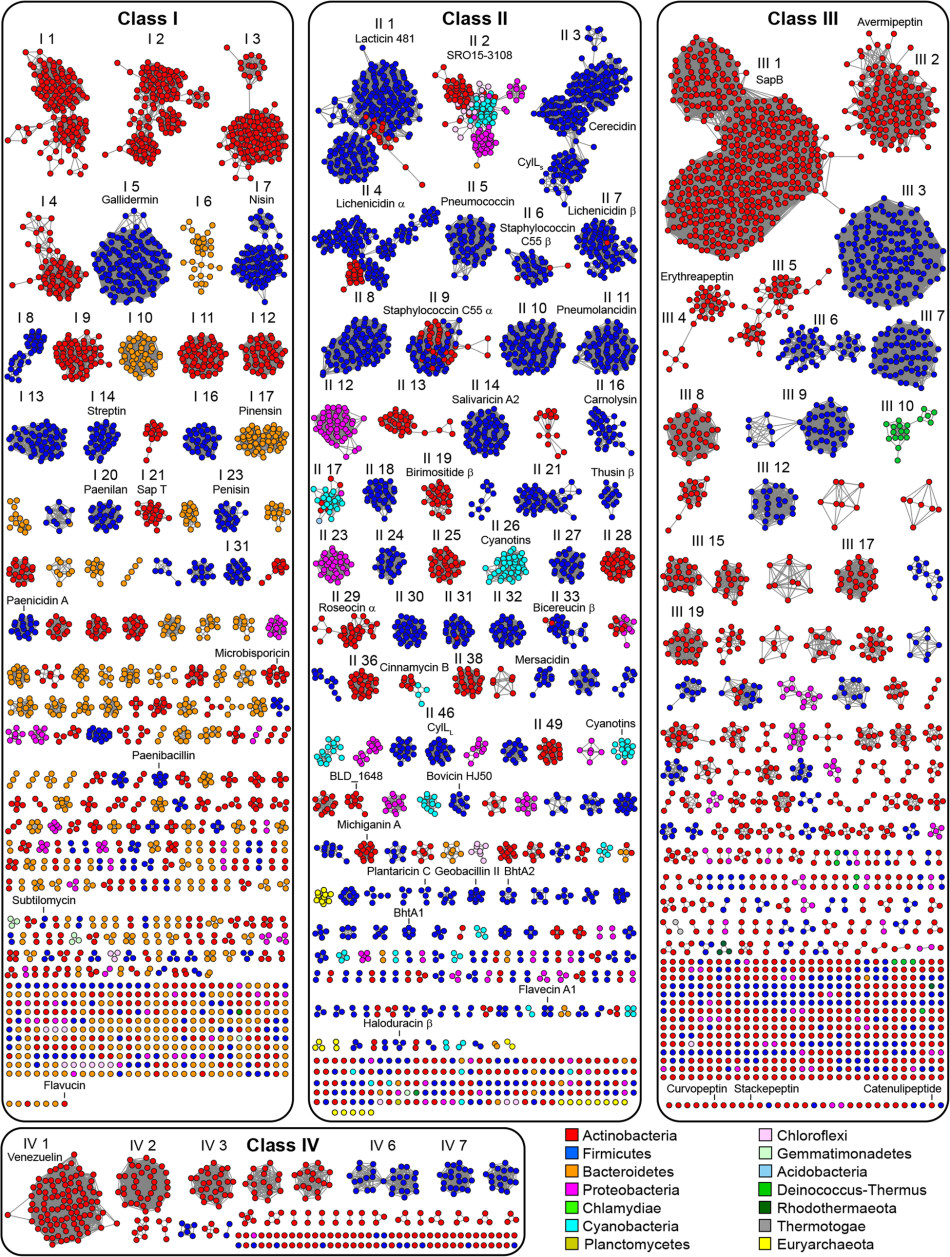
Sequence similarity networks [51] of precursor peptides. Clusters of precursor peptides with 20 or more members are numbered and sequence logos for these clusters are presented in Supplementary Figure S3. Clusters with characterized members as determined by using BAGEL4 [29] and the MIBiG repository [52] (Supplementary Table S5) are labeled by a selected member

A sequence similarity network analysis [51] (Fig. 2) reveals that the identified precursor peptides tend to cluster into families by lanthipeptide class and by taxonomic phylum (Fig. 2 and Supplementary Figure S3, Additional File 1). Even though a number of these families include lanthipeptides that have been characterized, as indicated by the representative lanthipeptides shown (Supplementary Table S5, Additional File 1), most families lack a characterized member, highlighting the scope of lanthipeptide sequence space that remains to be studied. In this work, we have labeled the precursor families by a Roman numeral indicating lanthipeptide class and an increasing Arabic number from left to right and top to bottom in the order generated by the Organic layout of Cytoscape [53]. Several of the uncharacterized families, including I 8, I 13, II 18, and II 32, appear to contain lipid II-binding motifs (Supplementary Figures S4, S5, S6, S7, Additional File 1) and are likely antibiotics. The four largest class I families (I 1–4) are from Actinobacteria and do not have a characterized member. Their core peptides contain a highly conserved Asp residue that is of particular note because the corresponding BGCs contain an *O*-methyltransferase (PF01135) and the conserved Asp is likely post-translationally modified [54]. A number of the class II families, such as II 2, II 13, II 17, and II 26 have conserved leader peptides and non-conserved core peptides. The leader peptides from families II 2, II 13, II 17, II 25, and II 29 belong to the nitrile hydratase leader peptide family of leader peptides, whereas the leader peptides from family II 26 belong to the Nif11 family of leader peptides [44]. The precursor peptides in family II 26 are from Cyanobacteria, however the prochlorosin lanthipeptides are not among them [49]. The prochlorosin precursor peptides (also in the Nif11 family) are located in a smaller cluster, which does not represent the actual size of this family of precursors as many of them are encoded in genes located distantly from their cognate LanM in the genome [49, 55] and thus were not identified in our analysis that limited the distance between the LanC-domain containing protein and the precursor peptide to seven genes upstream or downstream. We suggest the name cyanotins for this family of RiPPs that are made from highly diverse core peptides, some of which lack Cys and hence cannot be precursors to lanthipeptides.

### Other enzymes in lanthipeptide biosynthetic gene clusters

Very few of the BGCs with predicted precursors contained genes encoding class-defining enzymes from other lanthipeptide classes. For example, only six BGCs encoded a LanM and a LanB and LanC, and it is unclear if these encode a single biosynthetic pathway or two separate pathways encoded in close proximity. BGCs encoding a LanM and a LanKC have been identified previously [25]; however, the LanM-associated precursor peptides in those clusters lack Cys residues and therefore were not considered lanthipeptides in the current analysis. In contrast, enzymes that install secondary post-translational modifications are more broadly distributed. Other proteins present in the BGCs were characterized by searching the Pfam database of HMMs. Examining the most abundant proteins that hit at least one Pfam family reveals proteases, ABC transporters, and transcriptional regulators (Supplementary Tables S6, S7, S8, S9, Additional File 1). A number of class I BGCs contain split LanB enzymes that contain the glutamylation and elimination domains on separate polypeptides, as is seen in the biosynthesis of the lanthipeptide pinensin [6], as well as the thiopeptide family of RiPPs [56]. Other class I BGCs contain a full length LanB and an additional protein homologous to the LanB elimination domain. These proteins are also homologous to the enzyme in thiopeptide biosynthesis that catalyzes a formal [4 + 2] cycloaddition to install a substituted pyridine or (dehydro) piperidine moiety [14, 57–59]. Accordingly, it is an intriguing possibility that these domains catalyze a post-translational modification other than elimination. These standalone elimination domain proteins are also often fused to protein-L-isoaspartate *O*-methyltransferase (PCMT or PIMT, PF01135) family proteins and, in turn, many BGCs have these *O*-methyltransferases as standalone proteins. Notably, these elimination domain proteins and methyltransferases are nearly exclusively limited to class I BGCs (Supplementary Table S10, Additional File 1).

Enzymes that are among the most abundant in one class of lanthipeptide BGCs are generally also present in the other classes, if at lower abundance (Supplementary Table S10 and Figure S8, Additional File 1). For example, flavoprotein family enzymes, which have been shown to catalyze oxidative decarboxylation of the C-terminus of some lanthipeptides (LanDs) [60–64], halogenation of amino acid side chains [62], and oxidation of the sulfur in lanthionine crosslinks [65], are among the most abundant enzymes in class I BGCs but are present in class II and III BGCs as well. Likewise, NAD(P)H-dependent FMN reductase family enzymes, such as those that catalyze the reduction of dehydro amino acid side chains to form D-amino acid residues (LanJ_B_) [66, 67], are among the most common tailoring enzymes in class II BGCs and are present in class I and III BGCs. Another enzyme family, the zinc-dependent dehydrogenases, have been demonstrated to carry out the same overall reaction (LanJ_A_s) [68], and members of this family are present in all four classes of lanthipeptide BGCs (Supplementary Table S11). To date, the installation of D-amino acids has only been observed in class II lanthipeptides, but these reductases and dehydrogenases suggest these structures may also be present in class I, III, and IV lanthipeptides, or alternatively, these enzymes may catalyze a new post-translational modification. Some BGCs from all four classes of lanthipeptides encode a short chain dehydrogenase. This family of enzymes has been shown to install an N-terminal lactate moiety [69], although this modification has thus far only been observed in class I lanthipeptides. To date, no secondary post-translational modifications have been reported for class IV lanthipeptides; however, a number of these clusters contain genes encoding FAD-dependent oxidoreductases, glycosyltransferases, and acetyltransferases. Thus, tailoring may occur for the products of these clusters, or alternatively, the genes encoding these other enzymes may not be part of the gene clusters.

Many BGCs appear to encode enzymes that are less widely distributed but may carry out rare post-translational modifications (Supplementary Table S11 and Figure S9, Additional File 1). For example, some class I, II, and III lanthipeptide BGCs contain a YcaO family protein (PF02624), members of which catalyze modification to the amide backbone [70]. Moreover, a number of BGCs for all four classes of lanthipeptides encode polyketide or fatty acid biosynthetic machinery, as in the recently reported class III lipolanthine [63], or non-ribosomal peptide biosynthetic machinery. Enzymes from other families, such as radical SAM (PF04055), cytochrome P450 (PF00067), and α-ketoglutarate-dependent oxygenases (PF03171), are present in lanthipeptide BGCs and may catalyze the installation of additional secondary modifications. A number of these BGCs were previously identified in Actinobacteria [25], however the current analysis reveals they are present in numerous phyla, highlighting the broad distribution of lanthipeptide BGCs.

### Phylogenetic distribution of lanthipeptide biosynthetic gene clusters

**Fig. 3:**
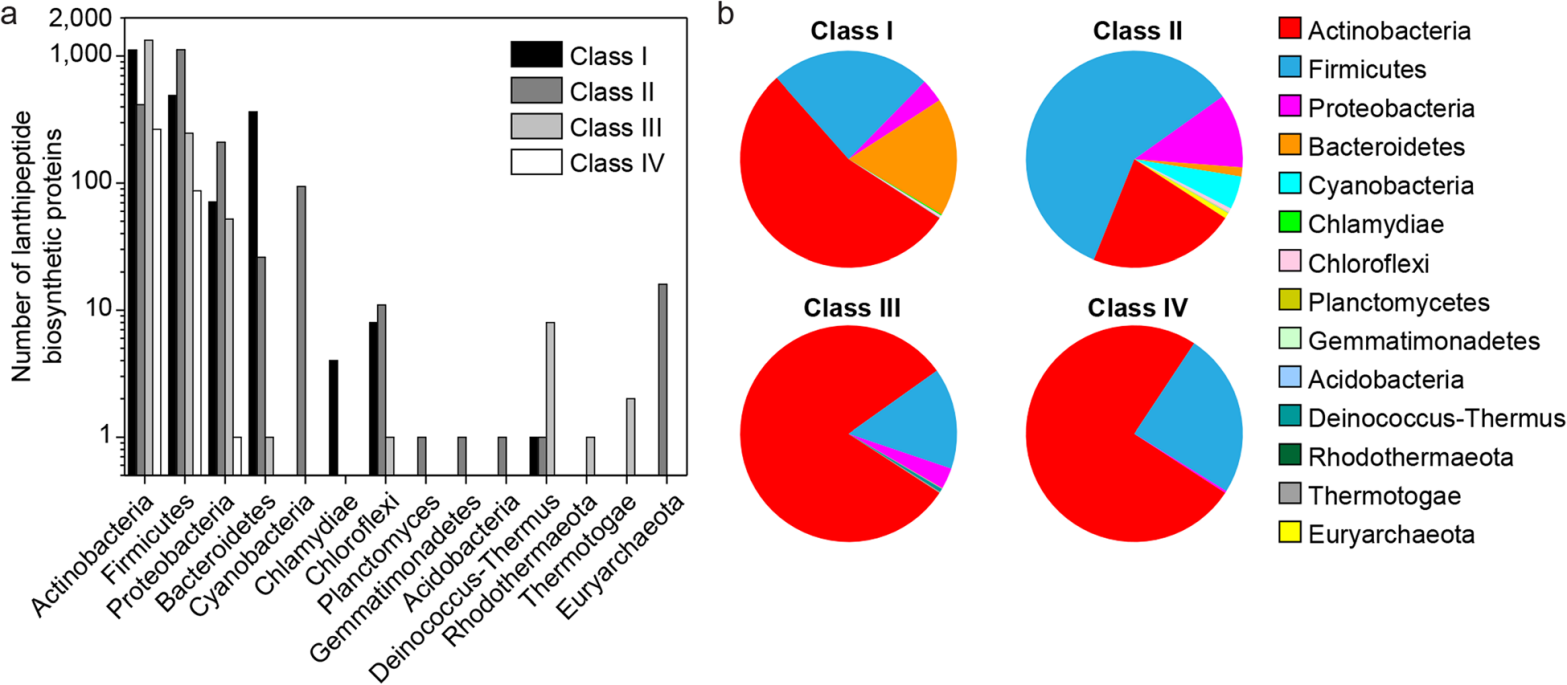
Phylogenetic distribution of lanthipeptide biosynthetic gene clusters. **a** The distribution of lanthipeptide BGCs in bacterial and archaeal phyla. **b** The distribution of bacterial and archaeal phyla among the four classes of lanthipeptide BGCs

Lanthipeptide biosynthetic enzymes were identified in a wide range of bacterial phyla, with the majority (within currently sequenced genomes) in Actinobacteria (Fig. 3a). The distribution of these proteins across phyla is inconsistent for the different classes of lanthipeptides (Fig. 3b). Nearly a quarter of the class I LanCs were identified in Bacteroidetes, despite their genomes making up a relatively small portion of those in the data set (Supplementary Figure S10, Additional File 1). This distribution suggests further genome sequencing efforts of Bacteroidetes may uncover additional novel lanthipeptide BGCs. At present, only the pinensins have been isolated from this phylum [6]. LanMs were the only lanthipeptide biosynthetic enzymes identified in Cyanobacteria. The majority of LanKCs and LanLs are from Actinobacteria and Firmicutes (Fig. 2); however, no members of these class III or class IV lanthipeptides from Firmicutes have been characterized to date.

**Fig. 4:**
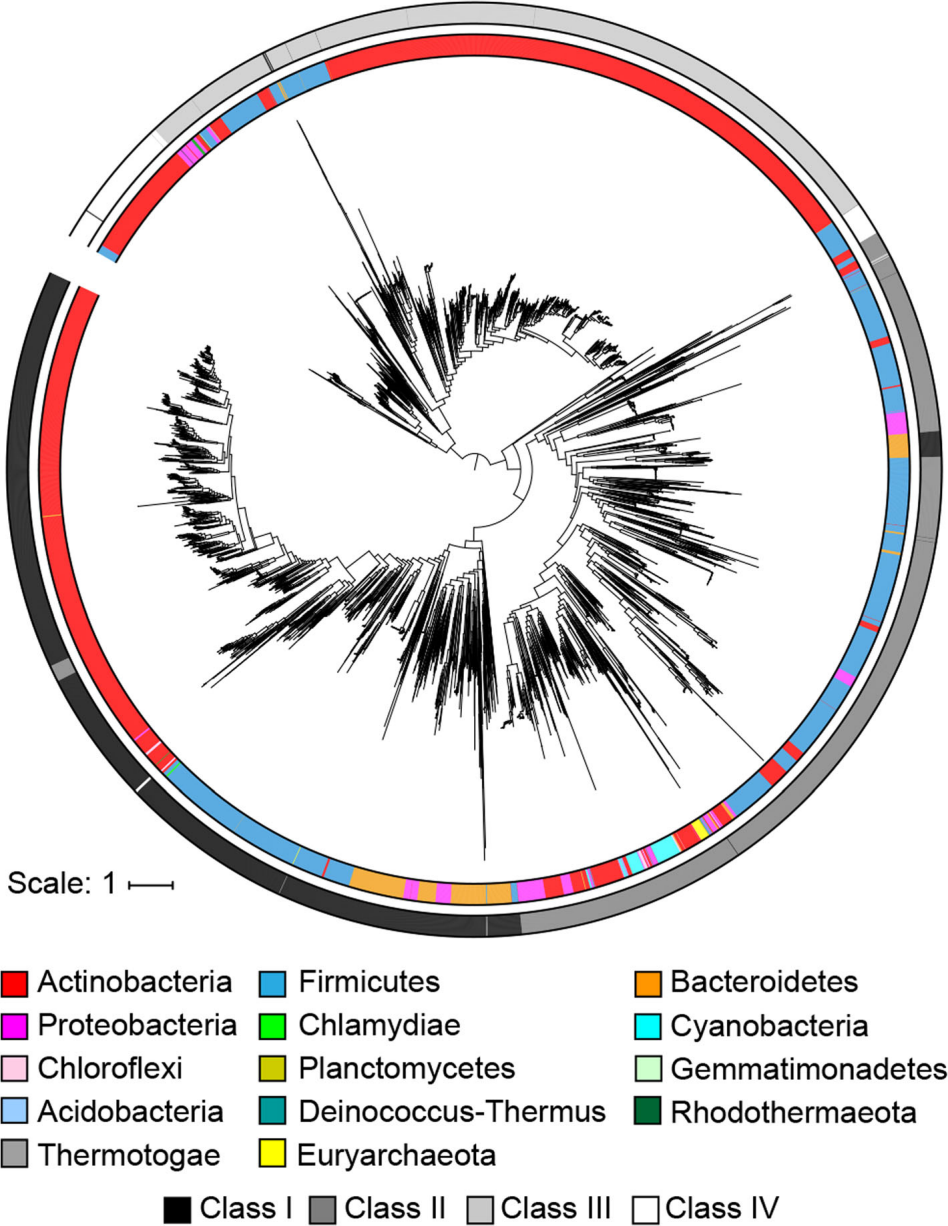
Phylogenetic analysis of LanC-like domains. A midpoint rooted approximately maximum likelihood phylogenetic tree of LanC-like domains from lanthipeptide biosynthetic enzymes. The inner ring is colored according to prokaryotic phylum and the outer ring is colored according to lanthipeptide class

A phylogenetic tree of LanCs and LanC-like domains reveals clades corresponding to the class of lanthipeptide and then sub-clades of bacterial phyla (Fig. 4). This topology suggests the divergence of the lanthipeptide classes is ancient and supports the hypothesis that the lanthipeptide synthases that produce different classes may have arisen through convergent evolution [71]. Inclusion of human LanC-like proteins on the tree shows that they fall into the class IV clade, which is made up of proteins with LanC-like domains linked to a kinase domain (Supplementary Figure S11, Additional File 1). Notably, human LanC-like proteins bind to kinases in various cell lines [72]. Some exceptions to grouping by class are observed, such as class I LanCs from Bacteroidetes that appear to be related to the LanC-like domains of class II LanMs from Firmicutes. The precursor peptides associated with these LanCs fall into family I 17, which includes the antifungal lanthipeptide pinensin [6]. Furthermore, a group of the LanC-like domains of LanMs from Actinobacteria are related to LanCs from the same phylum with the precursors associated with these LanMs falling in family II 28. Additionally, an analysis of the %GC content of the lanthipeptide BGCs versus the %GC content of the entire bacterial or archaeal genome was performed. Generally, these two values are in good agreement (Supplementary Figure S12, Additional File 1).

### Identification of a two-component lanthipeptide BGC from Streptomyces rimosus subsp. rimosus WC3908

**Fig. 5:**
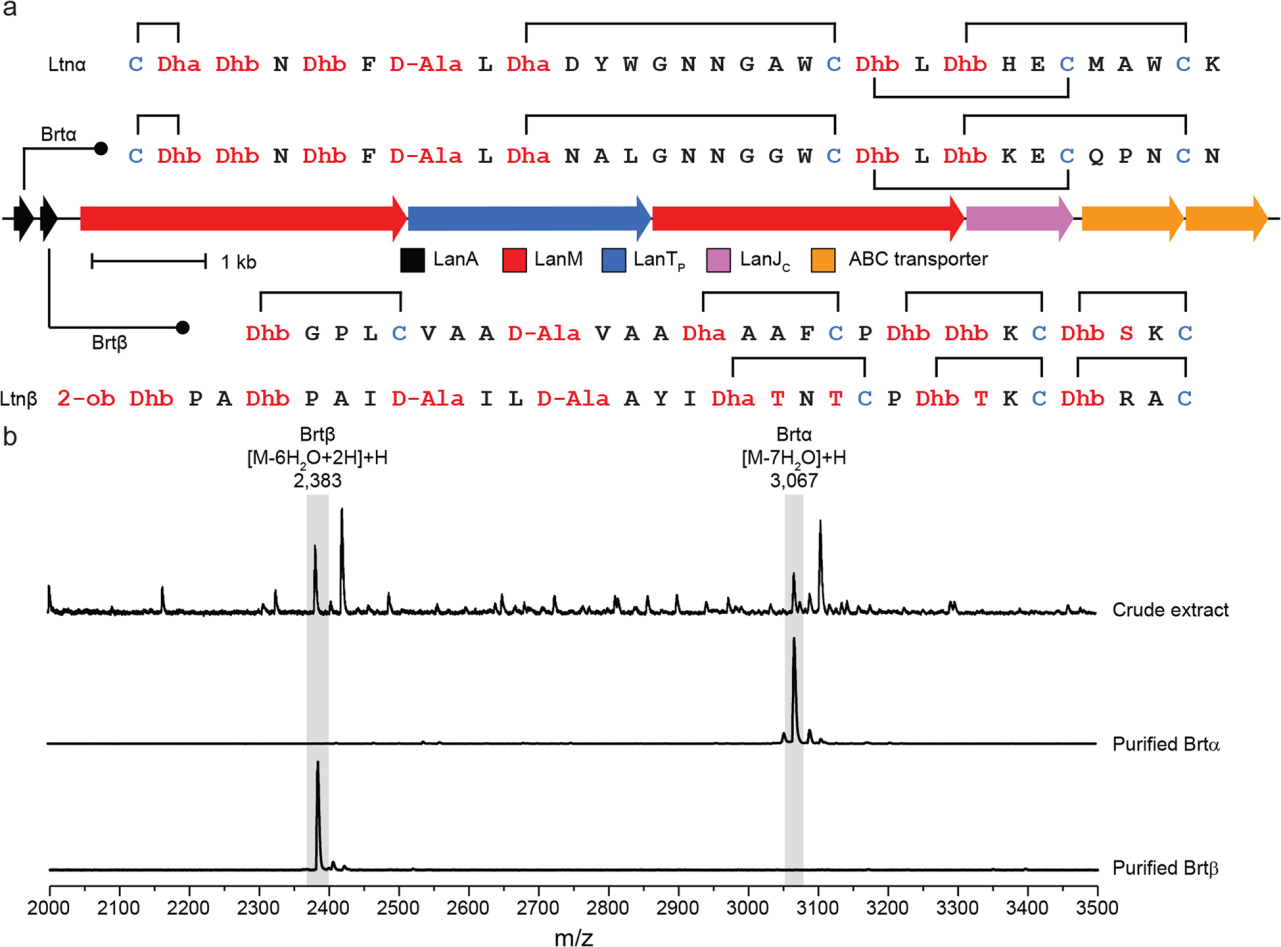
Characterization of a two-component lanthipeptide from *S. rimosus.***a** The biosynthetic gene cluster of birimositide and the predicted structures of Brtα and Brtβ with the cyclization pattern in comparison to those of lacticin 3147 (Ltnα and Ltnβ). The location of modified residues in Brtα and Brtβ are based on tandem mass spectrometry data (Supplementary Figure S13) and homology with lacticin 3147. Ser and Thr derived residues are colored in red as well as Ser and Thr, whereas Cys derived residues are colored in blue. Dha: dehydroalanine, Dhb: dehydrobutyrine, 2-ob: 2-oxo-butyrate moiety derived from Dhb. **b**) MALDI-TOF mass spectra showing Brtα and Brtβ in crude extract of *S. rimosus* culture, as well as purified Brtα and Brtβ. The masses correspond to the predicted core peptides dehydrated 7-fold (Brtα) and 6-fold (Brtβ) and the addition of hydrogen for the reduction of Brtβ

While several two-component lanthipeptides from Firmicutes have been characterized, only a single example, roseocin [73], has been reported from Actinobacteria. The roseocin α precursor peptide falls into family II 29 whereas the roseocin β precursor is a member of family II 2. To further explore two-component lanthipeptides from Actinobacteria, we focused on a BGC from *Streptomyces rimosus,* which encodes an α precursor peptide in family II 9 similar to lacticin 3147 α (Ltnα) [74, 75], and a β precursor peptide in family II 19 (Fig. 5a), the first example of a II 19 family member (Fig. 2). The BGC also encodes a flavin-dependent oxidoreductase belonging to the luciferase-like monooxygenase family (PF00296). The lacticin 3147 BGC encodes a LanJ_A_ that is involved in the conversion of Ser to D-Ala [68]. Curiously, whereas the corresponding Ser residues appear conserved in the precursor peptides encoded in the *S. rimosus* BGC (Fig. 5a), a gene encoding LanJ_A_ is not present. Therefore, we hypothesized that the luciferase-like monooxygenase might carry out a similar reaction. Upon culturing *Streptomyces rimosus*, masses corresponding to the predicted two-component lanthipeptide were observed and the purified peptides were shown to display the synergistic activity characteristic of a two-component lantibiotic (Supplementary Figure S13, Additional File 1). We named the compound birimositide and designated the locus encoding its biosynthesis *brt.* High-resolution mass spectra of both peptides were consistent with the conversion of one Ser to a D-Ala (Fig. 5b). Therefore, we propose the generic name LanJ_C_ to luciferase-like monooxygenases that reduce dehydrated residues in lanthipeptides. Tandem mass spectrometry suggests that the α-peptide (Brtα) is similar in structure to lacticin 3147 α including the position of the D-Ala, but that the β-peptide (Brtβ) is structurally more divergent from lacticin 3147 β (Ltnβ) and contains a single D-Ala compared to two Ser to D-Ala conversions for Ltnβ (Supplementary Figure S13 and Supplementary Table S12, Additional File 1).

## Discussion

In this work, we improved the ability of RODEO to predict precursor peptides for all four classes of lanthipeptides. This expanded functionality facilitated the mining of more than one hundred thousand bacterial and archaeal genomes for the ability to produce lanthipeptides. These studies revealed that lanthipeptide BGCs are more broadly distributed than previously appreciated, with a large number of class I lanthipeptides in Bacteroidetes, the presence of class III and IV lanthipeptides in Firmicutes, and the detection of class II lanthipeptide BGCs in archaea. Examining the precursor peptides encoded in the gene clusters revealed that the majority of lanthipeptide natural product families have not been characterized, including a number that are likely antibiotics because of lipid II-binding motifs. As delineated below, several new insights have been revealed through this bioinformatics study.

**Fig. 6:**
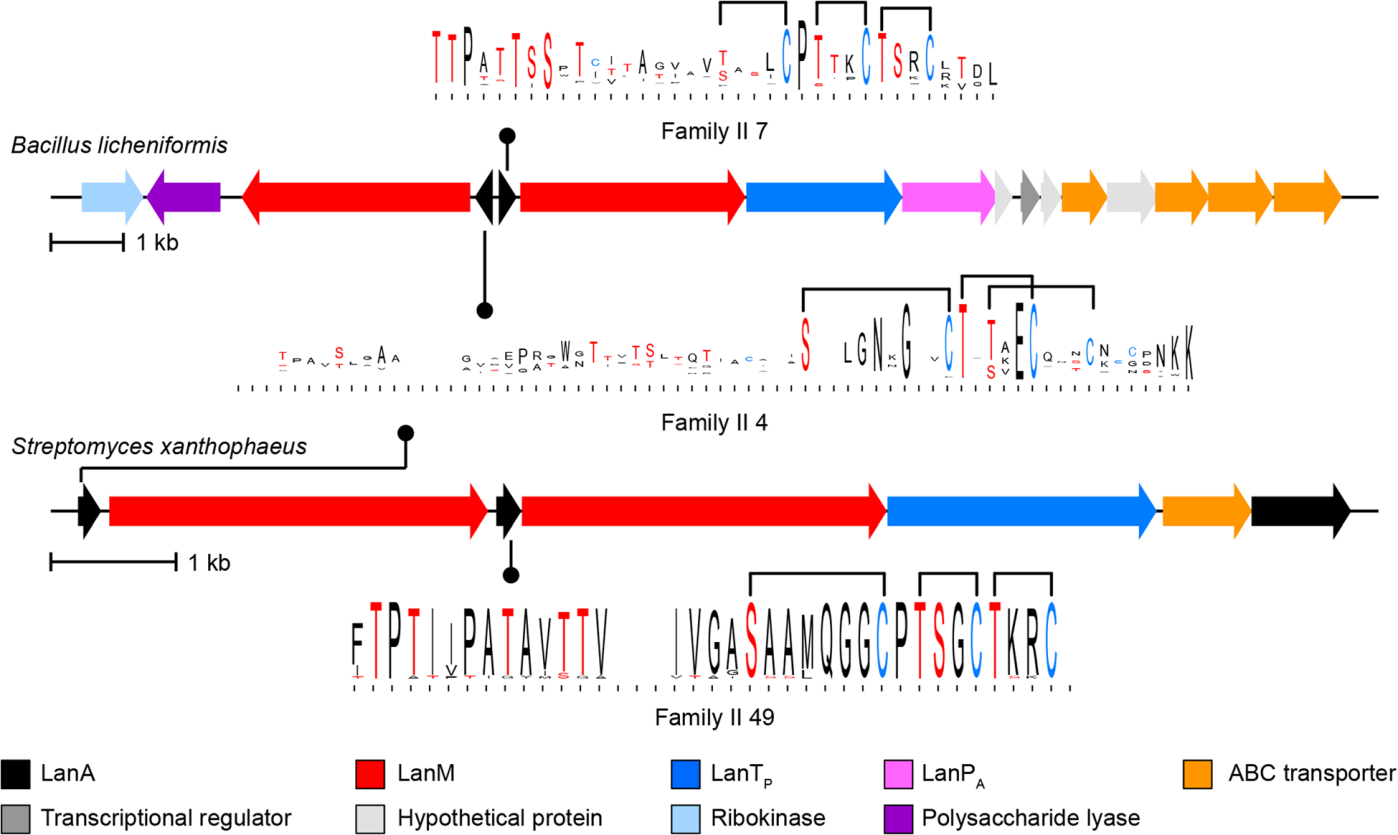
Representative diagrams for two-component lanthipeptide BGCs that share an α precursor peptide (from family II 4) with β peptides from families II 7 or II 49. Sequence logos for the predicted core peptides are shown as well. In the sequence logos, residues that can be dehydrated (i.e. Ser and Thr) are shown in red and Cys residues are shown in blue and predicted cyclization patterns are shown. The predicted cyclization pattern for family II 49 is based on similar positioning of modifiable residues in family II 7

As in a previous study that focused on Actinobacteria [25], the most common lanthipeptide precursor family when analyzing all currently available genomes from different phyla (III 1) is the morphogenic SapB peptide involved in sporulation [76]. The third- and fourth-most abundant precursor families (II 3 and II 4) comprise single- and two-component lanthipeptides in which two structurally dissimilar lanthipeptides exert synergistic bioactivity, with the individual peptides usually having low or no activity [77]. The fourth most abundant family (II 4) includes the α-peptide of the two-component lanthipeptides lichenicidin [30, 78, 79], haloduracin [80], and thusin [81] (Supplementary Table S5, Additional File 1), and are primarily found in Firmicutes with some members from Actinobacteria. Unexpectedly, the precursors of the partner lanthipeptide that would make up the two-component systems are not in one family but are more diverse in structure. In Firmicutes, lichenicidin β is a member of family II 7, and haloduracin β and thusin β are in small families, whereas the putative partners of the actinobacterial lichenicidin α-like precursors are in family II 49 (Fig. 6). Precursors related to other two-component lanthipeptides such as staphylococcin C55 and the newly reported birimositide also share a similar bifurcated distribution. The precursors of staphylococcin C55 α and birimositide α are members of family II 9, which is comprised of peptides encoded in Firmicutes and Actinobacteria. However, the precursor of staphylococcin C55 β is part of family II 6, whereas birimositide β (and other putative partners of the Actinobacterial α-peptides) are in family II 19. The α peptides of currently investigated two-component lanthipeptides are involved in lipid II-binding. The resulting complex is believed to serve as a binding site for the β peptides, which results in pore formation in the bacterial membranes [78, 82, 83]. The more divergent structures of the β peptides may suggest that different features are required to form pores in the membranes of the target bacteria for lanthipeptide producers that live in different ecological niches.

The precursor peptides of the third most abundant family of peptides (II 3) have sequence homology with the CylL_S_′′ peptide that together with CylL_L_′′ makes up the enterococcal cytolysin. This two-component lantibiotic lyses bacterial and mammalian cells [84], and epidemiological studies have shown a clear correlation between the presence of the cytolysin biosynthetic gene locus and hospital-acquired infections [85]. The precursor peptides of the CylL_L_′′ peptides (II 30) are much less abundant than the precursor peptides for CylL_S_′′ (II 3) (Fig. 2). Genes encoding these CylL_S_-like peptides are found mostly in species of *Bacillus* and sometimes in *Staphylococcus aureus*. Previous studies noted these peptides in *B. cereus* [23, 86] and several members have been isolated (cericidins) and shown to display antimicrobial activity without the need of a partner peptide, explaining why family II 3 is much larger than family II 30.

**Fig. 7:**
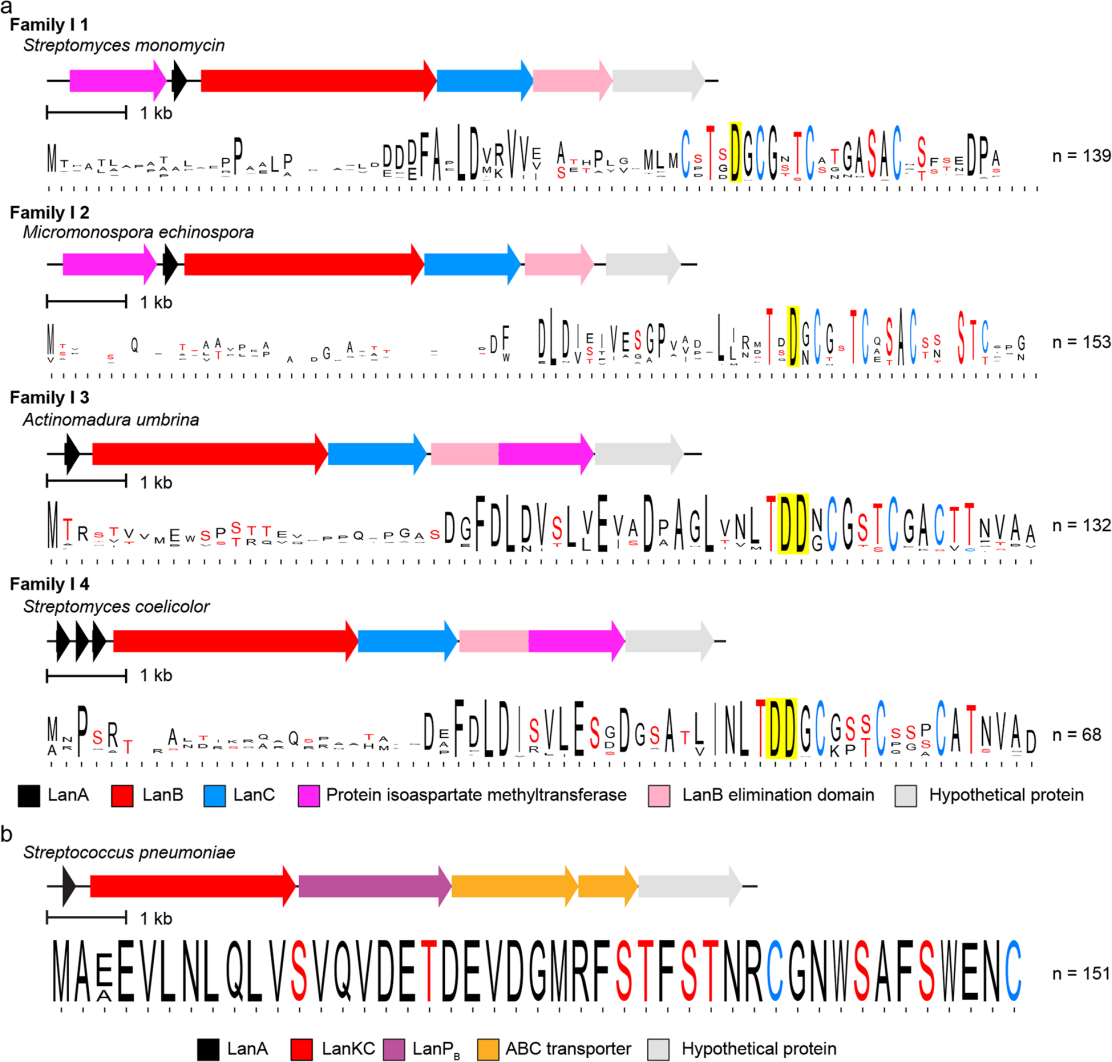
**a**. Representative diagrams for BGCs containing precursor peptides from families I 1, I 2, I 3, and I 4 along with sequence logos for those precursor families. Conserved Asp residues in the predicted core peptide are highlighted in yellow. **b**. A representative diagram for BGCs containing a precursor peptide from family III 3 along with the sequence logo for that family. LanP_B_ is a prolyl oligopeptidase family protease

The most abundant class I precursor peptides in Actinobacteria (I 1–4) are encoded in BGCs that contain the previously mentioned ortholog of a protein isoaspartate methyltransferase as well as a fully conserved Asp in the core peptide (Fig. 7a). This methyltransferase is the highest co-occurring protein with lanthionine biosynthetic enzymes (Additional File 1: Supplementary Table S9) even though it is only found in class I lanthipeptide BGCs. Recently, it was shown that one such enzyme methylated the conserved Asp in a precursor peptide of family I 2 encoded in *Streptomyces olivaceus* NRRL B-3009. Methylation led to the formation of a succinimide that was hydrolyzed to a mixture of aspartate and isoaspartate. The mature form of the natural product remains unknown [54].

The sixth most abundant precursor family (III 3) consists of class III lanthipeptides encoded by Firmicutes; currently class III peptides have only been isolated from Actinobacteria. At present, the organisms encoding family III 3 are restricted to *Streptococcus pneumoniae.* The precursor peptides display identical core sequences (Fig. 7b) that contain the characteristic Sx_2_Sx_3_C motif that in some class III lanthipeptides give rise to a lanthionine between the first Ser and last Cys of the motif, and in other peptides yield labionin [10, 43, 76, 87] (Fig. 7b). Unlike class III lanthipeptide gene clusters from Actinobacteria that usually do not contain a protease [88], the III 3 clusters encode for a prolyl oligopeptidase (we propose the name LanP_B_ for these with the name LanP_A_ given to the previously characterized group of subtilisin-like leader peptide proteases [13]) as well as a CAAX family protease [89]. The smaller III 6 family is also found in Firmicutes but the phylogenetic distribution is more varied as these BGCs are present in different *Bacillus* and *Staphylococcus* species. Like class III BGCs from Actinobacteria, they do not encode a conserved protease in the BGC, but they do encode an ABC-type transporter.

Generally speaking, a particular family of lanthipeptides is usually only produced by a single phylum, but some exceptions are notable. The lacticin 481 family (II 1) is mostly produced by Firmicutes, but some Actinobacteria also encode members. Nisin A, a commercially used food preservative, and related lanthipeptides are almost exclusively encoded in Firmicutes genomes, but our analysis revealed a single Actinobacterium (*Nocardia vaccinii* NBRC 15922) encoding a member of this antimicrobial family. After finding an example of a nisin-family lanthipeptide in Actinobacteria we manually examined the genomic context of highly similar LanB and LanC enzymes. Nisin-like precursor peptides were identified in these BGCs, however, they were encoded more than seven genes away from the *lanC* gene and therefore fell outside of the genome neighborhood analyzed in this study. Some lanthipeptides seem to have distributed nearly equally between Firmicutes and Actinobacteria, such as the α peptide of the two-component lantibiotic staphylococcin C55, as well as the II 2 family, which is found roughly equally in three phyla, Firmicutes, Actinobacteria, and Cyanobacteria. At present, the molecular structures of members of this family have yet to be reported. Examination of the Pfam families of other enzymes encoded in the BGCs revealed that some of the tailoring enzymes that were previously thought to be limited to a single lanthipeptide class are in fact distributed among multiple, if not all, classes of lanthipeptides.

## Conclusions

The current comprehensive analysis of bacterial and archaeal genomes for the presence of *lanC*-like genes combined with the new methodology to characterize the cognate precursor peptides reveal the diversity of the lanthipeptide family of RiPPs and the extent to which a large portion of chemical space remains to be explored. The current study will facilitate prioritization of genome-mining studies for novel structures, new synergistic lanthipeptide pairs, or lanthipeptides from genera currently not known to produce such compounds.

## Methods

### Bioinformatic mining for lanthipeptides

The non-redundant protein, GenBank, and nucleotide records for bacteria and archaea in the RefSeq collection were downloaded from NCBI in May of 2019. The non-redundant protein records were searched with the LanC-Like Pfam HMM (PF05147.12) [90] using HMMER3 [91] with the default settings. The GenBank and nucleotide records were parsed using the list of LanC-Like proteins. Proteins encoded within seven ORFs upstream and downstream of the LanC-Like protein were annotated by searching the Pfam HMM database using HMMER with an E-value cutoff of 1 × 10^− 5^. Gene clusters that encoded proteins that matched with the Lant_dehydr_N Pfam HMM (PF04738.13) and the Lant_dehydr_C Pfam HMM (PF14028.6) were classified as class I clusters. Gene clusters that encoded a protein that matched with the DUF4135 Pfam HMM (PF13575.6) were classified as class II clusters. Gene clusters that encoded a protein that matched both the LANC_Like Pfam HMM and the Pkinase Pfam HMM (PF00069.25) were classified as class III or class IV clusters. Gene clusters that did not encode proteins that matched these HMMs were discarded as unclassified. Custom HMMs were developed for class III and class IV LanC-Like domains. The sequences of representative LanKCs and LanLs were aligned with Clustal-Omega [92], and these alignments were manually truncated to include only the LanC-like domain. HMMER was then used to generate HMMs from these alignments (Additional Files 3 and 4). The LanC-like proteins in the class III and IV gene clusters were then searched against these custom HMMs, and classified as class III if the E-value for the match with the class III HMM was lower than that for the class IV HMM, and as class IV if the E-value for the match with the class IV HMM was lower than that for the class III HMM. Additionally, the DNA sequence spanning the most upstream ORF and most downstream ORF was translated into all potential ORFs with ATG, GTG, or TTG start codons. Potential gene products that were longer than 120 amino acids or shorter than 30 amino acids were discarded, as were any that were encoded entirely within an annotated gene. Additionally, any ORF not encoding a Cys was discarded as it could not be a potential lanthipeptide precursor. Finally, to reduce redundancy, the longest of the remaining ORFs with the same stop codon coordinates were retained for further analysis.

### Scoring of potential precursor peptides

Leader peptide motifs were identified in the gene products identified above using the MEME bioinformatics application. The leader-core boundary was then estimated by searching the amino acids following the leader peptide motif for GG, GA, or S/T(x)_2-7_C and setting the core region as starting immediately following a GG or GA motif or 1 residue before a S/T(x)_2-7_C as long as that motif was more than 10 residues from the end of the peptide. If multiple of these motifs were identified, the one allowing the longest core region was used as the boundary. If none of these motifs were present or were present within 10 residues of the C-terminus, the C-terminal half of the ORF was used as the core. If a leader peptide motif was not identified, the same analysis was performed from the beginning of the ORF. Finally, if no Cys was present in the estimated core region, the ORF was discarded as not a lanthipeptide precursor. Features were then calculated for the potential core peptide, SVM classification was performed, and the potential precursor peptides were scored according to the rubrics in Supplementary Tables S1, S2, S3, S4.

### Phylogenetic analysis of LanC and LanC-like domains

LanC and LanC-like domain containing proteins from clusters encoding likely precursor peptides per the analysis above, were retrieved. LanM, LanKC, and LanL enzymes were aligned separately using Clustal-Omega, manually truncated to their LanC-like domains, and then unaligned. Then an alignment of LanCs and LanC-like domains was constructed using Clustal-Omega and manually edited to remove large gaps. This alignment was used to calculate an approximately maximum likelihood phylogenetic tree using FastTree [93]. The tree was then visualized using the Interactive Tree of Life [94].

### Precursor sequence logos

Likely precursor peptides were aligned using Clustal-Omega, and that alignment was used to generate sequence logos using WebLogo [95].

### Purification of birimositide peptides

*Streptomyces rimosus* subsp*. rimosus* WC3908 was cultivated on GYM agar for 4 d at 30 °C. After growth, colonies were extracted with methanol. Solvent was removed under vacuum using rotary evaporation and the extract was resuspended in up to 10% MeCN:H_2_O prior to high-performance liquid chromatography (HPLC) purification. HPLC purification was conducted using a C18 column (Macherey-Nagel, 100 Å, 250 × 10 mm, 5 μm) connected to an Agilent Infinity II LC system with solvents A (0.1% trifluoroacetic acid in H_2_O) and B (0.1% trifluoroacetic acid in MeCN) at a flow rate of 4 mL/min. The following method was used: stationary at 2% B for 10 min, followed by a linear increase from 2 to 98% B over 35 min, and holding stationary at 98% B for an additional 2 min. For LC coupled to mass spectrometry (MS), LC was performed on HPLC-purified samples using a C18 column (Acclaim PepMap RSLC, nanoViper, 75 μm × 15 cm) on a Dionex UltiMate 3000 LC. The following stepwise linear gradient was applied with solvents A (1% formic acid in H_2_O) and B (1% formic acid in MeCN) at a flow rate of 0.3 μL/min: starting at 4% B to 35% B over 45 min, 35 to 50% B over 6 min, 50 to 75% B over 6 min, and holding stationary at 75% B for 6 min. Hybrid Ion Trap-Orbitrap Mass Spectrometer (Thermo Fusion Tribrid Orbitrap, Thermo Scientific) Peptides were diluted 1:1 with ESI mix (80% MeCN, 19% H_2_O, 1% formic acid) in H_2_O. Calibration and tuning was conducted with Pierce LTQ Velos ESI Positive Ion Calibration (ThermoFisher). Data were obtained using the following parameters: resolution, 30,000; isolation window (MS/MS), 1.6 m/z; collision-induced energy (CID) (MS/MS), 35%; CID activation time, 10 ms; activation q value (MS/MS), 0.25. Data analysis was performed with the Qualbrowser application of Xcalibur software (Thermo-Fisher Scientific).

### Birimositide BGC

The genome of *Streptomyces rimosus* subsp. *rimosus* WC3908 was sequenced as part of another genome mining effort focused on phosphonates [96]. The sequence of the birimositide BGC has been deposited under accession number MT037000.

### Bioactivity assay

Brtα and Brtβ were resuspended in 50% MeCN:H_2_O to a concentration of 50 μM. Each peptide was spotted at varying concentrations on 20 mL of Mueller-Hinton agar seeded with *Micrococcus luteus* ATCC 4698 or *Lactococcus lactis* sp*. cremoris* at a final OD_600_ of 0.05. Inhibition was assessed after overnight growth at 37 °C for *M. luteus* and 30 °C for *L. lactis*. Images were obtained using a GelDoc XR+ molecule imager (Bio-Rad).

## Supplementary information

**Additional file 1: Figure S1.** Features calculated to score precursor peptides; ** Table S1.** Features and scoring for class I precursors; ** Table S2.** Features and scoring for class II precursors; ** Table S3.** Features and scoring for class III precursors; ** Table S4.** Features and scoring for class IV precursors; ** Figure S2.** Sequence motifs present in more than 100 lanthipeptide precursor peptides; ** Figure S3.** Sequence similarity network of predicted precursor peptides with permissive similarity cutoff; ** Table S5.** Location of top BLAST hits of known lanthipeptides from the MIBiG and BAGEL databases in the sequence similarity networks presented in Fig. 2; ** Figure S4.** Sequence logos generated from alignments of class I precursor peptides in clusters with 20 or more members; ** Figure S5.** Sequence logos generated from alignments of class II precursor peptides in clusters with 20 or more members; ** Figure S6.** Sequence logos generated from alignments of class III precursor peptides in clusters with 20 or more members; ** Figure S7.** Sequence logos generated from alignments of class IV precursor peptides in clusters with 20 or more members; ** Table S6.** Twenty most abundant proteins in class I BGCs that belong to at least one Pfam; ** Table S7.** Twenty most abundant proteins in class II BGCs that belong to at least one Pfam; ** Table S8.** Twenty most abundant proteins in class III BGCs that belong to at least one Pfam; ** Table S9.** Twenty most abundant proteins in class IV BGCs that belong to at least one Pfam; ** Table S10.** Distribution of Pfams that are in the 20 most abundant protein families in one class among the other three classes; ** Figure S8.** Example biosynthetic gene clusters encoding the enzymes in Table S10; ** Table S11.** Distribution of select Pfam protein families from BGCs; ** Figure S9.** Example biosynthetic gene clusters encoding the enzymes in Table S11; ** Figure S10.** Phylogenetic distribution of genomes in the dataset; ** Figure S11.** A approximately maximum likelihood midpoint rooted phylogenetic tree of LanC and LanC-like domains including human LanC-like proteins; ** Figure S12.** GC content of clusters versus genomes; ** Figure S13.** ESI MS/MS and bioactivity of birimositide α and β; ** Table S12.** Expected and observed monoisotopic masses for Brtα and Brtβ using ESI MS.

**Additional file 2.** Excel File containing precursor peptides identified in this study.

**Additional file 3.** Custom HMM for LanKC LanC-like domains.

**Additional file 4.** Custom HMM for LanL LanC-like domains.

## Data Availability

All biosynthetic gene clusters and precursor peptides identified in this study are available in Additional file 2. Genomes used in this study are available from NCBI in RefSeq release 93. The software used to perform the genome-mining studies is available at https://github.com/mcwalker-group/reimagined-octo-funicular. The improved lanthipeptide precursor prediction has been incorporated into the RODEO web tool at http://ripp.rodeo and the command line version at https://gitgub.com/the-mitchell-lab/rodeo2.

## Abbreviations

BGC: Biosynthetic gene cluster
Dha: Dehydroalanine
Dhb: Dehydrobutyrine
HMM: Hidden Markov model
LanA: Lanthipeptide precursor peptide
LanB: Class I lanthipeptide dehydratase
LanC: Class I lanthipeptide cyclase
LanKC: Class III lanthipeptide synthetase
LanL: Class IV lanthipeptide synthetase
LanM: Class II lanthipeptide synthetase
MEME: Multiple em for motif elicitation
ORF: Open reading frame
Pfam: Protein family
RODEO: Rapid ORF description & evaluation online
RiPP: Ribosomally synthesized and post-translationally modified peptide
SVM: Support vector machine

## References

[1] Arnison PG, Bibb MJ, Bierbaum G, Bowers AA, Bugni TS, Bulaj G, Camarero JA, Campopiano DJ, Challis GL, Clardy J (2013). Ribosomally synthesized and post-translationally modified peptide natural products: overview and recommendations for a universal nomenclature. Nat Prod Rep.

[2] Jabés D, Brunati C, Candiani G, Riva S, Romanó G, Donadio S (2011). Efficacy of the new lantibiotic NAI-107 in experimental infections induced by multidrug-resistant gram-positive pathogens. Antimicrob Agents Chemother.

[3] Mathur H, O'Connor PM, Hill C, Cotter PD, Ross RP (2013). Analysis of anti-Clostridium difficile activity of thuricin CD, vancomycin, metronidazole, ramoplanin, and actagardine, both singly and in paired combinations. Antimicrob Agents Chemother.

[4] Goldstein BP, Wei J, Greenberg K, Novick R (1998). Activity of nisin against Streptococcus pneumoniae, in vitro, and in a mouse infection model. J Antimicrob Chemother.

[5] Hirsch A (1950). The assay of the antibiotic nisin. J Gen Microbiol.

[6] Mohr KI, Volz C, Jansen R, Wray V, Hoffmann J, Bernecker S, Wink J, Gerth K, Stadler M, Müller R (2015). Pinensins: the first antifungal lantibiotics. Angew Chem Int Ed.

[7] Férir G, Petrova MI, Andrei G, Huskens D, Hoorelbeke B, Snoeck R, Vanderleyden J, Balzarini J, Bartoschek S, Brönstrup M (2013). The lantibiotic peptide labyrinthopeptin A1 demonstrates broad anti-HIV and anti-HSV activity with potential for microbicidal applications. PLoS One.

[8] Smith TE, Pond CD, Pierce E, Harmer ZP, Kwan J, Zachariah MM, Harper MK, Wyche TP, Matainaho TK, Bugni TS (2018). Accessing chemical diversity from the uncultivated symbionts of small marine animals. Nat Chem Biol.

[9] Iorio M, Sasso O, Maffioli SI, Bertorelli R, Monciardini P, Sosio M, Bonezzi F, Summa M, Brunati C, Bordoni R (2014). A glycosylated, labionin-containing lanthipeptide with marked antinociceptive activity. ACS Chem Biol.

[10] Meindl K, Schmiederer T, Schneider K, Reicke A, Butz D, Keller S, Guhring H, Vertesy L, Wink J, Hoffmann H (2010). Labyrinthopeptins: a new class of carbacyclic lantibiotics. Angew Chem Int Ed.

[11] Kim SG, Becattini S, Moody TU, Shliaha PV, Littmann ER, Seok R, Gjonbalaj M, Eaton V, Fontana E, Amoretti L (2019). Microbiota-derived lantibiotic restores resistance against vancomycin-resistant *Enterococcus*. Nature.

[12] Duan Y, Llorente C, Lang S, Brandl K, Chu H, Jiang L, White RC, Clarke TH, Nguyen K, Torralba M (2019). Bacteriophage targeting of gut bacterium attenuates alcoholic liver disease. Nature.

[13] Repka LM, Chekan JR, Nair SK, van der Donk WA (2017). Mechanistic understanding of lanthipeptide biosynthetic enzymes. Chem Rev.

[14] Ortega MA, Hao Y, Zhang Q, Walker MC, van der Donk WA, Nair SK (2015). Structure and mechanism of the tRNA-dependent lantibiotic dehydratase NisB. Nature.

[15] Li B, Yu JP, Brunzelle JS, Moll GN, van der Donk WA, Nair SK (2006). Structure and mechanism of the lantibiotic cyclase involved in nisin biosynthesis. Science.

[16] Wang H, van der Donk WA (2012). Biosynthesis of the class III lantipeptide catenulipeptin. ACS Chem Biol.

[17] Dong SH, Tang W, Lukk T, Yu Y, Nair SK, van der Donk WA (2015). The enterococcal cytolysin synthetase has an unanticipated lipid kinase fold. eLife.

[18] Müller WM, Schmiederer T, Ensle P, Süssmuth RD (2010). In vitro biosynthesis of the prepeptide of type-III lantibiotic labyrinthopeptin A2 including formation of a C-C bond as a post-translational modification. Angew Chem Int Ed.

[19] Goto Y, Li B, Claesen J, Shi Y, Bibb MJ, van der Donk WA (2010). Discovery of unique lanthionine synthetases reveals new mechanistic and evolutionary insights. PLoS Biol.

[20] Funk MA, van der Donk WA (2017). Ribosomal natural products, tailored to fit. Acc Chem Res.

[21] Skinnider MA, Johnston CW, Edgar RE, Dejong CA, Merwin NJ, Rees PN, Magarvey NA (2016). Genomic charting of ribosomally synthesized natural product chemical space facilitates targeted mining. Proc Natl Acad Sci U S A.

[22] Letzel AC, Pidot SJ, Hertweck C (2014). Genome mining for ribosomally synthesized and post-translationally modified peptides (RiPPs) in anaerobic bacteria. BMC Genomics.

[23] Singh M, Sareen D (2014). Novel LanT associated lantibiotic clusters identified by genome database mining. PLoS One.

[24] Begley M, Cotter PD, Hill C, Ross RP (2009). Identification of a novel two-peptide lantibiotic, lichenicidin, following rational genome mining for LanM proteins. Appl Environ Microbiol.

[25] Zhang Q, Doroghazi JR, Zhao X, Walker MC, van der Donk WA (2015). Expanded natural product diversity revealed by analysis of lanthipeptide-like gene clusters in actinobacteria. Appl Environ Microbiol.

[26] Azevedo AC, Bento CB, Ruiz JC, Queiroz MV, Mantovani HC (2015). Distribution and genetic diversity of bacteriocin gene clusters in rumen microbial genomes. Appl Environ Microbiol.

[27] Marsh AJ, O'Sullivan O, Ross RP, Cotter PD, Hill C (2010). In silico analysis highlights the frequency and diversity of type 1 lantibiotic gene clusters in genome sequenced bacteria. BMC Genomics.

[28] Poorinmohammad N, Bagheban-Shemirani R, Hamedi J (2019). Genome mining for ribosomally synthesised and post-translationally modified peptides (RiPPs) reveals undiscovered bioactive potentials of actinobacteria. Antonie Van Leeuwenhoek.

[29] van Heel AJ, de Jong A, Song C, Viel JH, Kok J, Kuipers OP (2018). BAGEL4: a user-friendly web server to thoroughly mine RiPPs and bacteriocins. Nucleic Acids Res.

[30] Dischinger J, Josten M, Szekat C, Sahl HG, Bierbaum G (2009). Production of the novel two-peptide lantibiotic lichenicidin by *Bacillus licheniformis* DSM 13. PLoS One.

[31] Tracanna V, de Jong A, Medema MH, Kuipers OP (2017). Mining prokaryotes for antimicrobial compounds: from diversity to function. FEMS Microbiol Rev.

[32] Merwin NJ, Mousa WK, Dejong CA, Skinnider MA, Cannon MJ, Li HX, Dial K, Gunabalasingam M, Johnston C, Magarvey NA (2020). DeepRiPP integrates multiomics data to automate discovery of novel ribosomally synthesized natural products. Proc Natl Acad Sci U S A.

[33] Tietz JI, Schwalen CJ, Patel PS, Maxson T, Blair PM, Tai HC, Zakai UI, Mitchell DA (2017). A new genome-mining tool redefines the lasso peptide biosynthetic landscape. Nat Chem Biol.

[34] Finn RD, Coggill P, Eberhardt RY, Eddy SR, Mistry J, Mitchell AL, Potter SC, Punta M, Qureshi M, Sangrador-Vegas A (2016). The Pfam protein families database: towards a more sustainable future. Nucleic Acids Res.

[35] Bailey TL, Boden M, Buske FA, Frith M, Grant CE, Clementi L, Ren JY, Li WW, Noble WS (2009). MEME SUITE: tools for motif discovery and searching. Nucleic Acids Res.

[36] Hsu ST, Breukink E, Tischenko E, Lutters MA, De Kruijff B, Kaptein R, Bonvin AM, Van Nuland NA (2004). The nisin-lipid II complex reveals a pyrophosphate cage that provides a blueprint for novel antibiotics. Nat Struct Mol Biol.

[37] Hsu ST, Breukink E, Bierbaum G, Sahl HG, de Kruijff B, Kaptein R, van Nuland NA, Bonvin AM (2003). NMR study of mersacidin and lipid II interaction in dodecylphosphocholine micelles. Conformational changes are a key to antimicrobial activity. J Biol Chem.

[38] Szekat C, Jack RW, Skutlarek D, Farber H, Bierbaum G (2003). Construction of an expression system for site-directed mutagenesis of the lantibiotic mersacidin. Appl Environ Microbiol.

[39] van der Meer JR, Rollema HS, Siezen RJ, Beerthuyzen MM, Kuipers OP, de Vos WM (1994). Influence of amino acid substitutions in the nisin leader peptide on biosynthesis and secretion of nisin by *Lactococcus lactis*. J Biol Chem.

[40] Håvarstein LS, Diep DB, Nes IF (1995). A family of bacteriocin ABC transporters carry out proteolytic processing of their substrates concomitant with export. Mol Microbiol.

[41] Bobeica SC, Dong SH, Huo L, Mazo N, McLaughlin MI, Jimenez-Oses G, Nair SK, van der Donk WA. Insights into AMS/PCAT transporters from biochemical and structural characterization of a double Glycine motif protease. eLife. 2019;8. 10.7554/eLife.42305 PMC6363468 30638446

[42] Dirix G, Monsieurs P, Marchal K, Vanderleyden J, Michiels J (2004). Screening genomes of gram-positive bacteria for double-glycine-motif-containing peptides. Microbiology.

[43] Völler GH, Krawczyk JM, Pesic A, Krawczyk B, Nachtigall J, Süssmuth RD (2012). Characterization of new class III lantibiotics-erythreapeptin, avermipeptin and griseopeptin from *Saccharopolyspora erythraea*, *Streptomyces avermitilis* and *Streptomyces griseus* demonstrates stepwise N-terminal leader processing. ChemBioChem.

[44] Haft DH, Basu MK, Mitchell DA (2010). Expansion of ribosomally produced natural products: a nitrile hydratase- and Nif11-related precursor family. BMC Biol.

[45] Patton GC, Paul M, Cooper LE, Chatterjee C, van der Donk WA (2008). The importance of the leader sequence for directing lanthionine formation in lacticin 481. Biochemistry.

[46] Müller WM, Ensle P, Krawczyk B, Süssmuth RD (2011). Leader peptide-directed processing of labyrinthopeptin A2 precursor peptide by the modifying enzyme LabKC. Biochemistry.

[47] Blin K, Shaw S, Steinke K, Villebro R, Ziemert N, Lee SY, Medema MH, Weber T (2019). antiSMASH 5.0: updates to the secondary metabolite genome mining pipeline. Nucleic Acids Res.

[48] Blin K, Kazempour D, Wohlleben W, Weber T. Improved lanthipeptide detection and prediction for antiSMASH. PLoS One. 2014;9(7):e103665. 10.1371/journal.pone.0089420 PMC3930743 24586765

[49] Li B, Sher D, Kelly L, Shi Y, Huang K, Knerr PJ, Joewono I, Rusch D, Chisholm SW, van der Donk WA (2010). Catalytic promiscuity in the biosynthesis of cyclic peptide secondary metabolites in planktonic marine cyanobacteria. Proc Natl Acad Sci U S A.

[50] Zhao X, van der Donk WA (2016). Structural characterization and bioactivity analysis of the two-component lantibiotic Flv system from a ruminant bacterium. Cell Chem Biol.

[51] Gerlt JA, Bouvier JT, Davidson DB, Imker HJ, Sadkhin B, Slater DR, Whalen KL (2015). Enzyme function initiative-enzyme similarity tool (EFI-EST): a web tool for generating protein sequence similarity networks. Biochim Biophys Acta.

[52] Medema MH, Kottmann R, Yilmaz P, Cummings M, Biggins JB, Blin K, de Bruijn I, Chooi YH, Claesen J, Coates RC (2015). Minimum information about a biosynthetic gene cluster. Nat Chem Biol.

[53] Shannon P, Markiel A, Ozier O, Baliga NS, Wang JT, Ramage D, Amin N, Schwikowski B, Ideker T (2003). Cytoscape: a software environment for integrated models of biomolecular interaction networks. Genome Res.

[54] Acedo JZ, Bothwell IR, An L, Trouth A, Frazier C, van der Donk WA (2019). O-methyltransferase-mediated incorporation of a β-amino acid in Lanthipeptides. J Am Chem Soc.

[55] Cubillos-Ruiz A, Berta-Thompson JW, Becker JW, van der Donk WA, Chisholm SW (2017). Evolutionary radiation of lanthipeptides in marine cyanobacteria. Proc Natl Acad Sci U S A.

[56] Hudson GA, Zhang Z, Tietz JI, Mitchell DA, van der Donk WA (2015). In vitro biosynthesis of the core scaffold of the thiopeptide thiomuracin. J Am Chem Soc.

[57] Wever WJ, Bogart JW, Baccile JA, Chan AN, Schroeder FC, Bowers AA (2015). Chemoenzymatic synthesis of thiazolyl peptide natural products featuring an enzyme-catalyzed formal [4 + 2] cycloaddition. J Am Chem Soc.

[58] Zhang Z, Hudson GA, Mahanta N, Tietz JI, van der Donk WA, Mitchell DA (2016). Biosynthetic timing and substrate specificity for the Thiopeptide Thiomuracin. J Am Chem Soc.

[59] Cogan DP, Hudson GA, Zhang Z, Pogorelov TV, van der Donk WA, Mitchell DA, Nair SK (2017). Structural insights into enzymatic [4+2] aza-cycloaddition in thiopeptide antibiotic biosynthesis. Proc Natl Acad Sci U S A.

[60] Kupke T, Stevanovic S, Sahl HG, Götz F (1992). Purification and characterization of EpiD, a flavoprotein involved in the biosynthesis of the lantibiotic epidermin. J Bacteriol.

[61] Majer F, Schmid DG, Altena K, Bierbaum G, Kupke T (2002). The flavoprotein MrsD catalyzes the oxidative decarboxylation reaction involved in formation of the peptidoglycan biosynthesis inhibitor mersacidin. J Bacteriol.

[62] Ortega MA, Cogan DP, Mukherjee S, Garg N, Li B, Thibodeaux GN, Maffioli S, Donadio S, Sosio M, Escano J, et al. Two flavoenzymes install 5-chlorotryptophan and 2-aminovinyl cysteine during the biosynthesis of the lantibiotic NAI-107. ACS Chem Biol. 2017;12(2):548–57. 10.1021/acschembio.6b01031 PMC5315687 28032983

[63] Wiebach V, Mainz A, Siegert MJ, Jungmann NA, Lesquame G, Tirat S, Dreux-Zigha A, Aszodi J, Le Beller D, Süssmuth RD (2018). The anti-staphylococcal lipolanthines are ribosomally synthesized lipopeptides. Nat Chem Biol.

[64] Mo T, Yuan H, Wang F, Ma S, Wang J, Li T, Liu G, Yu S, Tan X, Ding W (2019). Convergent evolution of the Cys decarboxylases involved in aminovinyl-cysteine (AviCys) biosynthesis. FEBS Lett.

[65] Shi Y, Bueno A, van der Donk WA (2012). Heterologous production of the lantibiotic Ala (0) actagardine in *Escherichia coli*. Chem Commun.

[66] Lohans CT, Li JL, Vederas JC (2014). Structure and biosynthesis of carnolysin, a homologue of enterococcal cytolysin with D-amino acids. J Am Chem Soc.

[67] Huo L, van der Donk WA (2016). Discovery and characterization of bicereucin, an unusual D-amino acid-containing mixed two-component lantibiotic. J Am Chem Soc.

[68] Cotter PD, O'Connor PM, Draper LA, Lawton EM, Deegan LH, Hill C, Ross RP (2005). Posttranslational conversion of L-serines to D-alanines is vital for optimal production and activity of the lantibiotic lacticin 3147. Proc Natl Acad Sci U S A.

[69] Velásquez JE, Zhang X, van der Donk WA (2011). Biosynthesis of the antimicrobial peptide epilancin 15X and its N-terminal lactate moiety. Chem Biol.

[70] Burkhart BJ, Schwalen C, Mann G, Naismith JH, Mitchell DA (2017). YcaO-dependent posttranslational amide activation: biosynthesis, structure, and function. Chem Rev.

[71] Zhang Q, Yu Y, Velásquez JE, van der Donk WA (2012). Evolution of lanthipeptide synthetases. Proc Natl Acad Sci U S A.

[72] Zeng M, van der Donk WA, Chen J (2014). Lanthionine synthetase C-like protein 2 (LanCL2) is a novel regulator of Akt. Mol Biol Cell.

[73] Singh M, Chaudhary S, Sareen D. Roseocin, a novel two-component lantibiotic from an actinomycete. Mol Microbiol. 2020;113(2):326–37. 10.1111/mmi.14419 31696567

[74] Ryan MP, Jack RW, Josten M, Sahl HG, Jung G, Ross RP, Hill C (1999). Extensive post-translational modification, including serine to D-alanine conversion, in the two-component lantibiotic, lacticin 3147. J Biol Chem.

[75] Martin NI, Sprules T, Carpenter MR, Cotter PD, Hill C, Ross RP, Vederas JC (2004). Structural characterization of lacticin 3147, a two-peptide lantibiotic with synergistic activity. Biochemistry.

[76] Kodani S, Hudson ME, Durrant MC, Buttner MJ, Nodwell JR, Willey JM (2004). The SapB morphogen is a lantibiotic-like peptide derived from the product of the developmental gene *ramS* in *Streptomyces coelicolor*. Proc Natl Acad Sci U S A.

[77] Garneau S, Martin NI, Vederas JC (2002). Two-peptide bacteriocins produced by lactic acid bacteria. Biochimie.

[78] Shenkarev ZO, Finkina EI, Nurmukhamedova EK, Balandin SV, Mineev KS, Nadezhdin KD, Yakimenko ZA, Tagaev AA, Temirov YV, Arseniev AS (2010). Isolation, structure elucidation, and synergistic antibacterial activity of a novel two-component lantibiotic lichenicidin from *Bacillus licheniformis* VK21. Biochemistry.

[79] Caetano T, Krawczyk JM, Mosker E, Süssmuth RD, Mendo S (2011). Heterologous expression, biosynthesis, and mutagenesis of type II lantibiotics from *Bacillus licheniformis* in *Escherichia coli*. Chem Biol.

[80] McClerren AL, Cooper LE, Quan C, Thomas PM, Kelleher NL, van der Donk WA (2006). Discovery and in vitro biosynthesis of haloduracin, a two-component lantibiotic. Proc Natl Acad Sci U S A.

[81] Xin BY, Zheng JS, Liu HL, Li JH, Ruan LF, Peng DH, Sajid M, Sun M. Thusin, a novel two-component lantibiotic with potent antimicrobial activity against several gram-positive pathogens. Front Microbiol. 2016;7. 10.3389/fmicb.2016.01115 PMC4949975 27486447

[82] Wiedemann I, Bottiger T, Bonelli RR, Wiese A, Hagge SO, Gutsmann T, Seydel U, Deegan L, Hill C, Ross P (2006). The mode of action of the lantibiotic lacticin 3147--a complex mechanism involving specific interaction of two peptides and the cell wall precursor lipid II. Mol Microbiol.

[83] Oman TJ, Lupoli TJ, Wang T-SA, Kahne D, Walker S, van der Donk WA (2011). Haloduracin a binds the peptidoglycan precursor lipid II with 2:1 stoichiometry. J Am Chem Soc.

[84] Coburn PS, Gilmore MS (2003). The Enterococcus faecalis cytolysin: a novel toxin active against eukaryotic and prokaryotic cells. Cell Microbiol.

[85] Cox CR, Coburn PS, Gilmore MS (2005). Enterococcal cytolysin: a novel two component peptide system that serves as a bacterial defense against eukaryotic and prokaryotic cells. Curr Protein Pept Sci.

[86] Wang J, Zhang L, Teng K, Sun S, Sun Z, Zhong J (2014). Cerecidins, novel lantibiotics from *Bacillus cereus* with potent antimicrobial activity. Appl Environ Microbiol.

[87] Krawczyk B, Völler GH, Völler J, Ensle P, Süssmuth RD (2012). Curvopeptin: a new lanthionine-containing class III lantibiotic and its co-substrate promiscuous synthetase. ChemBioChem.

[88] Chen S, Xu B, Chen E, Wang J, Lu J, Donadio S, Ge H, Wang H (2019). Zn-dependent bifunctional proteases are responsible for leader peptide processing of class III lanthipeptides. Proc Natl Acad Sci U S A.

[89] Maxson T, Deane CD, Molloy EM, Cox CL, Markley AL, Lee SW, Mitchell DA (2015). HIV protease inhibitors block streptolysin S production. ACS Chem Biol.

[90] El-Gebali S, Mistry J, Bateman A, Eddy SR, Luciani A, Potter SC, Qureshi M, Richardson LJ, Salazar GA, Smart A (2019). The Pfam protein families database in 2019. Nuc Acids Res.

[91] Eddy SR (2011). Accelerated profile HMM searches. PLoS Comput Biol.

[92] Sievers F, Higgins DG (2018). Clustal omega for making accurate alignments of many protein sequences. Protein Sci.

[93] Price MN, Dehal PS, Arkin AP. FastTree 2-Approximately maximum-likelihood trees for large alignments. PLoS One. 2010;5(3):e9490. 10.1371/journal.pone.0009490 PMC2835736 20224823

[94] Letunic I, Bork P (2019). Interactive tree of life (iTOL) v4: recent updates and new developments. Nuc Acids Res.

[95] Crooks GE, Hon G, Chandonia JM, Brenner SE (2004). WebLogo: a sequence logo generator. Genome Res.

[96] Ju KS, Gao J, Doroghazi JR, Wang KK, Thibodeaux CJ, Li S, Metzger E, Fudala J, Su J, Zhang JK (2015). Discovery of phosphonic acid natural products by mining the genomes of 10,000 actinomycetes. Proc Natl Acad Sci U S A.

