# Supplementary material for: Precursor peptide-targeted mining of more than one hundred thousand genomes expands the lanthipeptide natural product family
Source: BMC Genomics. 2020 Jun 3;21:387. doi: 10.1186/s12864-020-06785-7 (PMC7268733; doi:10.1186/s12864-020-06785-7)
Supplement: Supplementary file 1 — Additional file 1: Figure S1. Features calculated to score precursor peptides; Table S1. Features and scoring for class I precursors; Table S2. Features and scoring for class II precursors; Table S3. Features and scoring for class III precursors; Table S4. Features and scoring for class IV precursors; Figure S2. Sequence motifs present in more than 100 lanthipeptide precursor peptides; Figure S3. Sequence similarity network of predicted precursor peptides with permissive similarity cutoff; Table S5. Location of top BLAST hits of known lanthipeptides from the MIBiG and BAGEL databases in the sequence similarity networks presented in Fig. 2; Figure S4. Sequence logos generated from alignments of class I precursor peptides in clusters with 20 or more members; Figure S5. Sequence logos generated from alignments of class II precursor peptides in clusters with 20 or more members; Figure S6. Sequence logos generated from alignments of class III precursor peptides in clusters with 20 or more members; Figure S7. Sequence logos generated from alignments of class IV precursor peptides in clusters with 20 or more members; Table S6. Twenty most abundant proteins in class I BGCs that belong to at least one Pfam; Table S7. Twenty most abundant proteins in class II BGCs that belong to at least one Pfam; Table S8. Twenty most abundant proteins in class III BGCs that belong to at least one Pfam; Table S9. Twenty most abundant proteins in class IV BGCs that belong to at least one Pfam; Table S10. Distribution of Pfams that are in the 20 most abundant protein families in one class among the other three classes; Figure S8. Example biosynthetic gene clusters encoding the enzymes in Table S10; Table S11. Distribution of select Pfam protein families from BGCs; Figure S9. Example biosynthetic gene clusters encoding the enzymes in Table S11; Figure S10. Phylogenetic distribution of genomes in the dataset; Figure S11. A approximately maximum likelihood midpoint rooted phylogenetic tree of Lan [file 12864_2020_6785_MOESM1_ESM.pdf]

Supporting Information for

Precursor peptide-targeted mining of more than one hundred thousand genomes expands the lanthipeptide natural product family

Mark C. Walker,[1,2]* Sara Eslami[2], Kenton J. Hetrick[2], Sarah E. Ackenhusen[2], Douglas A. Mitchell,[2,3,4] Wilfred A. van der Donk[2,3,5]

[1]Department of Chemistry and Chemical Biology, University of New Mexico, 346 Clark Hall, 300 Terrace St. NE, Albuquerque, New Mexico 87131, United States.

[2]Department of Chemistry, University of Illinois at Urbana-Champaign, Roger Adams Laboratory, 600 S. Mathews Ave., Urbana, Illinois 61801, United States.

[3]Carl R. Woese Institute for Genomic Biology, University of Illinois, Urbana, Illinois 61801, United States.

[4]Department of Microbiology, University of Illinois at Urbana-Champaign, 601 S. Goodwin Ave., Urbana, Illinois 61801, United States.

[5]Howard Hughes Medical Institute, University of Illinois at Urbana-Champaign, 600 S. Mathews Ave., Urbana, Illinois 61801, United States.

*To whom correspondence should be addressed. E-mail: markcwalker@unm.edu

ORCID:

Mark C. Walker	0000-0003-3942-003X
Sara M. Eslami	0000-0003-3397-0583
Kenton J. Hetrick	0000-0003-4657-5275
Sarah E. Ackenhusen	0000-0002-7412-575X
Douglas A. Mitchell	0000-0002-9564-0953
Wilfred A. van der Donk	0000-0002-5467-7071

Supplementary Figures and Tables

Supplementary Figure S1. Features calculated for precursor peptides 3
Supplementary Table S1. Features and scoring for class I precursors 4
Supplementary Table S2. Features and scoring for class II precursors 4
Supplementary Table S3. Features and scoring for class III precursors 4
Supplementary Table S4. Features and scoring for class IV precursors 4
Supplementary Figure S2. Sequence motifs in precursor peptides 5
Supplementary Figure S3. Sequence similarity network of precursor peptides 6
Supplementary Table S5. Location of characterized lanthipeptides in sequence similarity network 7
Supplementary Figure S4. Sequence logos of class I precursor families 8
Supplementary Figure S5. Sequence logos of class II precursor families 10
Supplementary Figure S6. Sequence logos of class III precursor families 12
Supplementary Figure S7. Sequence logos of class IV precursor families 14
Supplementary Table S6. Abundant protein families in class I biosynthetic gene clusters 15
Supplementary Table S7. Abundant protein families in class II biosynthetic gene clusters 15
Supplementary Table S8. Abundant protein families in class III biosynthetic gene clusters 16

Supplementary Table S9. Abundant protein families in class IV biosynthetic gene clusters 16
Supplementary Table S10. Distribution of abundant protein families among lanthipeptide biosynthetic gene clusters 17
Supplementary FigureS8. Representative lanthipeptide biosynthetic gene clusters with abundant protein families 18
Supplementary Table S11. Distribution of select protein families in lanthipeptide biosynthetic gene clusters 26
Supplementary Figure S9. Representative lanthipeptide biosynthetic gene clusters with select protein families 27
Supplementary Figure S10. Phylogenetic distribution of genomes used in this study 33
Supplementary Figure S11. Phylogenetic tree of LanC-like proteins including human LanC-like proteins 34
Supplementary Figure S12. GC content of biosynthetic gene clusters and their cognate genomes 35
Supplementary Figure S13. ESI MS/MS and bioactivity of birimositide α and β 36
Supplementary Table S12. Expected and observed monoisotopic masses for Brtα and Brtβ 37
References. 38

Supplementary Figure S1. Features calculated to score precursor peptides. The DNA sequence encoding 7 genes upstream and downstream of a LanC-like domain containing protein was extracted and all potential open reading frames (ORFs) were identified. In the case that multiple potential ORFs shared a stop codon, the longest ORF within the expected range of LanA lengths was used. These ORFs were discarded if they were too long, too short, occurred entirely within an annotated gene, or did not contain a Cys residue. The remaining ORFs were then analyzed using FIMO [1] to identify conserved leader motifs. If the ORF contained a leader motif and has a GG, GA, or S/T (x)$_{2\text{-}7}$C motif downstream of the leader, but not within 10 residues of the end of the ORF, the approximate core was identified as starting immediately after the GG or GA motif or 1 reside before the S/T (x)$_{2\text{-}7}$C motif. If the ORF contained multiple GG or GA motifs, only the first one was considered, likewise with the S/T (x)$_{2\text{-}7}$C motif. If the ORF contained both a GG or GA motif and a S/T (x)$_{2\text{-}7}$C motif, the longer potential core was used. If the ORF did not contain a GG, GA, or S/T (x)$_{2\text{-}7}$C motif, or those motifs were within 10 residues of the end of the ORF, the C-terminal half of the peptide was identified as the potential core. If no leader peptide motif was identified in the ORF, the same analysis was performed starting from the beginning of the ORF instead of after the leader motif. Finally, if the predicted core did not contain a Cys residue, the ORF was discarded. The given features were then calculated for each potential ORF.

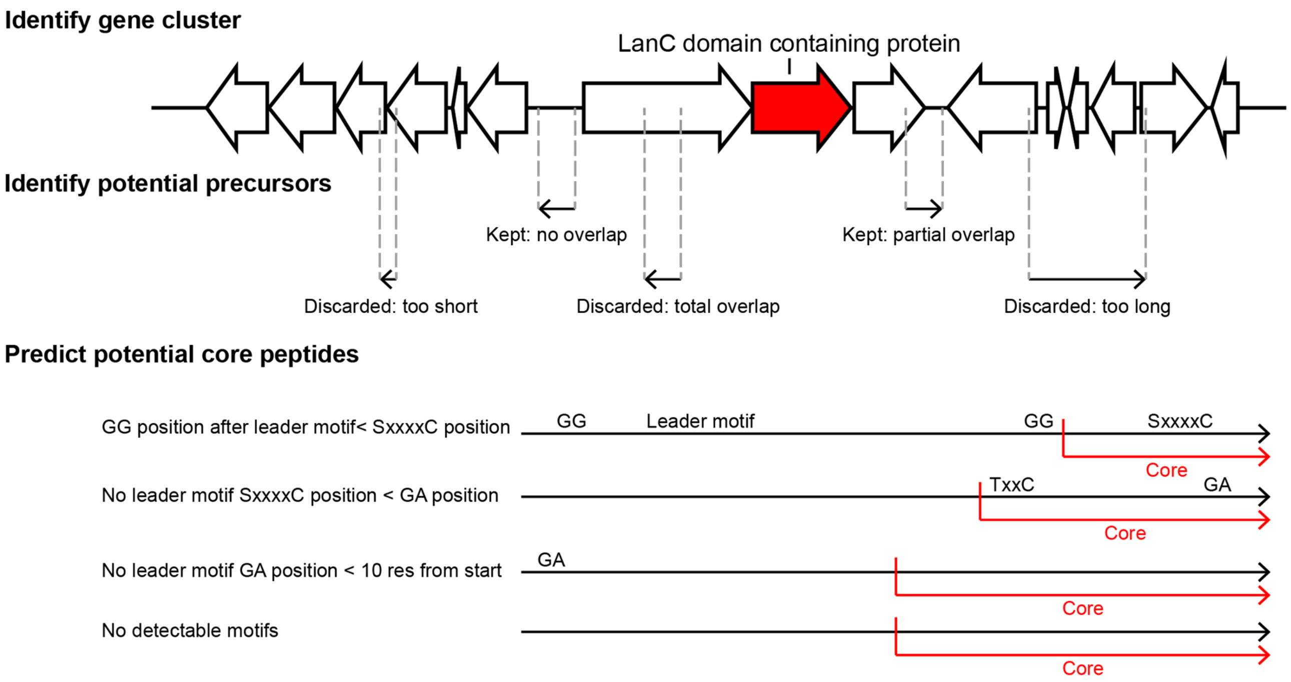

Score potential core peptides

Molecular weight of core
Isoelectric point of core
Number of each amino acid in core
Number of Ser + Thr in core
Number of Ser + Thr + Cys in core
Number of Charged residues in core
Number of positive charges in core
Number of negative charges in core
Net charge of core
Number of polar residues in core
Number of aliphatic residues in core
Number of aromatic residues in core
Core fraction that is Cys
Core fraction that is Ser + Thr
Core fraction that is Ser + Thr + Cys
Core fraction that is charged residues
Core fraction that is positive residues
Core fraction that is negative residues
Core fraction that is polar residues
Core fraction that is aliphatic residues
First fifth of precursor fraction S+T
First fifth of precursor fraction S+T+C
First fifth of precursor fraction C
Second fifth of precursor fraction S+T
Second fifth of precursor fraction S+T+C
Second fifth of precursor fraction C
Third fifth of precursor fraction S+T
Third fifth of precursor fraction S+T+C
Third fifth of precursor fraction C
Fourth fifth of precursor fraction S+T
Fourth fifth of precursor fraction S+T+C
Fourth fifth of precursor fraction C
Fifth fifth of precursor fraction S+T
Fifth fifth of precursor fraction S+T+C
Fifth fifth of precursor fraction C

Number of amino acid pairs

```
AA, AC, AD, ..., AW, AY  AxA, AxC, AxD, ..., AxW, AxY ... AxxxxxxA, AxxxxxxC, AxxxxxxD, ..., AxxxxxxW, AxxxxxxY
CA, CC, CD, ..., CW, CY  CxA, CxC, CxD, ..., CxW, CxY ... CxxxxxxA, CxxxxxxC, CxxxxxxD, ..., CxxxxxxW, CxxxxxxY
:                        :                                :
:                        :                                :
WA, WC, WD, ..., WW, WY  WxA, WxC, WxD, ..., WxW, WxY ... WxxxxxxA, WxxxxxxC, WxxxxxxD, ..., WxxxxxxW, WxxxxxxY
YA, YC, YD, ..., YW, YY  YxA, YxC, YxD, ..., YxW, YxY ... YxxxxxxA, YxxxxxxC, YxxxxxxD, ..., YxxxxxxW, YxxxxxxY
```

Supplementary Table S1. Features and scoring for class I precursors. ORFs were identified as precursors if their score was over 10. SVM, support vector machine.

Feature	Score
SVM classification	5
Presence of Class I leader peptide MEME motif	5
Core pI less than 9	2
2 or more Cys in core	2
Leader has KLxLxK MEME motif and ends in GG sequence	1

Supplementary Table S2. Features and scoring for class II precursors. ORFs were identified as precursors if their score was over 10.

Feature	score
SVM classification	5
Presence of Class II leader peptide MEME motif	5
2 or more Cys in core	2
Hits a Pfam* hidden Markov model	5
Precursor ends with sequence KRC	4

*PF08130.1, PF04604.12, PF14867.5, PF16934.4, PF02979.15, PF07862.10

Supplementary Table S3. Features and scoring for class III precursors. ORFs were identified as precursors if their score was over 10.

Feature	score
SVM classification	5
Presence of Class III leader peptide MEME motif	5
2 or more Cys in core	2
Has SxxSxxxC motif	1
Has SxxSxxC motif	1
Core pI is between 3 and 9	1

Supplementary Table S4. Features and scoring for class IV precursors. ORFs were identified as precursors if their score was over 10.

Feature	score
SVM classification	5
Presence of Class IV leader peptide MEME motif	5
2 or more Cys in core	2
Precursor ends with sequence GCD	1
Precursor ends with sequence LGS	1

Supplementary Figure S2. Sequence motifs present in more than 100 lanthipeptide precursor peptides. Some of the conserved leader peptide motifs have been shown to be important for the cognate lanthipeptide synthases (FxLD in class I [2]; E/D(-8)L/M(-9) in class II [3]; LxLQ in class III [4], and Lx_2LPE in class IV [5]). Lipid II-binding motifs, which arise for likely antibiotic lanthipeptides are underlined in red.

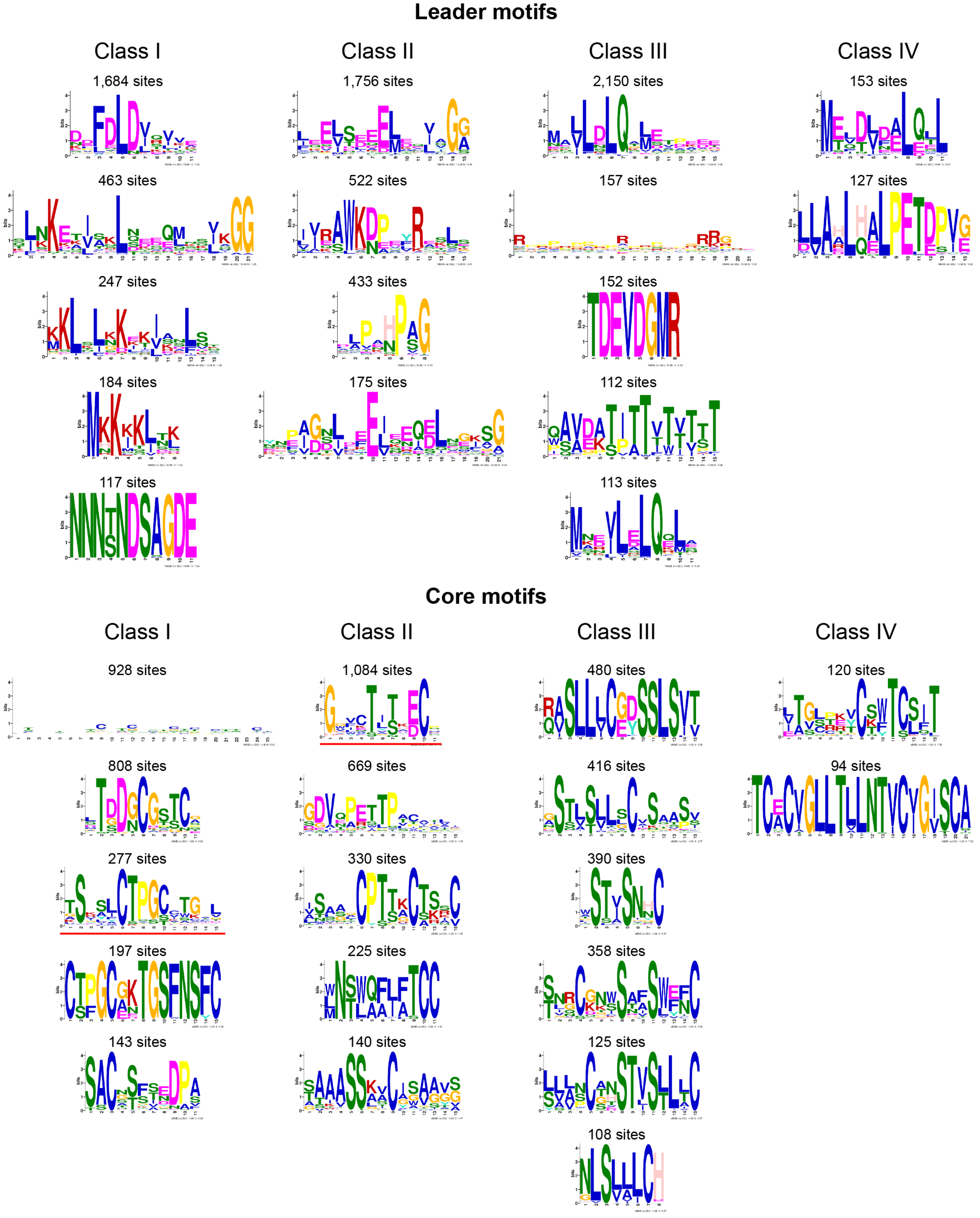

Supplementary Figure S3. Sequence similarity network of predicted precursor peptides generated with the Enzyme Function Initiative-Enzyme Similarity Tool [6] with permissive similarity cutoff (alignment score: 6; equivalent to an expectation value of 10^{-6}) and visualized in Cytoscape [7].

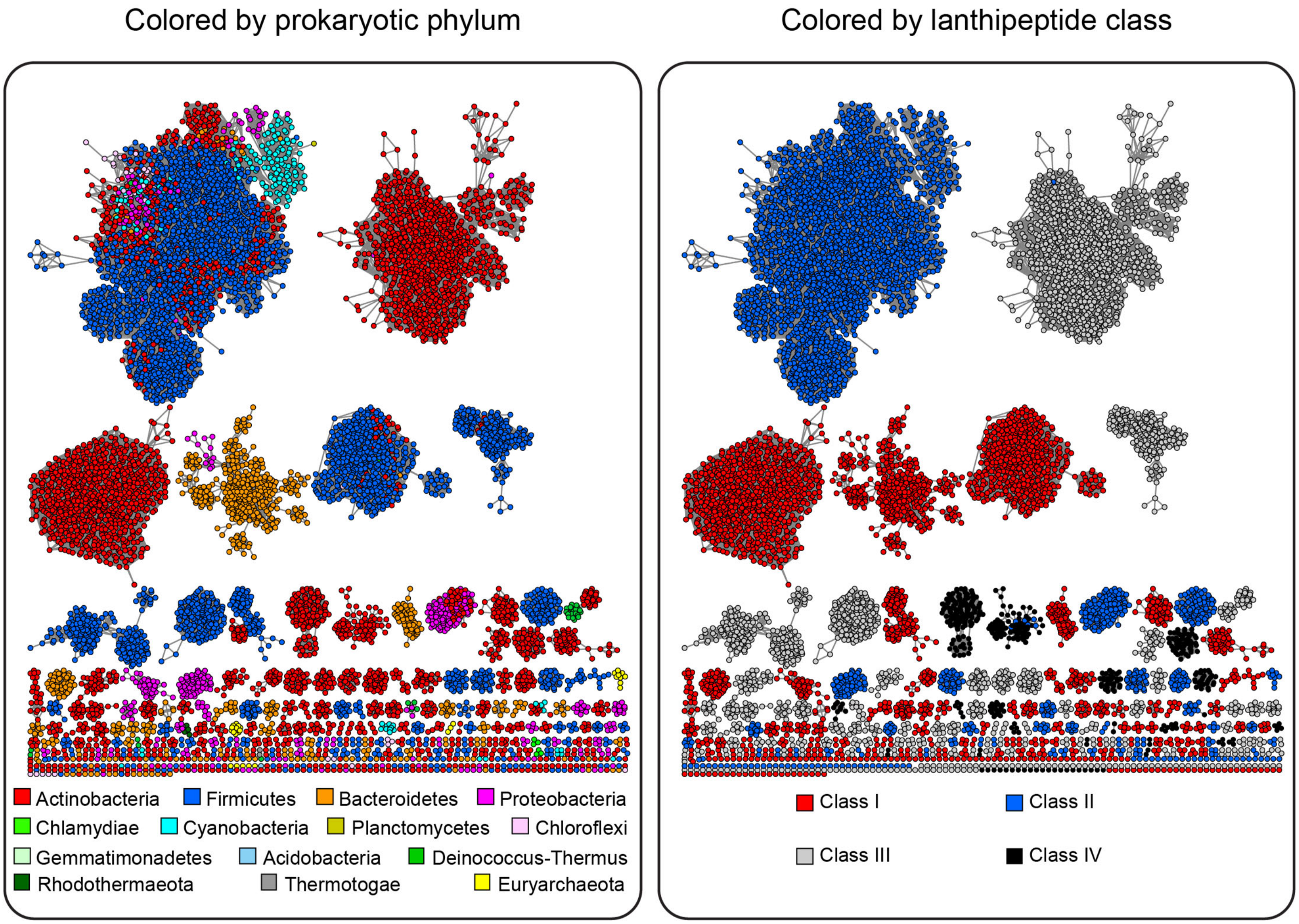

Supplementary Table S5. Location of top BLAST hits of known lanthipeptides from the MIBiG [8] and BAGEL [9] databases in the sequence similarity networks presented in Figure 2. If the precursor is located in a cluster with more than 20 members, the identifier of that cluster is given. If the precursor is located in a cluster with 20 or fewer members, the label from Figure 2 is given. If the precursor occurred only once, it is annotated as a singleton.

Known Lanthipeptide	Cluster
AmfS	III 1
BacCH91	I 5
BhtA1	BhtA1
BhtA2	BhtA2
Bicereucin_beta	II 33
BLD_1648	BLD_1648
Bovicin_HJ50	Bovicin HJ50
BsaA2	I 5
Carnolysin_A1	II 16
Carnolysin_A2	II 16
Catenulipeptide	singleton
Cerecidin	II 3
Cinnamycin_B	Cinnamycin B
CylL$_L$	II 46
CylL$_S$	II 3
Curvopeptin	singleton
Divamide*	Cinnamycin B
Duramycin*	Cinnamycin B
Entianin	I 7
Epidermin	I 5
Ericin_A	I 7
Ericin_S	I 7
Erythreapeptin	III 4
Flavecin_A1	Flavecin A1
Flavucin	Flavucin
Gallidermin	I 5
Geobacillin_I	I 7
Geobacillin_II	Geobacillin II
Griseopeptin	III 1
Haloduracin_alpha	II 4
Haloduracin_beta	Haloduracin_beta
Informatipeptin	III 2
Lacticin 3147 α*	II 9
Lacticin 3147 β*	II 6
Lacticin 481*	II 1
Lichenicidin_VK21_A1	II 4
Lichenicidin_VK21_A2	II 7
Macedocin	II 1
Macedovicin	Bovicin HJ50
McdA1	II 1
Mersacidin	Mersacidin
Michiganin A	Michiganin A
Microbisporicin	Microbisporicin
Mutacin 1140*	I 5
Mutacin II	II 1
Mutacin K8	II 1
Nisin_A	I 7
Nisin_O	I 7
Nisin_U	I 7
Nisin_Z	I 7
Nukacin_ISK-1	II 1
Paenibacillin	Paenibacillin
Paenicidin_A	Paenicidin A
Paenicidin_B	Paenicidin A
Paenilan	I 20
Penisin	I 23
Pinensin	I 17
Plantaricin C	Plantaricin C
Pneumolancidin_PldA2	II 11
Pneumolancidin_PldA3	II 11
Pneumolancidin_PldA4	II 11
Prochlorosin_1.1	Prochlorosin 1.1
Pseudomycoicidin	Geobacillin II
Roseocin α	II 29
Roseocin β	II 2
Ruminococcin_A	II 1
SAL-2242	III 1
Salivaricin_9	II 1
Salivaricin_A	II 14
Salivaricin_G32	II 1
Sap_T	I 21
SapB	III1
Smb_A	BhtA2
Smb_B	BhtA1
SRO15-2212	III 1
SRO15-3108	II 2
Stackepeptin_A	singleton
Stackepeptin_B	singleton
Stackepeptin_C	singleton
Stackepeptin_D	singleton
Staphylococcin_C55_alpha	II 9
Staphylococcin_C55_beta	II 6
Streptin	I 14
Streptococcin_A-FF22	II 1
StreptococcinA-M49	II 1
Subtilin	I 7
Subtilomycin	Subtilomycin
Suicin_3908	Bovicin HJ50
Suicin_65	II 1
Suicin_90-1330	I 7
Thermophilin_1277	Bovicin HJ50
Thusin_A	II 4
Thusin_B	Thusin B

*top blast hit is not identical, possibly because the genome of the producer is not in the database.

Supplementary Figure S4. Sequence logos generated from alignments of class I precursor peptides in clusters with 20 or more members (in Figure 2) using WebLogo [10]. Conserved leader motifs that are shared among multiple clusters (from Figure S2) are boxed in gray and potential lipid II-binding motifs are overlined in red. Families with no previously characterized members are highlighted in yellow.

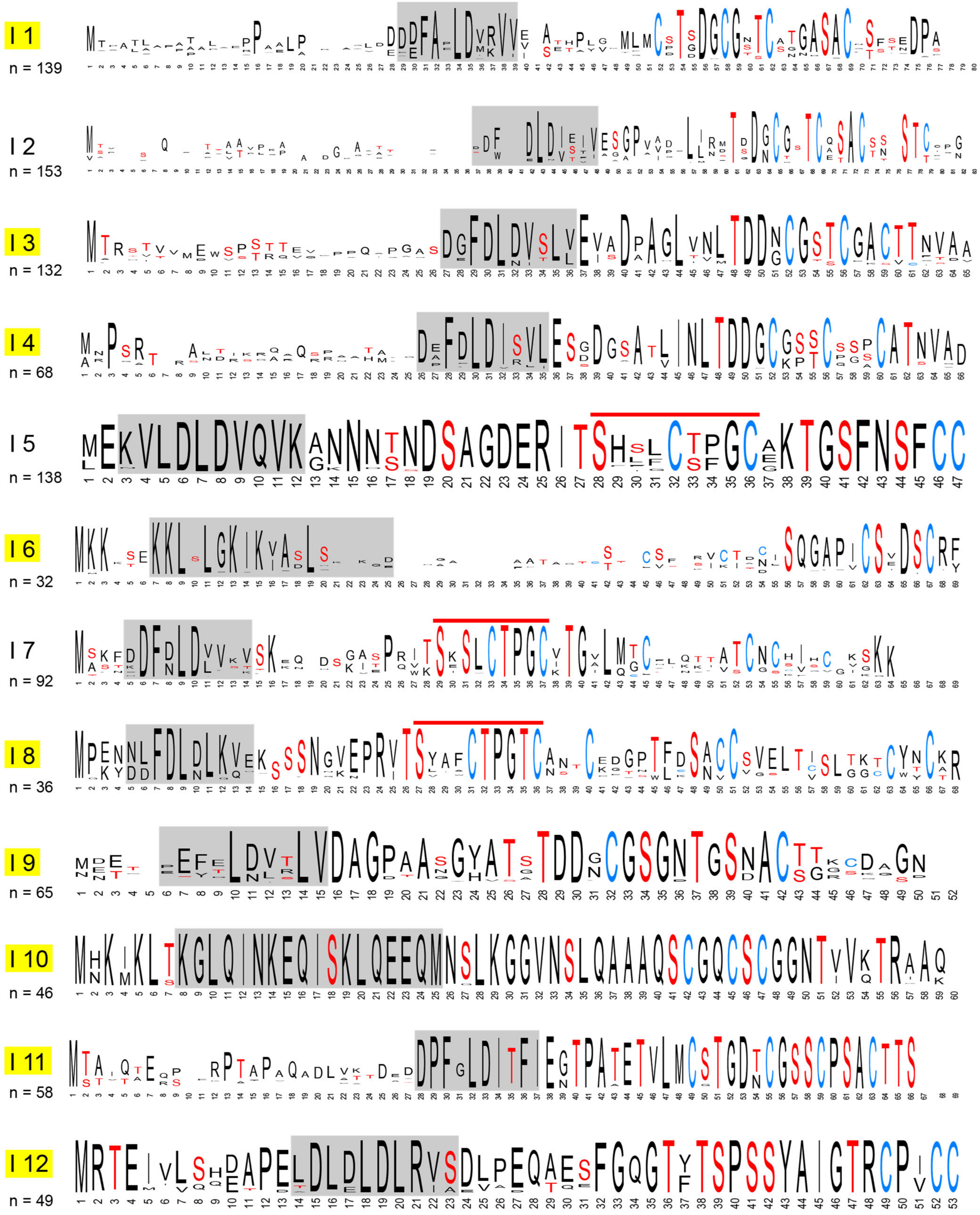

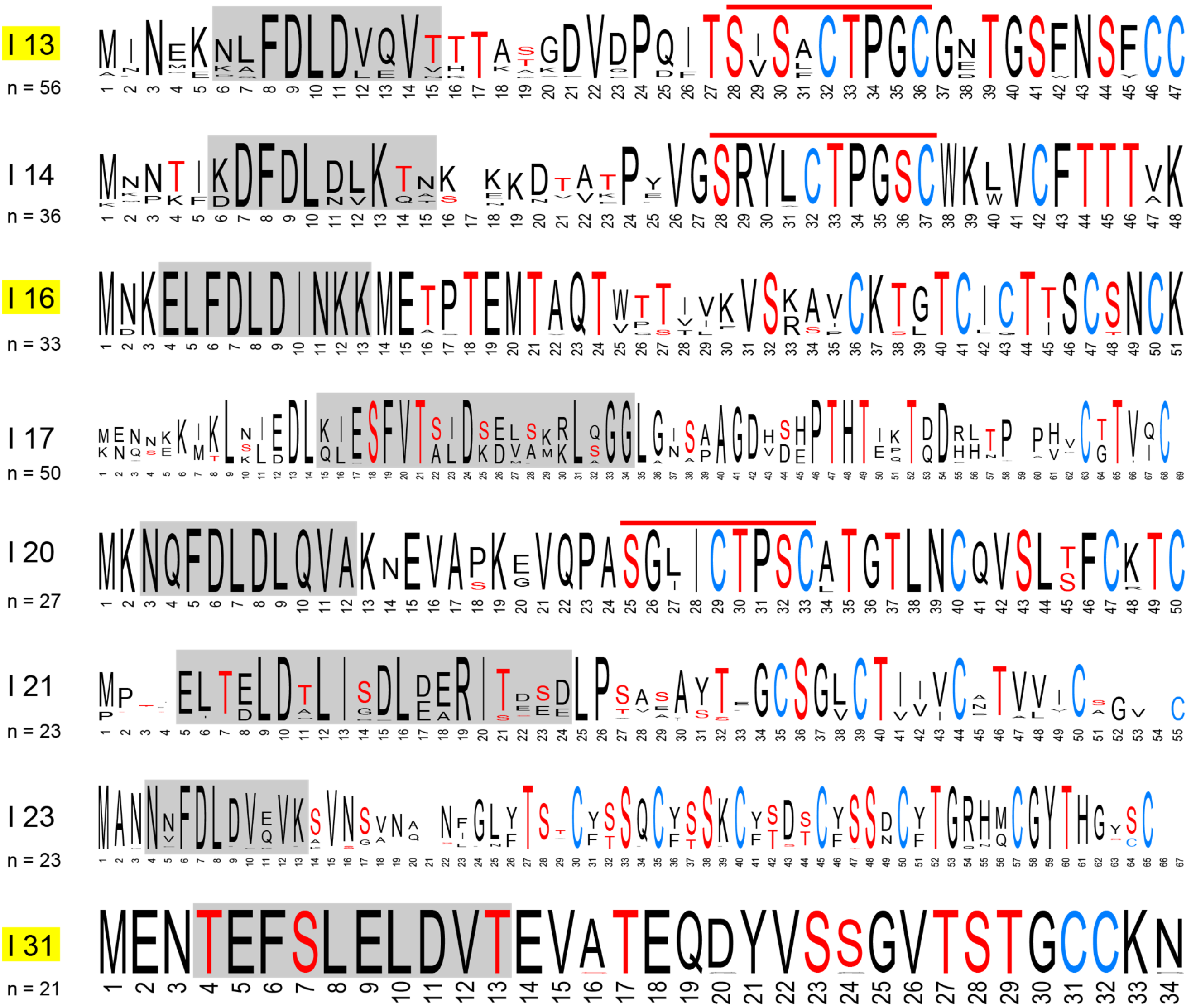
I 13
n = 56
I 14
n = 36
I 16
n = 33
I 17
n = 50
I 20
n = 27
I 21
n = 23
I 23
n = 23
I 31
n = 21

Supplementary Figure S5. Sequence logos generated from alignments of class II precursor peptides in clusters with 20 or more members (in Figure 2) using WebLogo [10]. Conserved leader motifs that are shared among multiple clusters (from Figure S2) are boxed in gray and potential lipid II-binding motifs are overlined in red. Families with no previously characterized members are highlighted in yellow.

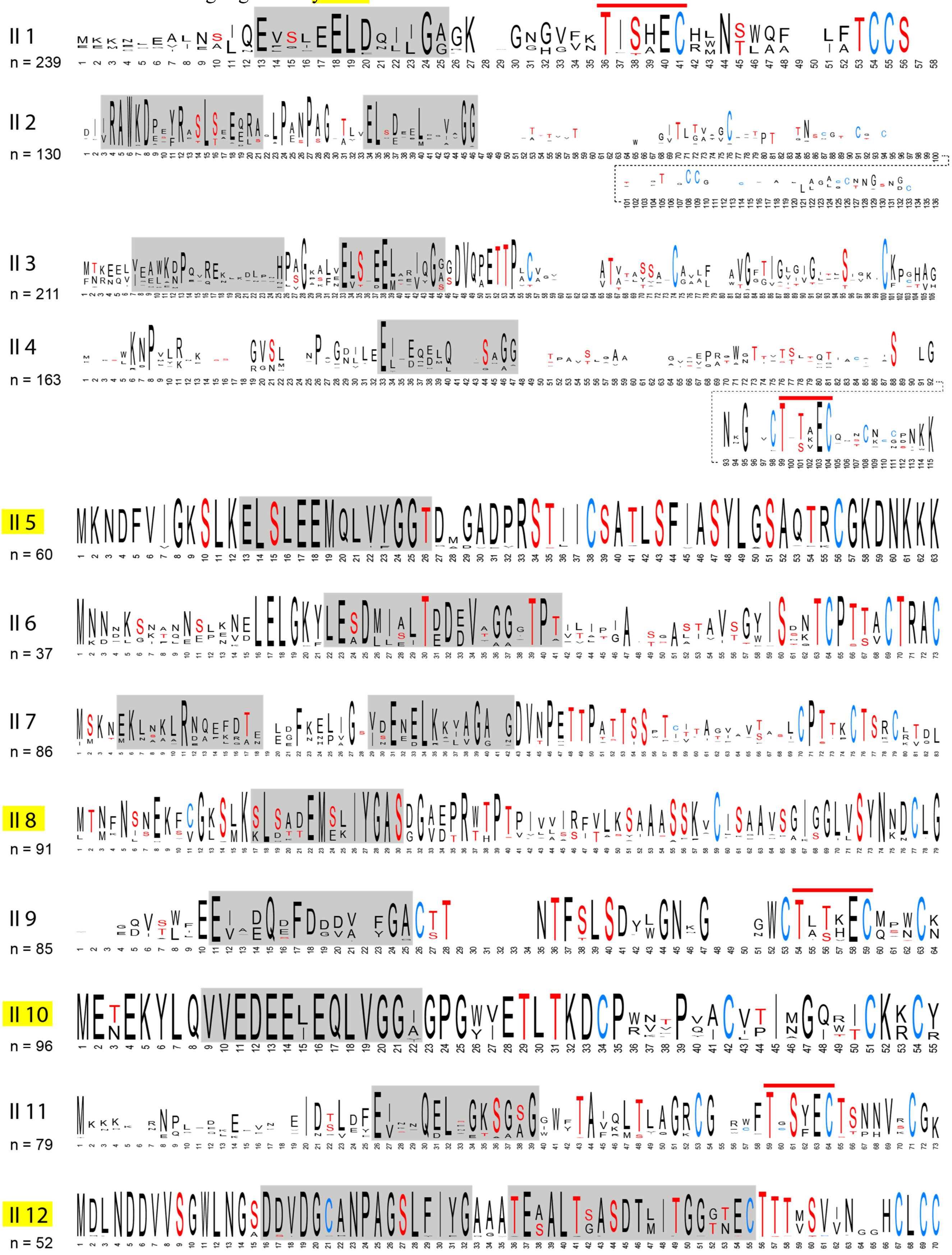

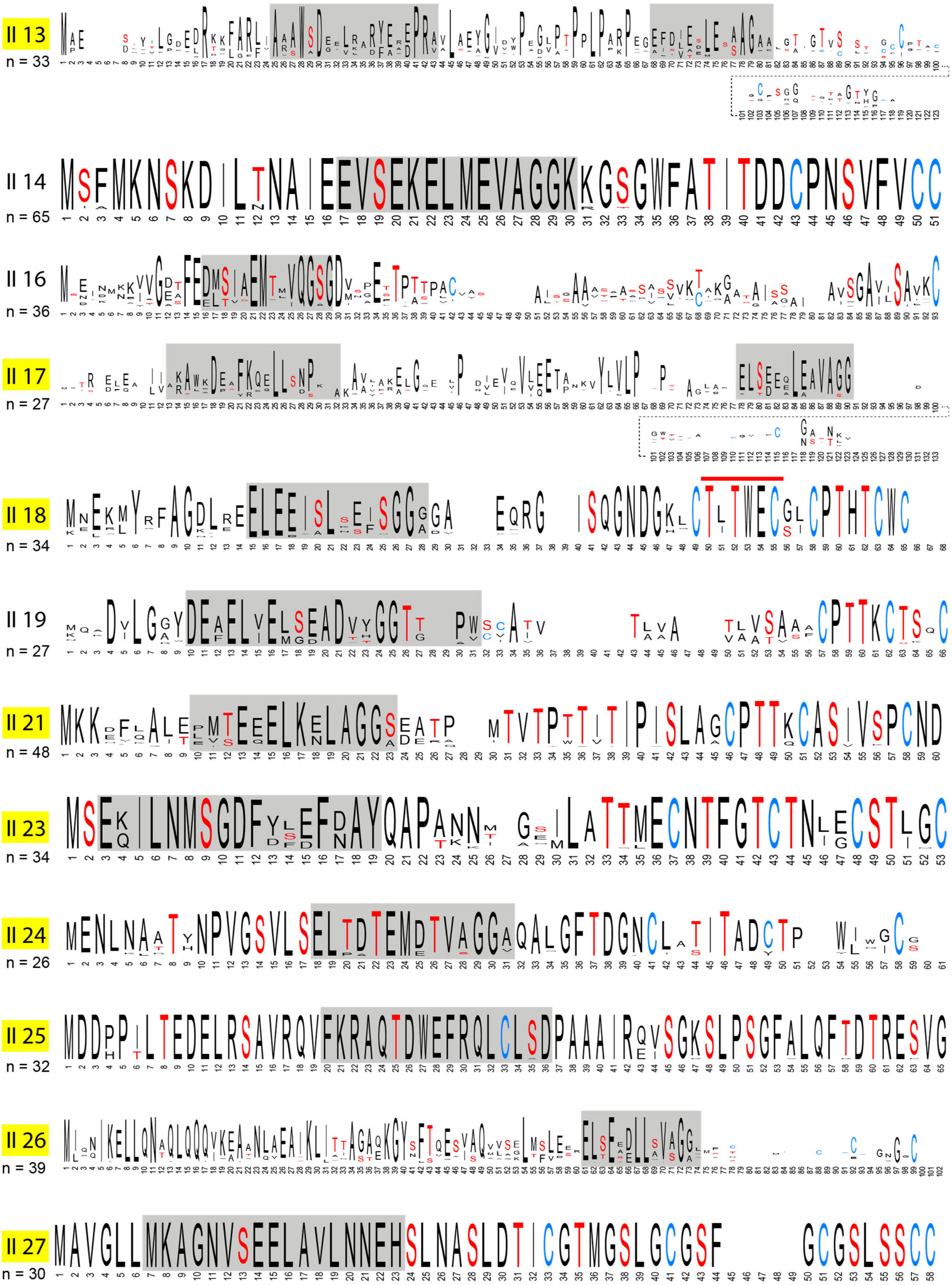
II 13
n = 33
II 14
n = 65
II 16
n = 36
II 17
n = 27
II 18
n = 34
II 19
n = 27
II 21
n = 48
II 23
n = 34
II 24
n = 26
II 25
n = 32
II 26
n = 39
II 27
n = 30

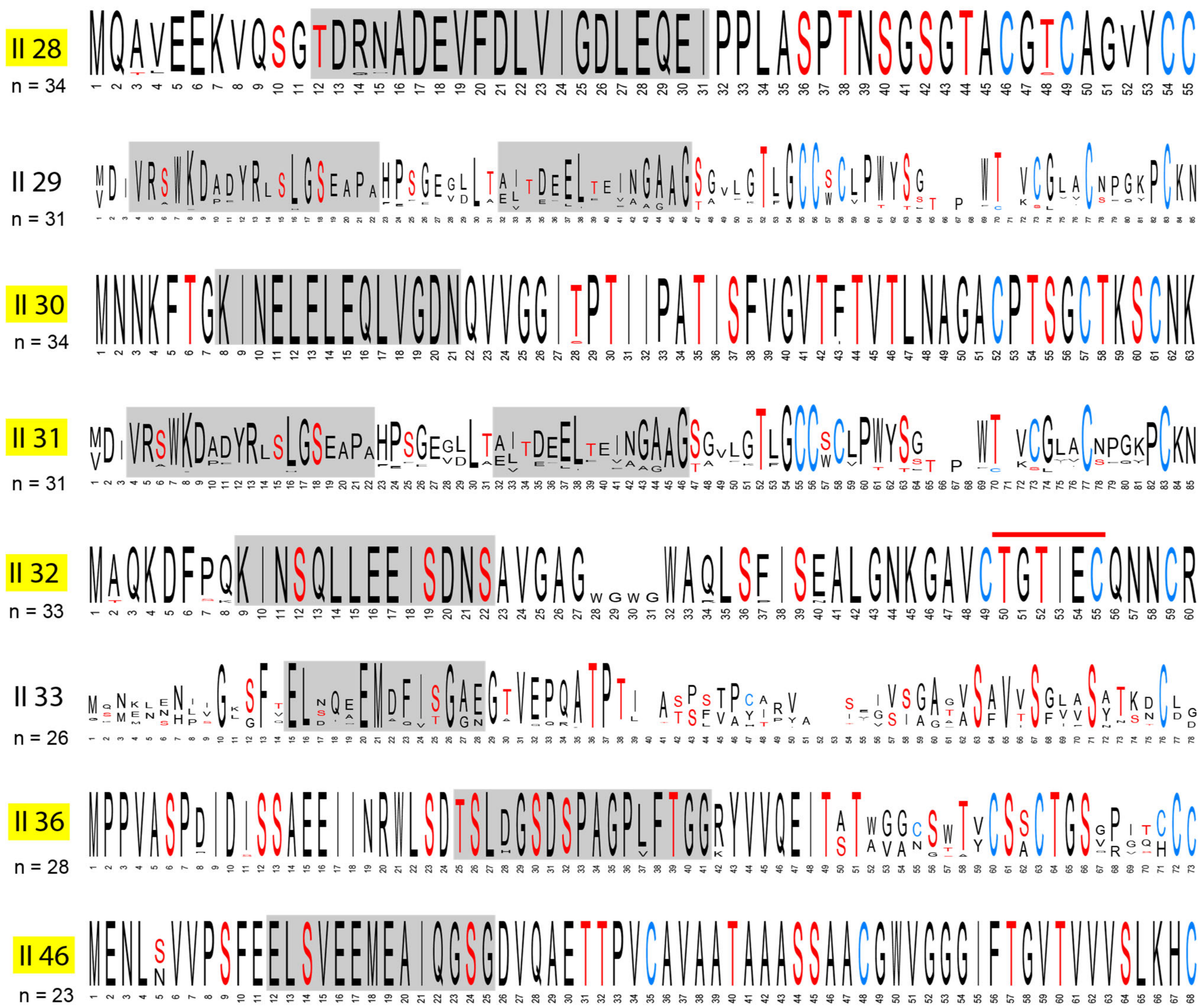
II 28
n = 34
II 29
n = 31
II 30
n = 34
II 31
n = 31
II 32
n = 33
II 33
n = 26
II 36
n = 28
II 46
n = 23

Supplementary Figure S6. Sequence logos generated from alignments of class III precursor peptides in clusters with 20 or more members (in Figure 2) using WebLogo [10]. Conserved leader motifs that are shared among multiple clusters (from Figure S2) are boxed in gray. Families with no characterized members are highlighted in yellow.

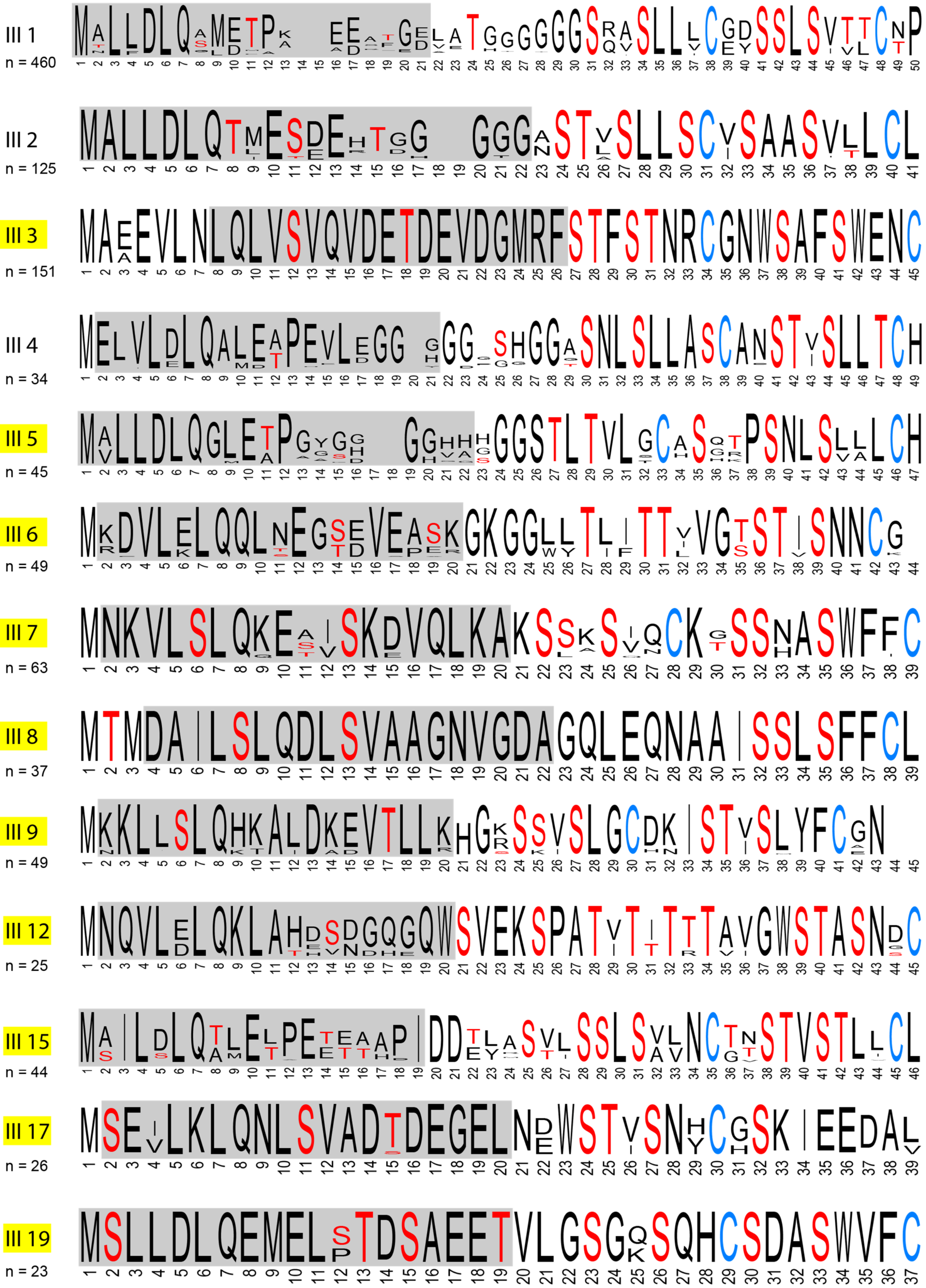

Supplementary Figure S7. Sequence logos generated from alignments of class IV precursor peptides in clusters with 20 or more members (in Figure 2) using WebLogo [10]. Conserved leader motifs that are shared among multiple clusters (from Figure S2) are boxed in gray. Families with no previously characterized members are highlighted in yellow.

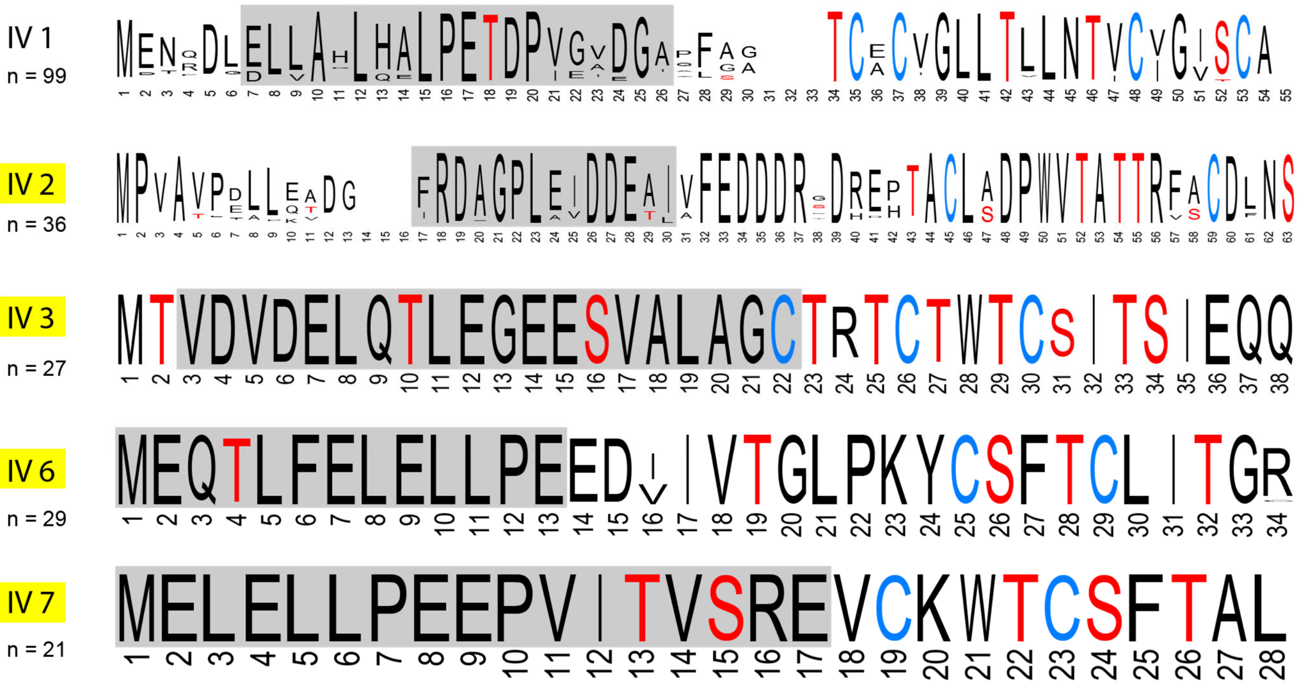

Supplementary Table S6. Twenty most abundant proteins in class I BGCs that belong to at least one Pfam family. If a protein has multiple domains from different Pfam families, those families are separated by a slash. Potential secondary modification enzymes are highlighted in yellow. Split LanBs, with the glutamylation and elimination domains on separate polypeptides, are denoted sLanB.

Pfam families	Description	Count
LANC_like (PF05147)	LanC	2,204
Lant_dehydr_N (PF04738)/Lant_dehydr_C (PF14028)	LanB	1,745
ABC_tran (PF00005)	LanT	657
Lant_dehydr_C (PF14028)	LanB elimination domain	562 (193 sLanBs)
PCMT (PF01135)	Protein-L-isoaspartate(D-aspartate) *O*-methyltransferase	571
Gallidermin (PF02052)	Gallidermin- and nisin-like precursor peptides	426
ABC2_membrane_4 (PF12730)	LanT	363
HTH_31 (PF13560)	transcriptional regulator	307
Lant_dehydr_N (PF04738)	PEARL	304 (193 sLanBs)
ABC_membrane (PF00664)/ABC_tran (PF00005)	LanT	283
Lant_dehydr_C (PF14028)/PCMT (PF01135)	LanB elimination domain and Protein-L-isoaspartate(D-aspartate) *O*-methyltransferase fusion protein	261
Peptidase_C39 (PF03412)/ABC_membrane (PF12730)/ABC_tran (PF00005)	$LanT_P$	258
HATPase_c_2 (PF13581)	Histidine kinase-like ATPase	197
Response_reg (PF00072)/Trans_reg_C (PF00486)	transcriptional regulator	180
Peptidase_S8 (PF00082)	$LanP_A$	172
Flavoprotein (PF02441)	LanD	146
Acetyltransf_1 (PF00583)	*N*-acetyltransferase	142
DUF397 (PF04149)	Domain of Unknown Function	140
MFS_1 (PF07690)	Major Facilitator Superfamily protein	128
Leukocidin (PF07968)	Leukocidin/Hemolysin toxin family protein	127

Supplementary Table S7. Twenty most abundant proteins in class II BGCs that belong to at least one Pfam family. If a protein has multiple domains from different Pfam families, those families are separated by a slash. Potential tailoring enzymes are highlighted in yellow.

Pfam families	Description	Count
DUF4135 (PF13575)/LANC_like (PF05147)	LanM	2,163
Peptidase_C39 (PF03412)/ABC_membrane (PF12730)/ABC_tran (PF00005)	$LanT_P$	1,270
ABC_tran (PF00005)	LanT	886
ABC2_membrane_4 (PF12730)	LanT	761
Mersacidin (PF16934)	Mersacidin-like precursor peptide	705
L_biotic_typeA (PF04604)	Type A lanthipeptide precursor peptide	532
HTH_3 (PF01381)	transcriptional regulator	348
Lantibiotic_a (PF14867)	Alpha precursor peptide	339
Response_reg (PF00072)/GerE (PF00196)	transcriptional regulator	308
Peptidase_S8 (PF00082)	$LanP_A$	291
ABC_membrane (PF00664)/ABC_tran (PF00005)	LanT	222
ABC_tran (PF00005)/DUF4162 (PF13732)	LanT	213
FMN_red (PF03358)	Flavin mononucleotide reductase	186
HisKA (PF00512)/HATPase_c (PF02518)	2-component response regulator	180
Response_reg (PF00072)/Trans_reg_C (PF00486)	transcriptional regulator	164
GerE (PF00196)	transcriptional regulator	140
Nhase_alpha (PF02979)	precursor peptide with nitrile hydratase family leader peptide	119
Nif11 (PF07862)	precursor peptide with Nif11 family leader peptide	109
FtsX (PF02687)	FtsX-like permease family	104
LANC_like (PF05147)	LanC (split LanM possibly from sequencing errors)	95

Supplementary Table S8. Twenty most abundant proteins in class III BGCs that belong to at least one Pfam family. If a protein has multiple domains from different Pfam families, those families are separated by a back slash. Potential tailoring enzymes are highlighted in yellow. LanP$_P$ is a Pro oligopeptidase that is distinct from the LanP$_A$ subtilin-like S8 peptidases.

Pfam families	Description	Count
ABC_membrane (PF00664)/ABC_tran (PF00005)	LanT	1,514
Pkinase (PF00069)/LANC_like (PF05147)	LanKC	1,511
ABC_tran (PF00005)	LanT	781
Response_reg (PF00072)/GerE (PF00196)	transcriptional regulator	715
GerE (PF00196)	transcriptional regulator	263
MFS_1 (PF07690)	Major facilitator superfamily protein	243
adh_short (PF00106)	short chain dehydrogenase	156
GAF_2 (PF13185)/PAS_3 (PF08447)/GAF_2 (PF13185)/SpoIIE (PF07228)/HATPase_c_2 (PF13581)	Unknown	135
trypsin (PF00089)	Protease	133
Pkinase (PF00069)	protein kinase	133
HTH_20 (PF12840)	transcriptional regulator	123
Acetyltransf_1 (PF00583)	N-acetyltransferase	122
BPD_transp_1 (PF00528)	Binding-protein-dependent transport system, inner membrane component	121
FtsX (PF02687)/FtsX (PF02687)	FtsX-like permease	119
GAF_2 (PF13185)/PAS_4 (PF08448)/GAF_2 (PF13185)/SpoIIE (PF07228)	Unknown	114
Acetyltransf_3 (PF13302)	*N*-acetyltransferase	113
Peptidase_S9 (PF00326)	LanP$_P$	112
FecCD (PF01032)	FecCD transport family	102
Mac (PF12464) /Hexapep (PF00132)	Acetyltransferase	97
DUF4265 (PF14085)	Domain of unknown function	93

Supplementary Table S9. Twenty most abundant proteins in class IV BGCs that belong to at least one Pfam family. If a protein has multiple domains from different Pfam families, those families are separated by a back slash. Known class IV BGCs comprise only 4 genes, so these entries may include proteins encoded by genes that are not part of the gene cluster. Potential tailoring enzymes are highlighted in yellow.

Pfam families	Description	Count
Pkinase (PF00069)/LANC_like (PF05147)	LanL	340
ABC_membrane (PF00664)/ABC_tran (PF00005)	LanT	164
Peptidase_S9 (PF00326)	LanP$_P$	112
MFS_1 (PF07690)	Major facilitator Superfamily protein	101
ABC2_membrane (PF01061)	LanT	83
ABC_tran (PF00005)/DUF4162 (PF13732)	LanT	82
HATPase_c_2 (PF13581)	Histidine kinase-like ATPase domain	48
ABC_tran (PF00005)	LanT	48
Nif3 (PF01784)	NGG1p interacting factor 3	46
NAD_binding_10 (PF13460)	NAD(P)H-binding protein	35
STAS_2 (PF13466)	STAS domain containing protein	31
DAO (PF01266)	FAD dependent oxidoreductase	30
BPD_transp_1 (PF00528)	Binding-protein-dependent transport system, inner membrane component	30
TrmK (PF04816)	N-methyltransferase	29
Glycos_transf_2 (PF00535)	Glycosyl transferase	28
SBP_bac_3 (PF00497)	Bacterial extracellular solute-binding protein	26
Methyltransf_19 (PF04672)	Methyltransferase	26
GDP_Man_Dehyd (PF16363)	GDP-mannose 4,6-dehydratase	26
SNF2_assoc (PF08455)/SNF2_N (PF00176)/Helicase_C (PF00271)	Helicase	24
Response_reg (PF00072)/Sigma70_r4_2 (PF08281)	Response regulator	25

Supplementary Table S10. Distribution among the lanthipeptide classes of Pfams that are in the 20 most abundant protein families in a single class. Values are presented on the basis of domains, so Pfam families that occur in multidomain proteins and single domain proteins are counted together. The Pfam are listed in order of decreasing frequency.

Pfam protein family	Top 20 most abundant in class	Class I	Class II	Class III	Class IV
PCMT (PF01135)	I	835	1	1	0
Flavoprotein (PF02441)	I	146	26	24	4
Acetyltransf_1 (PF00583)	I, III	149	47	142	12
FMN_red (PF03358)	II	34	186	7	4
adh_short (PF00106)	III	57	30	157	19
Acetyltransf_3 (PF13302)	III	57	12	113	9
Mac (PF12464)	III	0	0	97	0
TrmK (PF04816)	IV	0	0	0	29
DAO (PF01266)	IV	6	1	9	30
NAD_binding_10 (PF13460)	IV	21	10	19	38
GDP_Man_Dehyd (PF16363)	IV	2	0	65	26
Glycos_transf_2 (PF00535)	IV	36	8	39	29
Methyltransf_19 (PF04672)	IV	64	12	9	26

Supplementary Figure S8. Example putative biosynthetic gene clusters encoding the enzymes in Table S10. LanA genes that were not annotated in the genome are indicated in red. Because BGC boundaries are not known, the genes encoding the noted enzymes may or may not be part of the lanthipeptide BGC for all panels of Supplementary Figure S8.

O-methyltransferase containing clusters

Class I

Streptomyces clavuligerus ATCC 27064

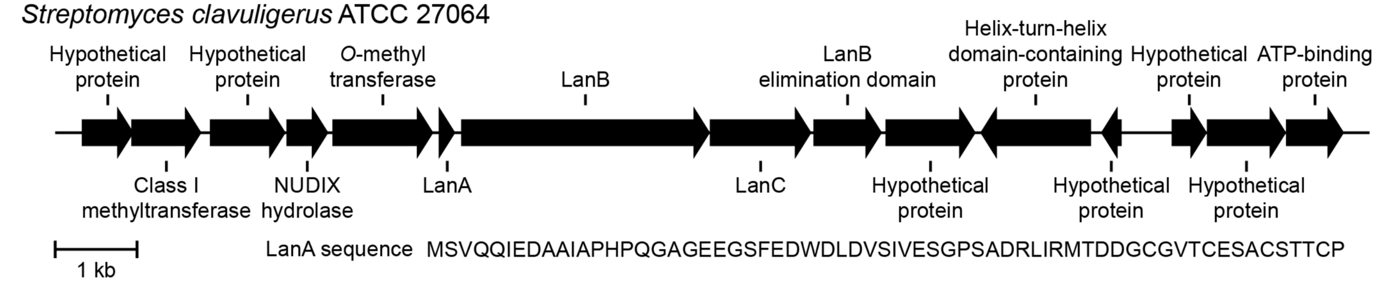

Class II

Marinicella sediminis strain F2

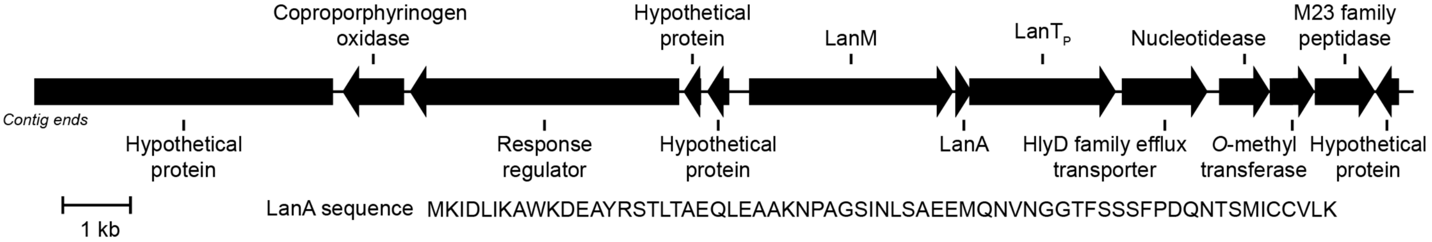

Class III

Streptomyces varsoviensis strain NRRL ISP-5346

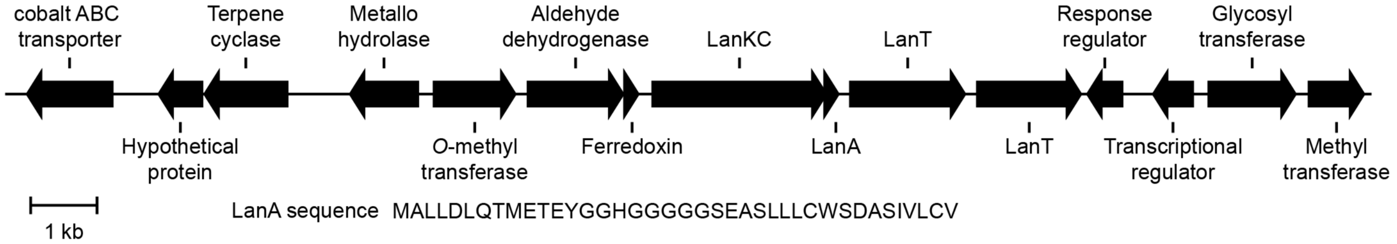

Supplementary Figure S8 continued.

Flavoprotein containing clusters

Class I

Brevibacillus sp. BC25

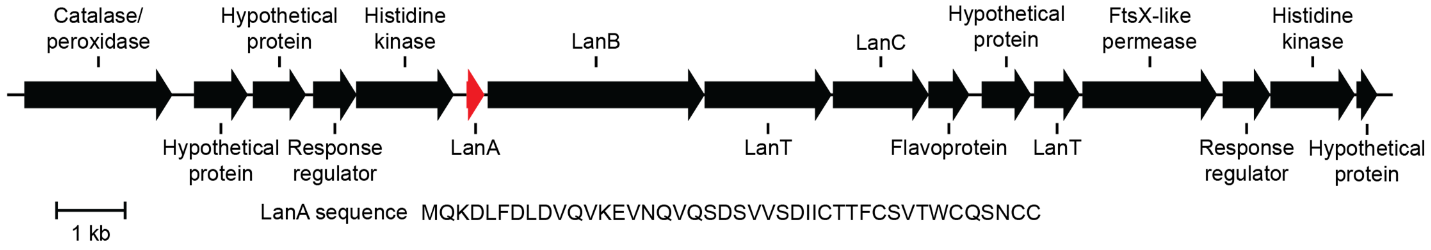

LanA sequence MQKDLFDLDVQVKEVNQVQSDSVVSDIICTTFCSVTWCQSNCC

Class II

Bacillus velezensis strain GH1-13

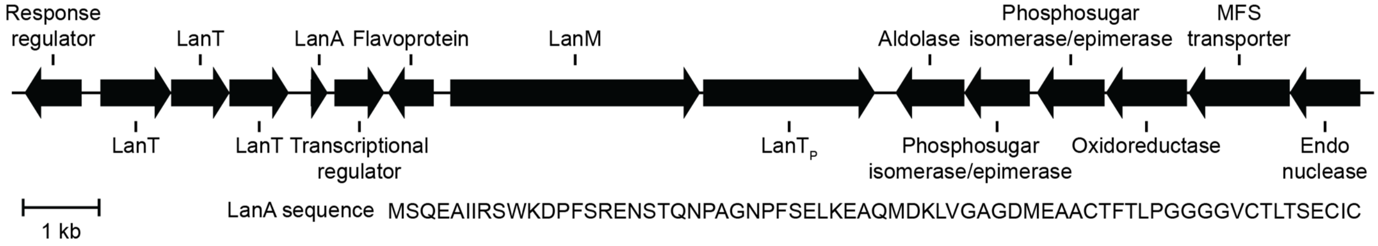

LanA sequence MSQEAIIRSWKDPFSRENSTQNPAGNPFSELKEAQMDKLVGAGDMEAACTFTLPGGGGVCTLTSECIC

Class III

Streptomyces bicolor strain NRRL B-5348

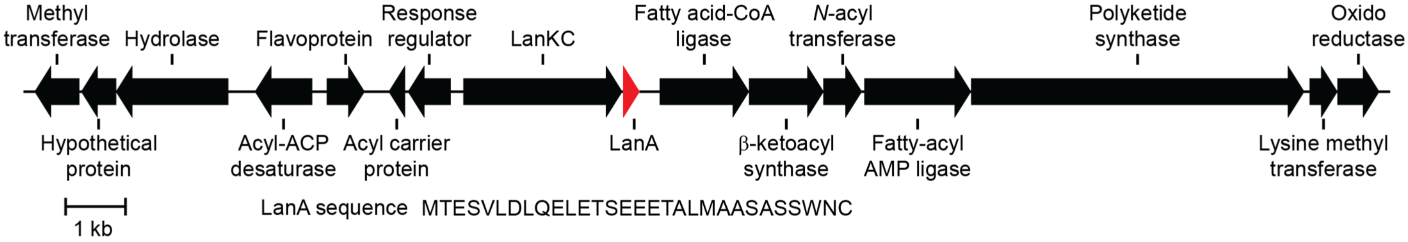

LanA sequence MTESVLDLQELETSEEETALMAASASSWNC

Class IV

Streptomyces sp. AcH 505

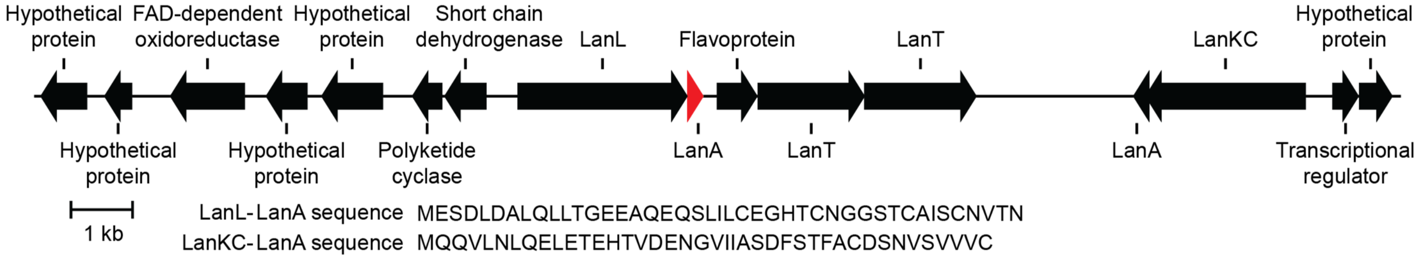

LanL-LanA sequence MESDLDALQLLTGEEAQEQSLILCEGHTCNGGSTCAISCNVTN

LanKC-LanA sequence MQQVLNLQELETEHTVDENGVIIASDFSTFACDSNVSVVVC

Supplementary Figure S8 continued.

Acyltransferase (Acyltransf_1) containing clusters

Class I

Paenibacillus polymyxa strain NCTC10343

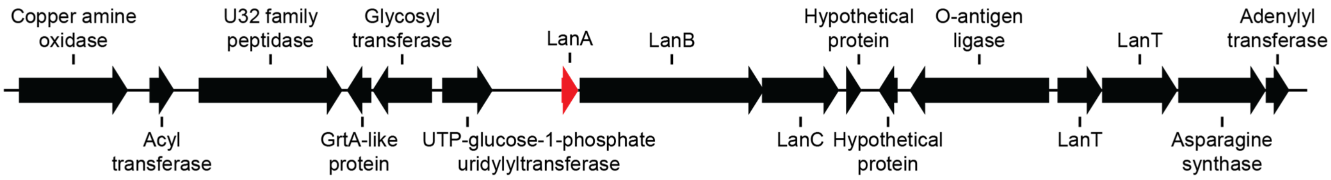

1 kb

LanA sequence MNSPELVFFEQEDTLDLDLQINDLTLKQAKNPCTSTVTCSVSRCLGTHVTCECWC

Class II

Thermoanaerobaculum aquaticum strain MP-01

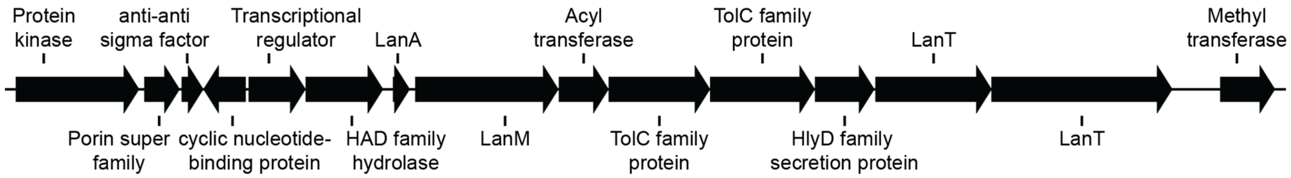

1 kb

LanA sequence MPEAVTLTRGEVNDLIAGFATKNPEYRKALLAKPREVVGAQLGTSIPPSVQVKVIEEKPNEFYVIIPHVA KEGQELSDADLEQVAGGKKDTYKCEVQGLGFGTRVEINASLF

Class III

Streptomyces sp. CNS654

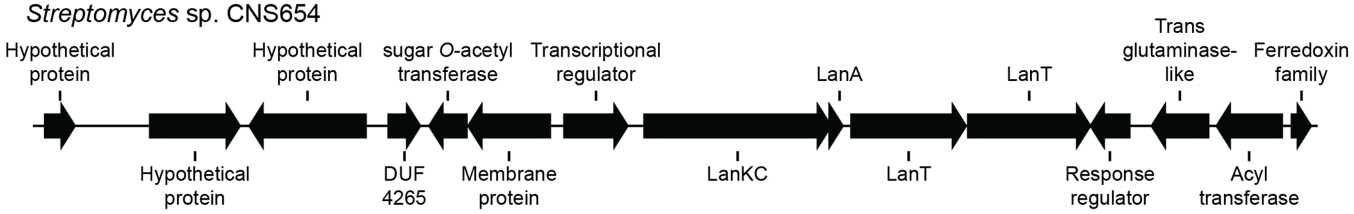

1 kb

LanA sequence MALLDLQAMDTPAEDSFGELATGSQVSLLVCEYSSLSVVLCTP

Class IV

Streptomyces baarnensis strain NRRL B-2842

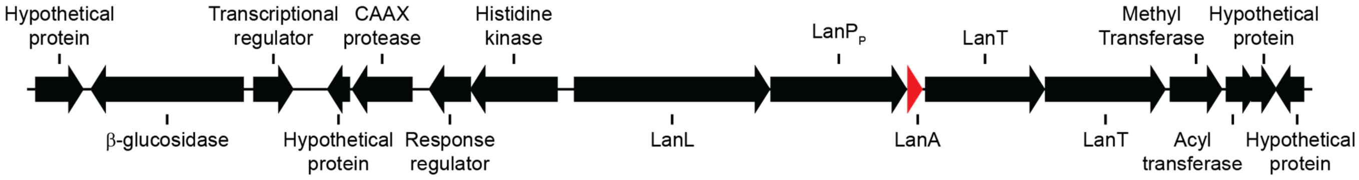

1 kb

LanA sequence MIDNTMTVDVDELQTLEGEESVALAGCTRTCTWTCSITSIEQQ

Supplementary Figure S8 continued.

FMN reductase (FMN_red) containing clusters

Class I

Listeria monocytogenes strain AL-OM-1-WH2

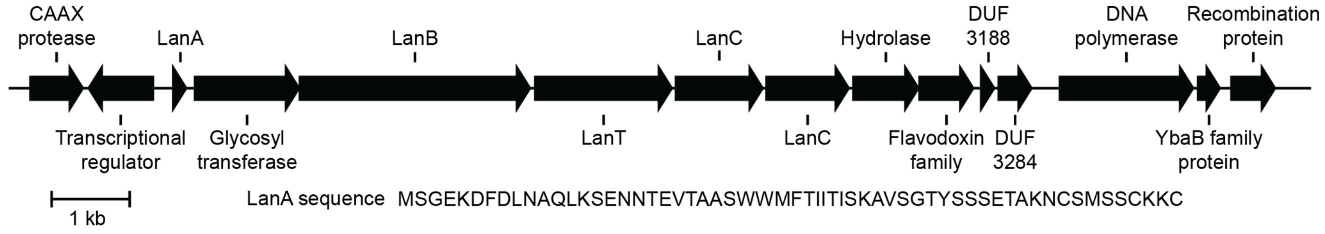

LanA sequence MSGEKDFDLNAQLKSENNTEVTAASWWMFTIITISKAVSGTYSSSETAKNCSMSSCKKC

Class II

Bacillus cereus VD166

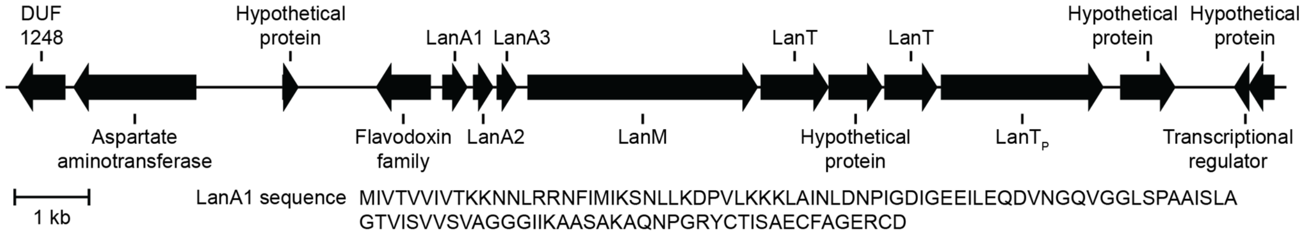

LanA1 sequence MIVTVVIVTKKNNLRRNFIMIKSNLLKDPVLKKKLAINLDNPIGDIGEEILEQDVNGQVGGLSPAAISLA GTVISVVSVAGGGIIKAASAKAQNPGRYCTISAECFAGERCD

LanA2 sequence MFSQKSYLLKNPVAKNKFGASMTNPSGDIITEIQEQELEAVNGGITPTVGWSAVAVSTLTVNSVLVVSVS ATNPGRVCTISAECSSGYKRCD

LanA3 sequence MHMNHKLMKNPVAKNKLKVLDESPAGDLLEEIQEQELQSNGYGGISPSVVATISVVSAVSIAGTSVVSAS ATNPGRYCTWSAECSWSQKRCD

Class III

Streptomyces sp. CNS654

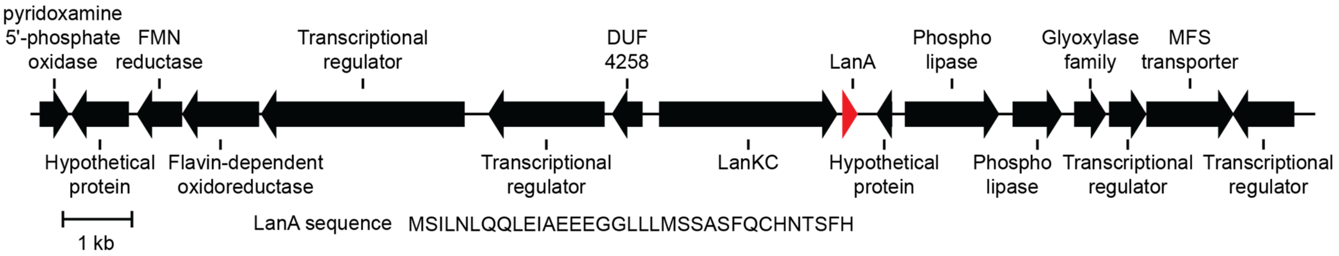

LanA sequence MSILNLQQLEIAEEEGGLLLMSSASFQCHNTSFH

Class IV

Streptomyces misionensis strain DSM 40306

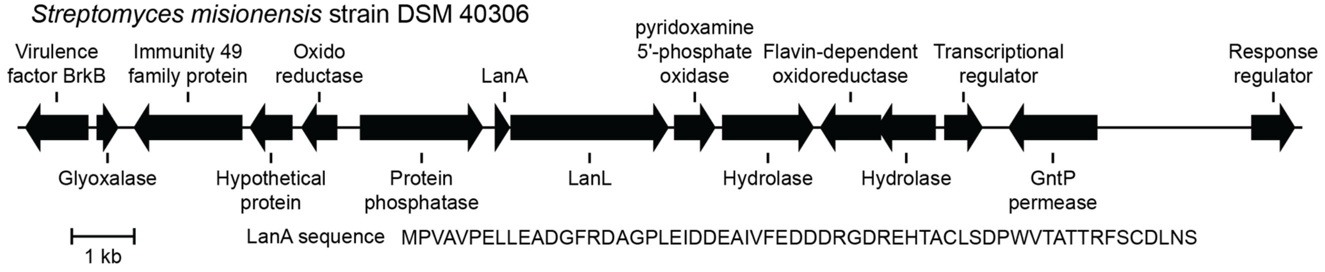

LanA sequence MPVAVPELLEADGFRDAGPLEIDDEAIVFEDDDRGDREHTACLSDPWVTATTRFSCDLNS

Supplementary Figure S8 continued.

Short chain dehydrogenase (adh_short) containing clusters

Class I

Frankia sp. QA3

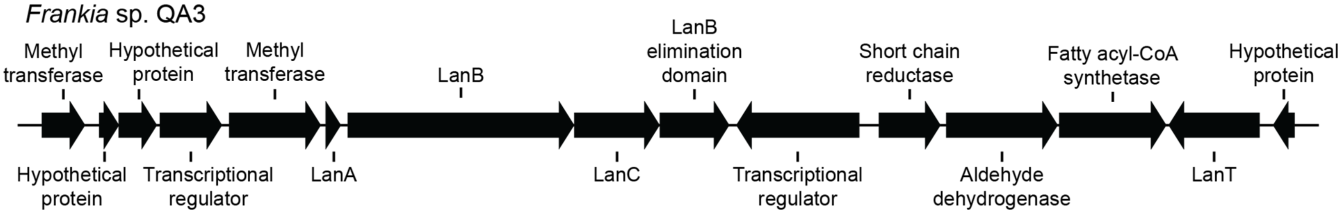

1 kb

LanA sequence MTPSTAVLAPAPVSSEPDPFDVDLDLRVIEAAGPLVITMCSTDDNCGSTCKPSACASNSADPF

Class II

Bacillus thuringiensis MC28

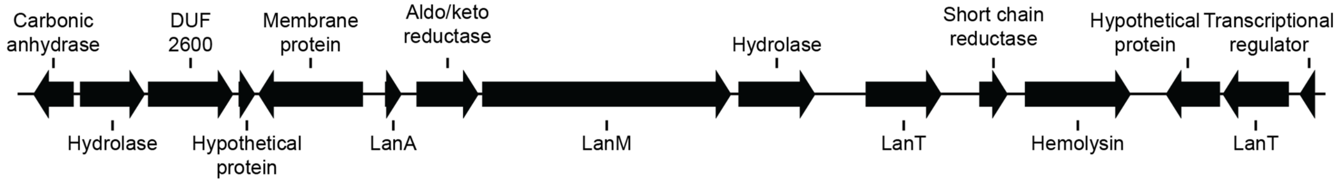

1 kb

LanA sequence MTNEQVISAWKNPDIRSIMGIFSDNPAGISFNELSTNEMEEVHGKIDPKLESYSLSCFISERFPSWCP

Class III

Rhodococcus erythropolis SK121

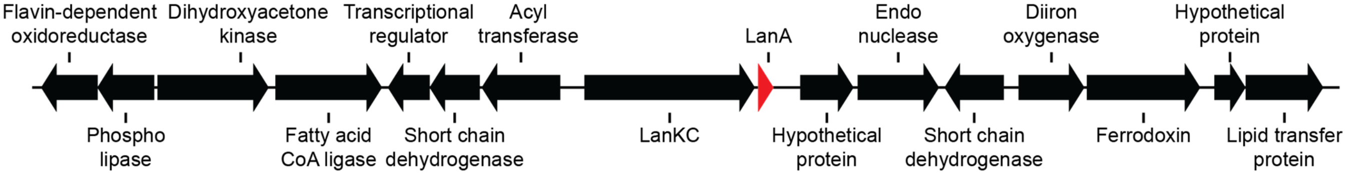

1 kb

LanA sequence MTMDAILSLQDLSVAAGNVGDAGQLEQNAAISSLSFFCL

Class IV

Streptomyces flavochromogenes strain NRRL B-2684

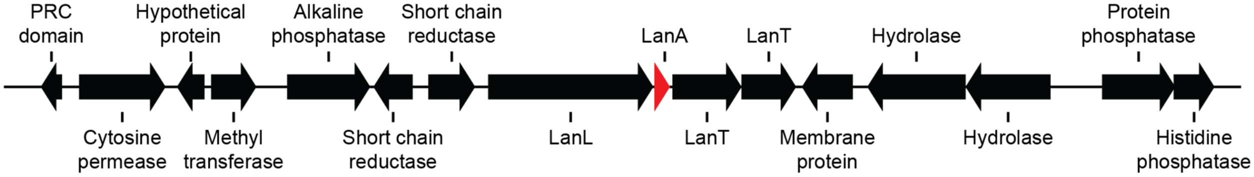

1 kb

LanA sequence MENHDIELLARLHALPETDPVGVDGAPFAATCECVGLLTLLNTVCIGISCA

Supplementary Figure S8 continued.

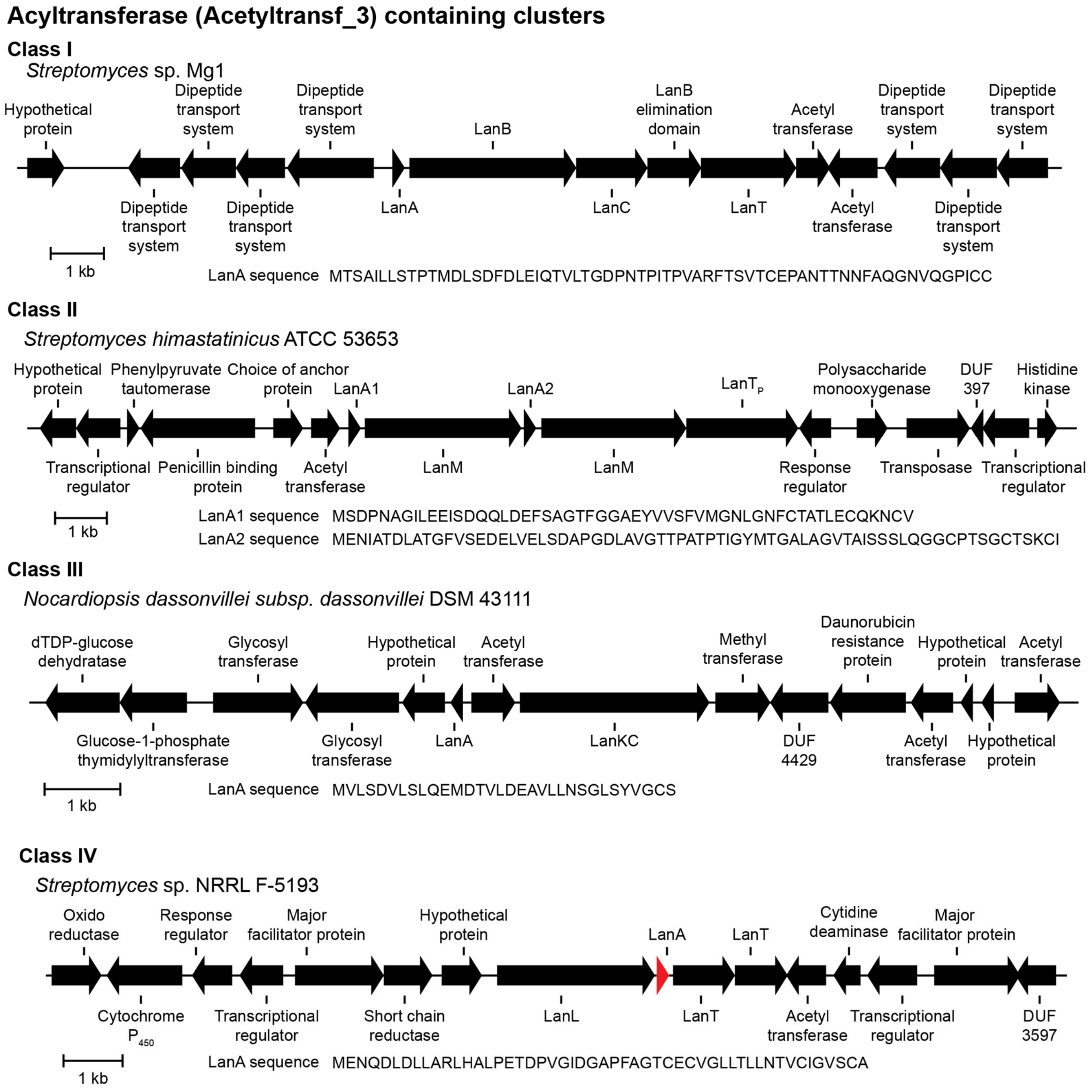

Supplementary Figure S8 continued.

Glycosyltransferase (Glycos_transf_2) containing clusters

Class I

Pedobacter heparinus DSM 2366

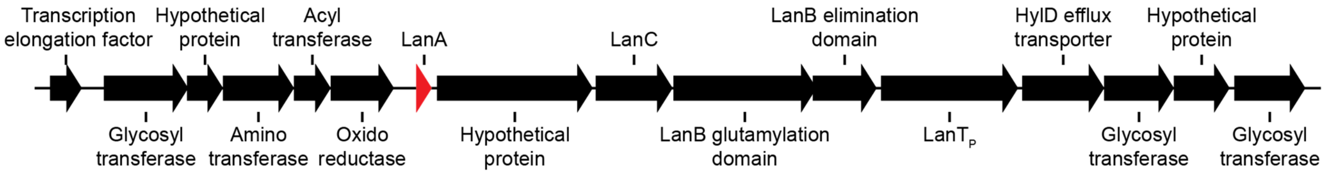

1 kb

LanA sequence MKKLRLNKSFISNLTRDEAGKIMGGNDATWEECSDRCSDYCTTPTYGDDATCYTAGAGCP GTGSYGCATDNGCQSDLCPDSLQMTECGPIC

Class II

Kitasatospora aureofaciens strain NRRL B-1286

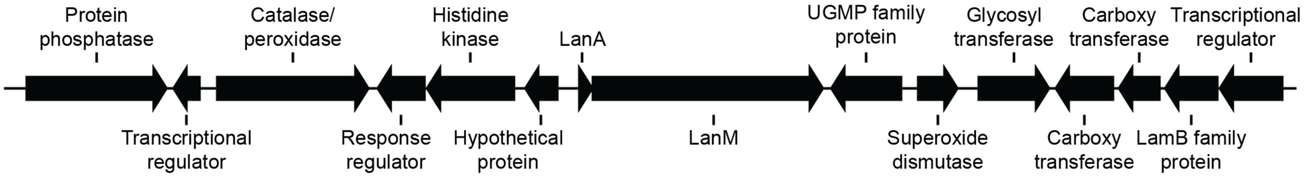

1 kb

LanA1 sequence MQPTAEQVRALKDEDFRLTLGQGHQVHPAGELADELYVTAAVLNSTCDGWSSCGRVCSY

Class III

Streptomyces pseudovenezuelae strain DSM 40212

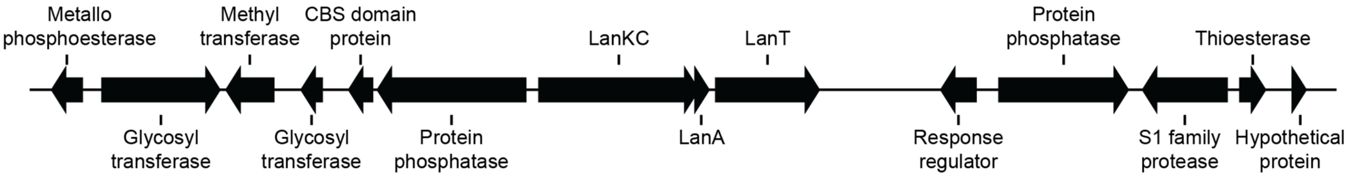

1 kb

LanA sequence MALLDLQTMESDEHTGGGGNSTLSLLSCVSAASVTLCL

Class IV

Streptomyces sp. NRRL F-5193

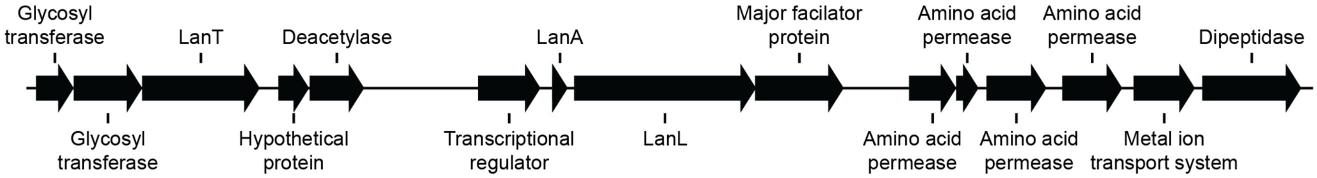

1 kb

LanA sequence MRFSKISQFPCYTVLVKHLKQILGRRSGLQKNNQRRKEMHNLMELELLPEEPVITVSREVCKWTCSFTAL

Supplementary Figure S8 continued.

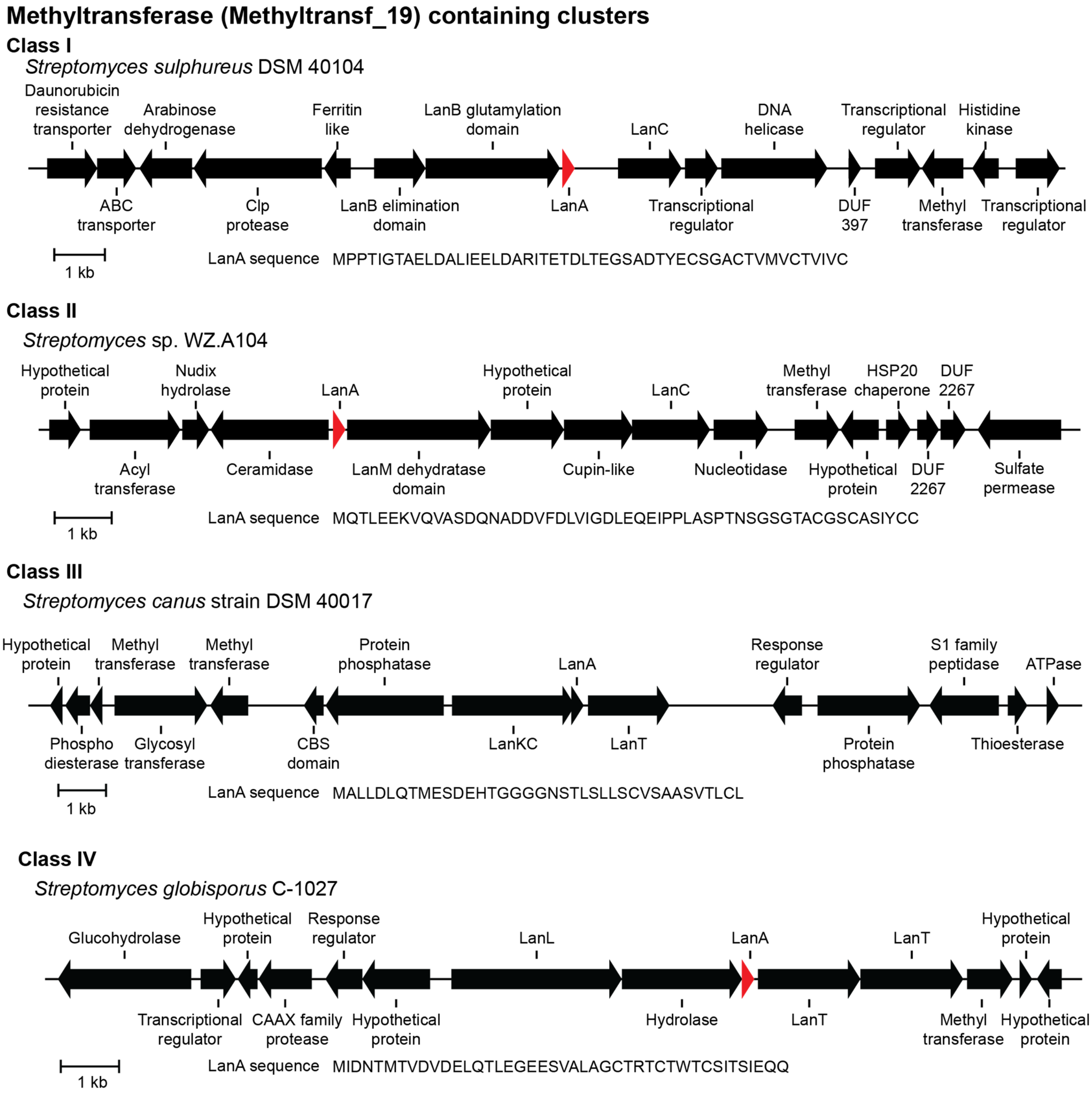

Supplementary Table S11. Distribution of select Pfam protein families from BGCs. The enzymes belonging to these families potentially carry-out secondary post-translational modifications. Values are presented on the basis of domains, so Pfam protein families that occur in multidomain proteins or single domain proteins are counted together. NRPS: non-ribosomal peptide synthetase, PKS: polyketide synthase, FAS: fatty acid synthase.

Pfam protein families	Class I	Class II	Class III	Class IV
YcaO (PF02624)	4	42	2	0
Radical_SAM (PF04055)	39	49	42	43
p450 (PF00067)	45	44	50	23
Condensation (PF00668) (NRPS)	45	31	10	2
Ketoacyl-synt_C (PF02801) (PKS/FAS)	34	5	92	8
ADH_N (PF08240) (zinc-dependent dehydrogenase)	56	63	38	27
2OG-FeII_Oxy_3 (PF13640)	0	5	4	0

Supplementary Figure S9. Example biosynthetic gene clusters encoding the enzymes in Table S11. LanA genes that were not annotated in the genome are indicated in red. Because BGC boundaries are not known, the noted enzymes may or may not be part of the lanthipeptide BGC for all panels of Supplementary Figure S9.

YcaO containing clusters

Class I

Actinokineospora enzanensis DSM 44649

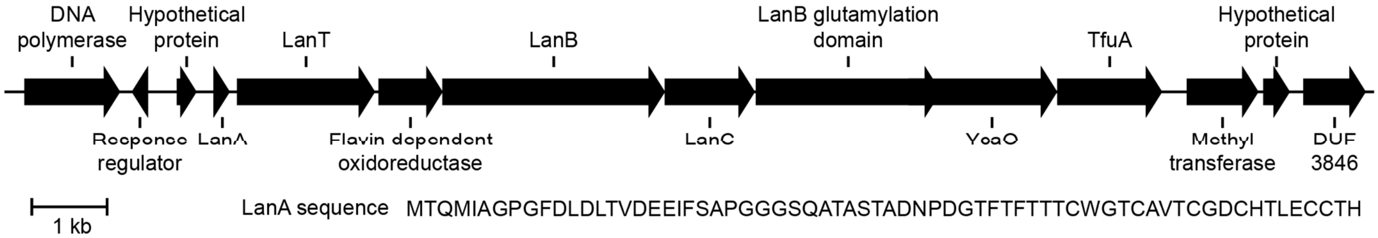

LanA sequence MTQMIAGPGFDLDLTVDEEIFSAPGGGSQATASTADNPDGTFTFTTTCWGTCAVTCGDCHTLECCTH

Class II

Chryseobacterium vrystaatense strain LMG 22846

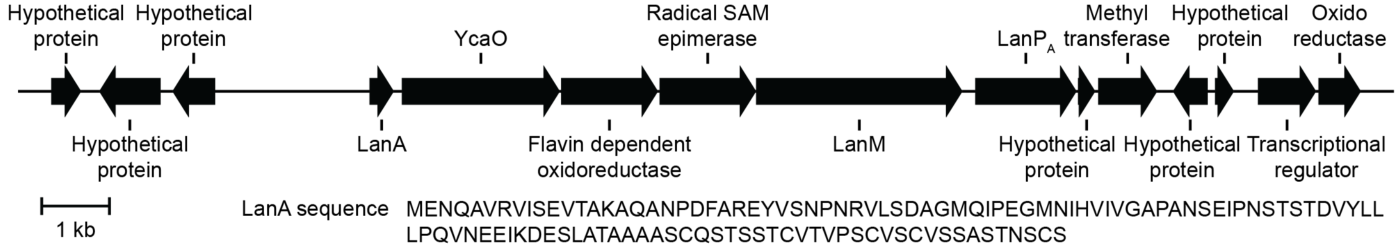

LanA sequence MENQAVRVISEVTAKAQANPDFAREYVSNPNRVLSDAGMQIPEGMNIHVIVGAPANSEIPNSTSTDVYLL
LPQVNEEIKDESLATAAAASCQSTSSTCVTVPSCVSCVSSASTNSCS

Class III

Nocardiopsis valliformis DSM 45023

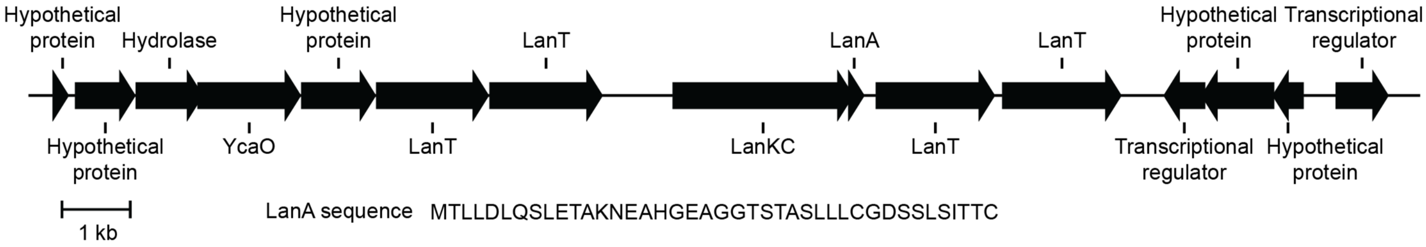

LanA sequence MTLLDLQSLETAKNEAHGEAGGTSTASLLLCGDSSLSITTC

Supplementary Figure S9 continued.

Radical SAM containing clusters

Class I

Tannerella forsythia 92A2

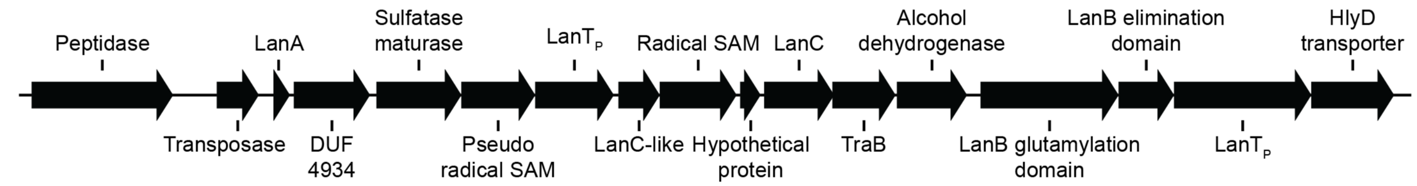

1 kb

LanA sequence MKKLKKIKLNQLCKAELNSREMAQLTGGLCCGCGCHGPSSTVDNQNANAENGYGSIGGNKICWCW DGGAWVKTETK

Class II

Butyrivibrio proteoclasticus strain P18

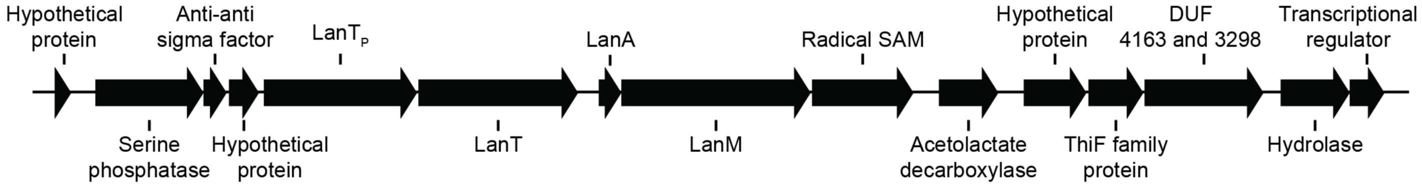

1 kb

LanA sequence MKTLQELHKEISANKDLQKAYAAAIENDAVLDFVKANGCDVTEDDIKEYISGIKKDDDTPLSVEDIENVS GGGLICKTVSVYKKMSKMAKKKGLIPDWYPC

Class III

Kibdelosporangium aridum strain A82846

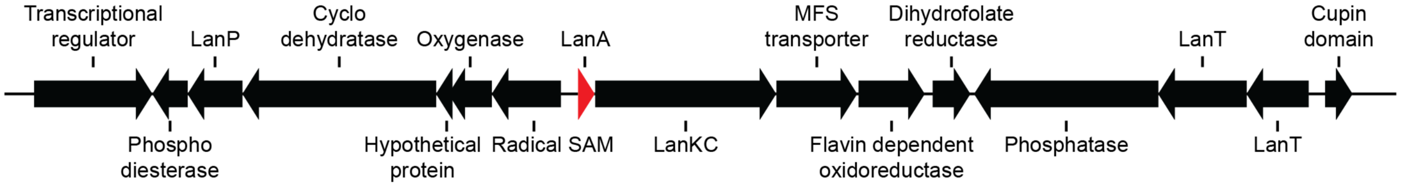

1 kb

LanA sequence MSDEKPQSSEEQSENEEVVAHAVLDLQKIQTTMPAGQIGNPAGSCSSCFAASCG

Class IV

Kibdelosporangium aridum strain A82846

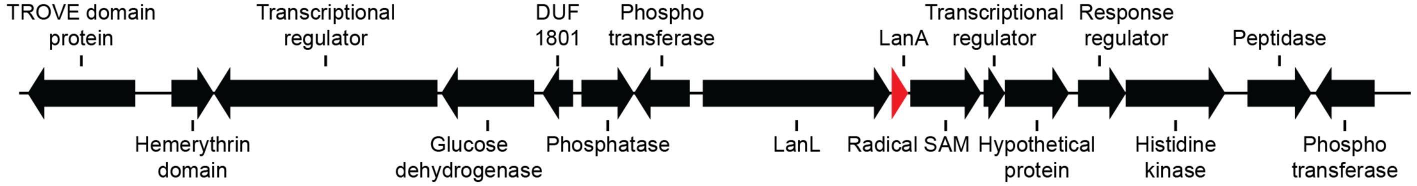

1 kb

LanA sequence MSDEKPQSSEEQSENEEVVAHAVLDLQKIQTTMPAGQIGNPAGSCSSCFAASCG

Supplementary Figure S9 continued.

Cytochrome P_{450} containing clusters

Class I

Actinomadura madurae strain DSM 43067

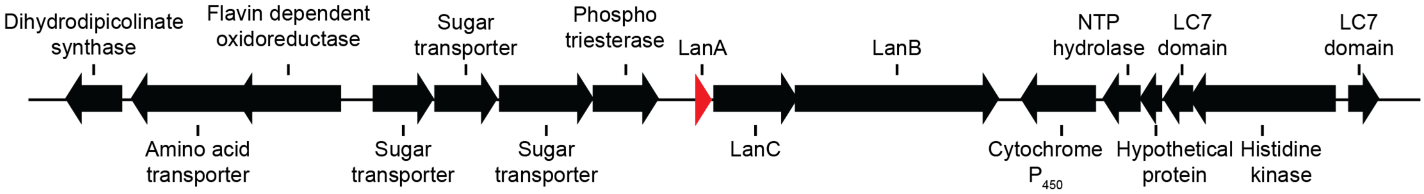

1 kb

LanA sequence MTISLTDQAGVDAQDEFDLDVRVSELSTSAEILDCTDSCPCFTAPCQSTPTCLEGI

Class II

Lentzea terrae strain NEAU-LZS 42

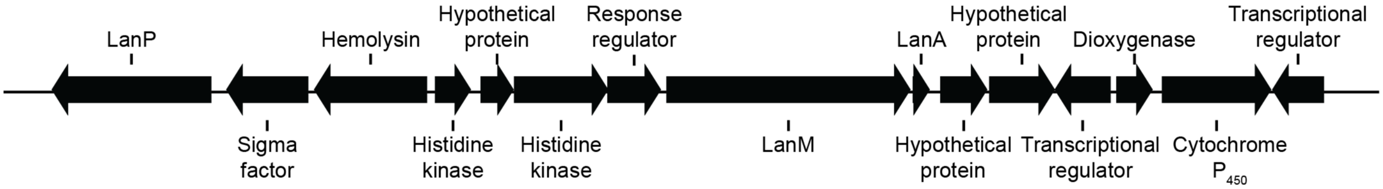

1 kb

LanA sequence MKNATIIRAWKDAAFRATLPAGAVPAHPAGSAVVKTELLGAAMGTTPICTDGGPRCTDSGPHCS

Class III

Streptomyces sp. NRRL F-4428

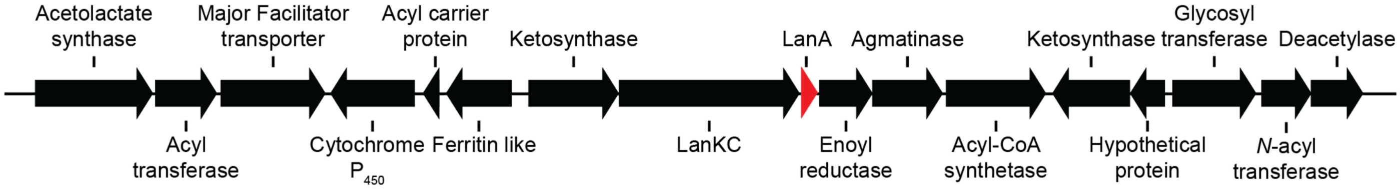

1 kb

LanA sequence MSIVLDLQGLEVPAEETTRVASTASNHC

Class IV

Streptomyces sp. NRRL F-5193

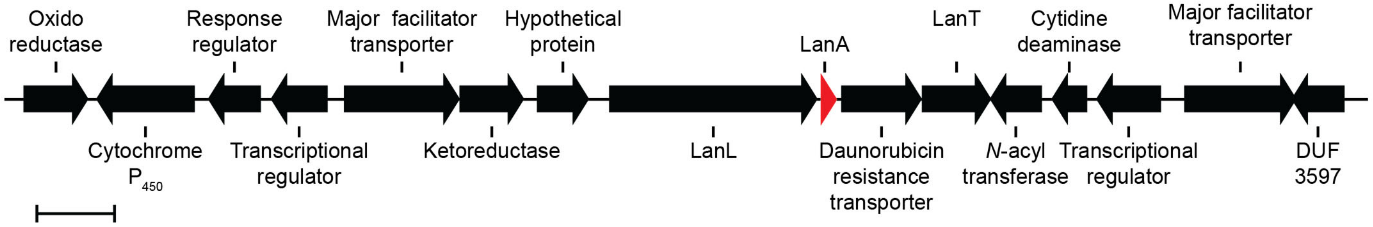

1 kb

LanA sequence MENQDLDLLARLHALPETDPVGIDGAPFAGTCECVGLLTLLNTVCIGVSCA

Supplementary Figure S9 continued.

Nonribosomal peptide synthetase containing clusters

Class I

Tumebacillus avium strain AR23208

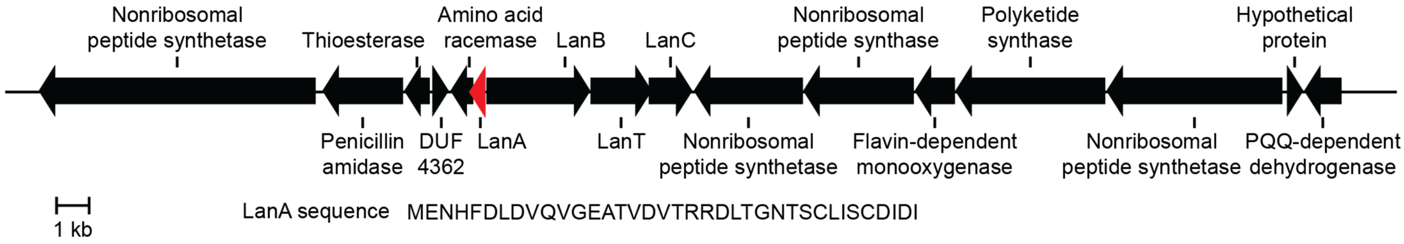

Class II

Saccharothrix variisporea strain DSM 43911

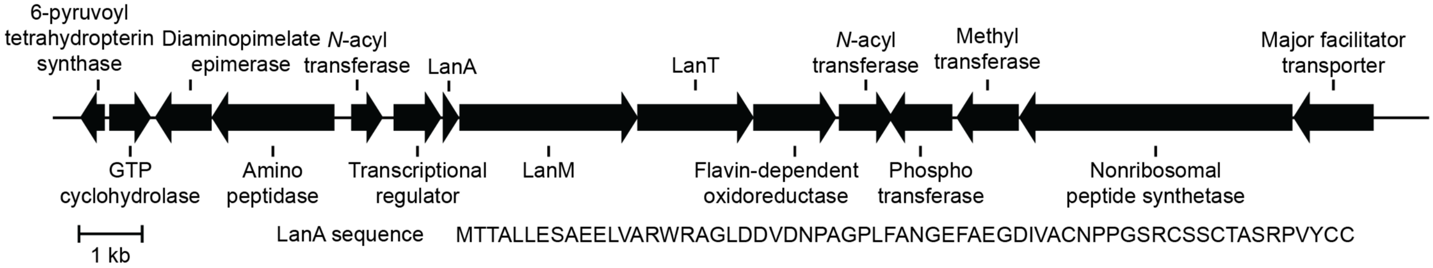

Class III

Streptomyces sp. NRRL F-4428

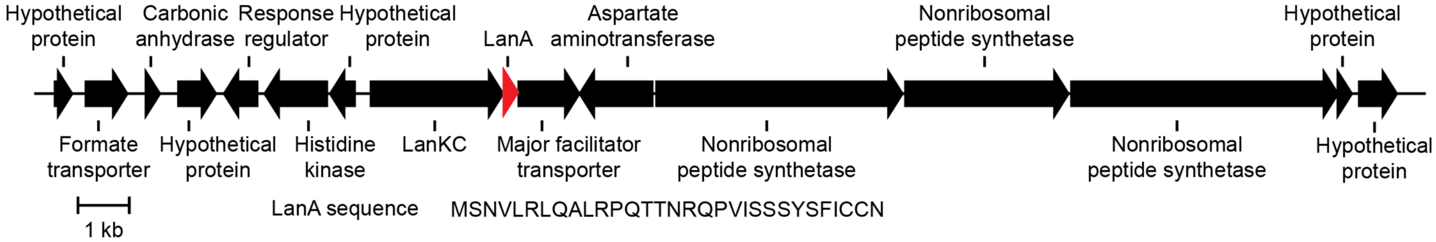

Class IV

Streptomyces venezuelae

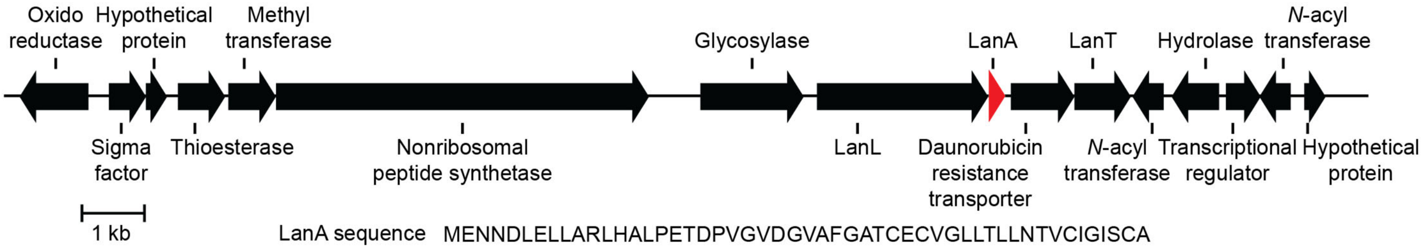

Supplementary Figure S9 continued.

Polyketide synthase containing clusters

Class I

Streptomyces formicae strain KY5

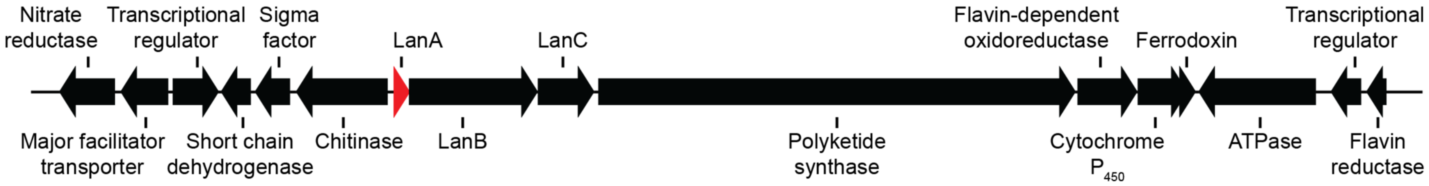

1 kb

LanA sequence MNNISAAETEFDLDLDLDTAFEDAPAMEAKSFTGTGTCLSCPVTSWHC

Class II

Actinoplanes brasiliensis strain DSM 43805

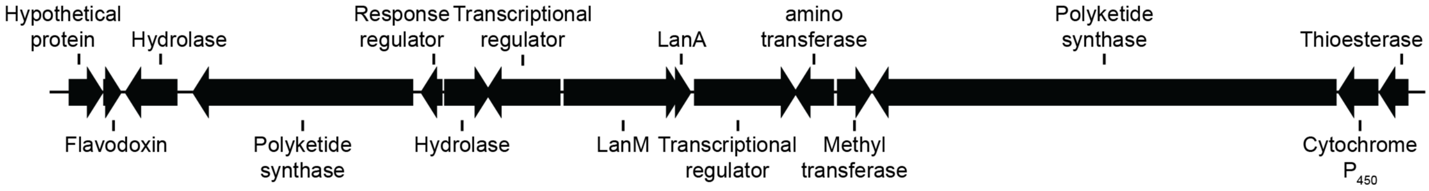

1 kb

LanA sequence MSEIKNDQNDECGESEQITEHAVGRLRLLPSVSFGTRAAALAQIALPVAVVTAGITAAVHGMGAENLVAI ETSCCPPSPR

Class III

Nocardia sp. NRRL S-836

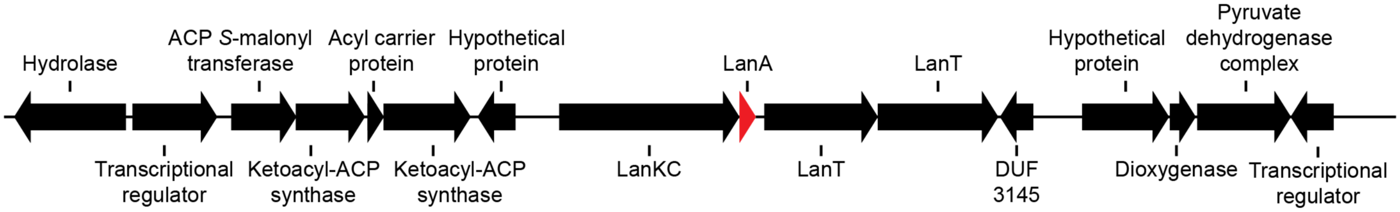

1 kb

LanA sequence MGFILDMQDLETPEAPANVLAGGSSGGGGGSTTASNASLLLPCSHSTVSLLAC

Class IV

Amycolatopsis jejuensis strain NRRL B-24427

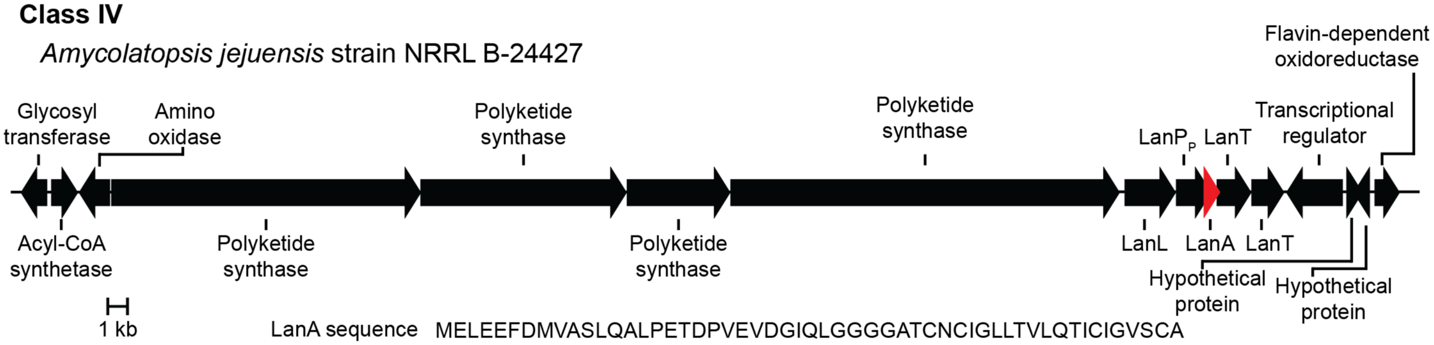

1 kb

LanA sequence MELEEFDMVASLQALPETDPVEVDGIQLGGGGATCNCIGLLTVLQTICIGVSCA

Supplementary Figure S9 continued.

Zinc-dependent alcohol dehydrogenase containing clusters

Class I

Streptomyces sp. CB02959

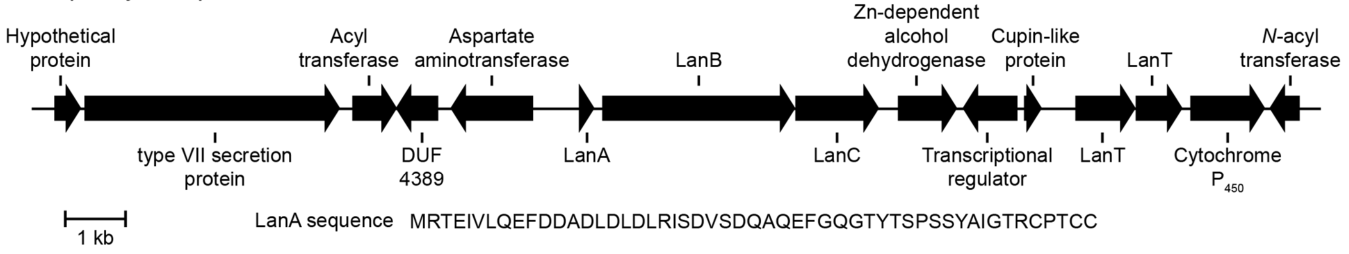

LanA sequence MRTEIVLQEFDDADLDLDLRISDVSDQAQEFGQGTYTSPSSYAIGTRCPTCC

Class II

Enterococcus faecalis strain 4928STDY7071440

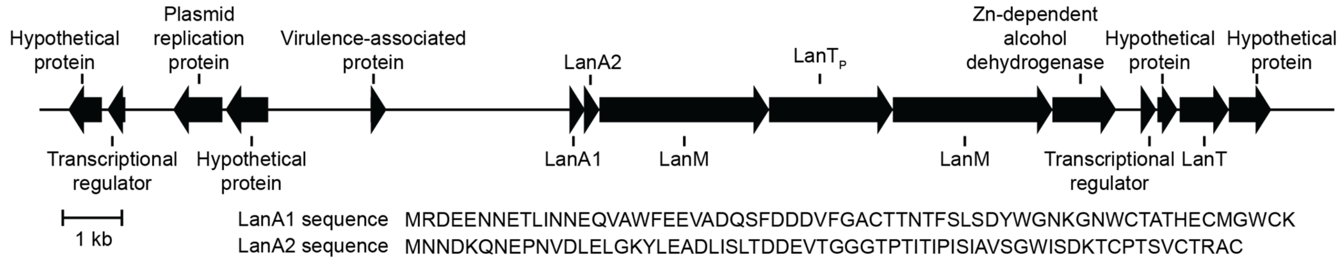

LanA1 sequence MRDEENNETLINNEQVAWFEEVADQSFDDDVFGACTTNTFSLSDYWGNKGNWCTATHECMGWCK
LanA2 sequence MNNDKQNEPNVDLELGKYLEADLISLTDDEVTGGGTPTITIPISIAVSGWISDKTCPTSVCTRAC

Class III

Nonomuraea sp. ATCC 55076

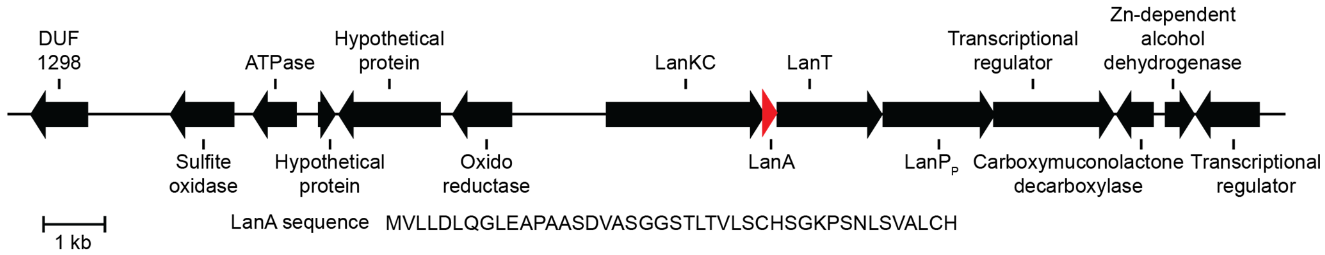

LanA sequence MVLLDLQGLEAPAASDVASGGSTLTVLSCHSGKPSNLSVALCH

Class IV

Kitasatospora sp. CB01950

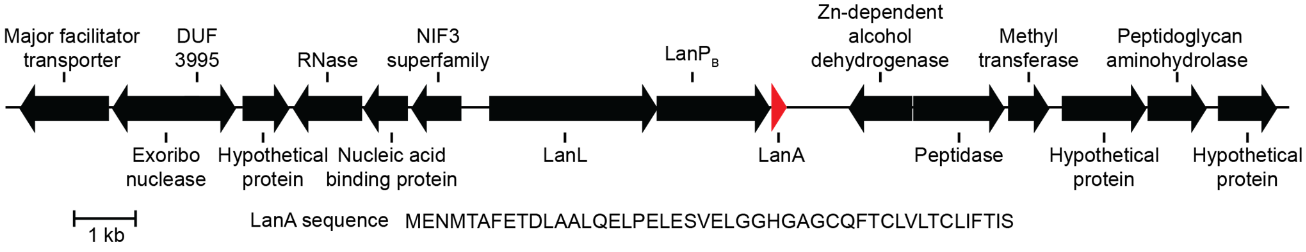

LanA sequence MENMTAFETDLAALQELPELESVELGGHGAGCQFTCLVLTCLIFTIS

Supplementary Figure S10. Phylogenetic distribution of genomes in the dataset used for this study.

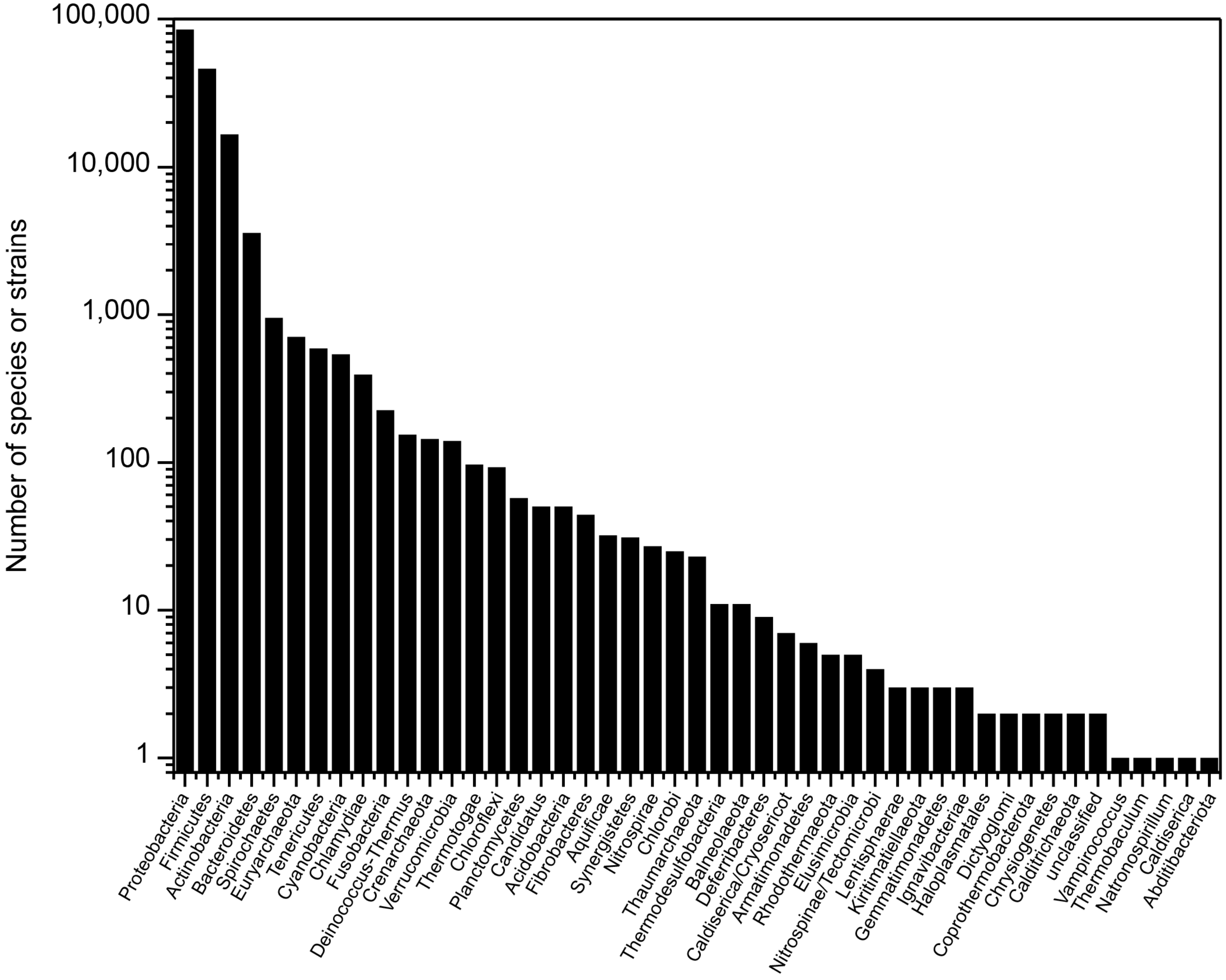

Supplementary Figure S11. An approximate maximum likelihood, midpoint rooted phylogenetic tree of LanC and LanC-like domains including human LanC-like proteins. The branches for the human LanC-like proteins are colored in red.

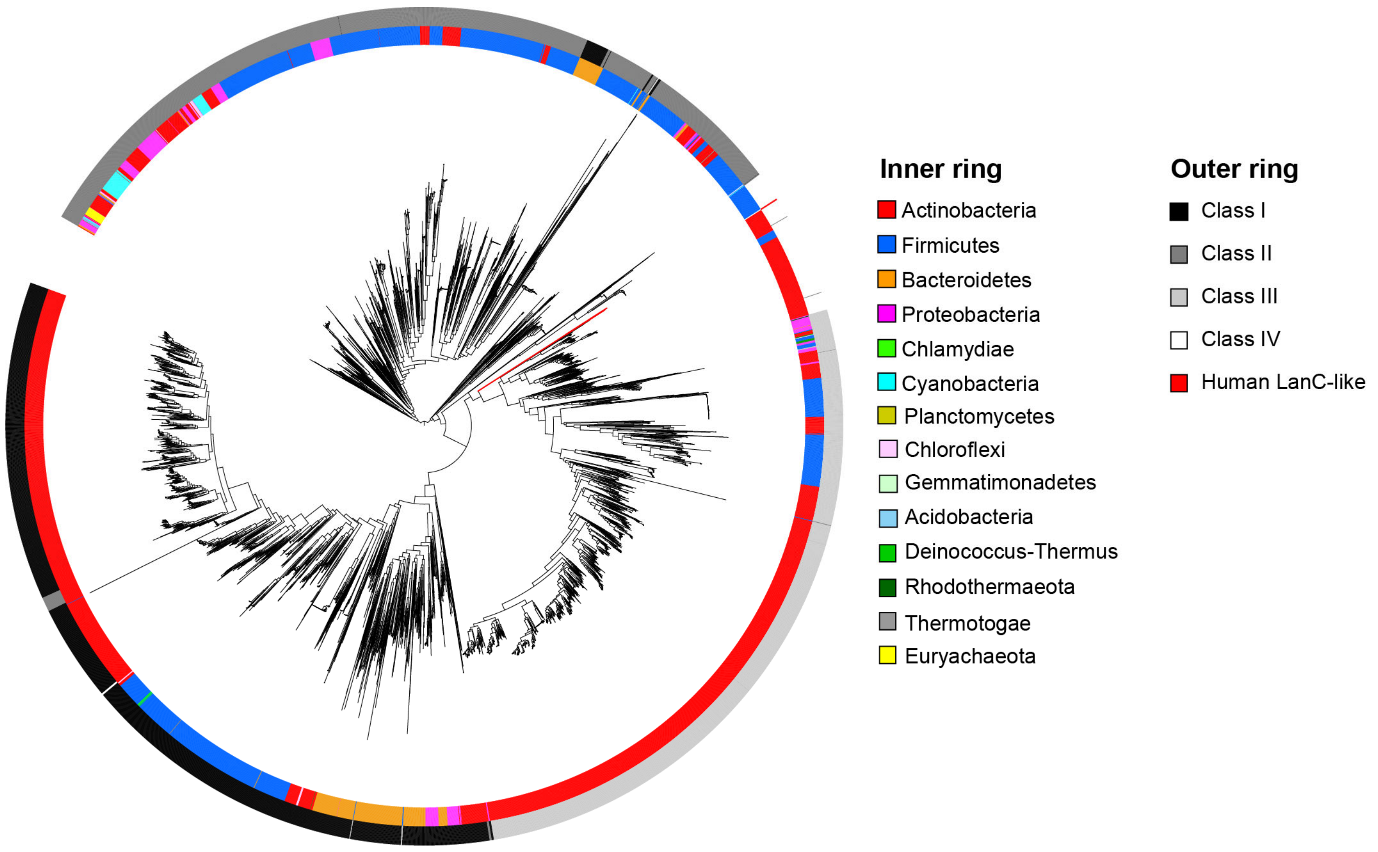

Supplementary Figure S12. GC content of clusters versus the cognate genome. The blue diagonal line is the linear regression with slope and intercept provided. Red points are the 10 greatest regression outliers. The box plot represents the differences between the cluster GC content and the genome GC content. The boxes indicate 1 standard deviation of the mean with the whiskers representing the maximum and minimum values.

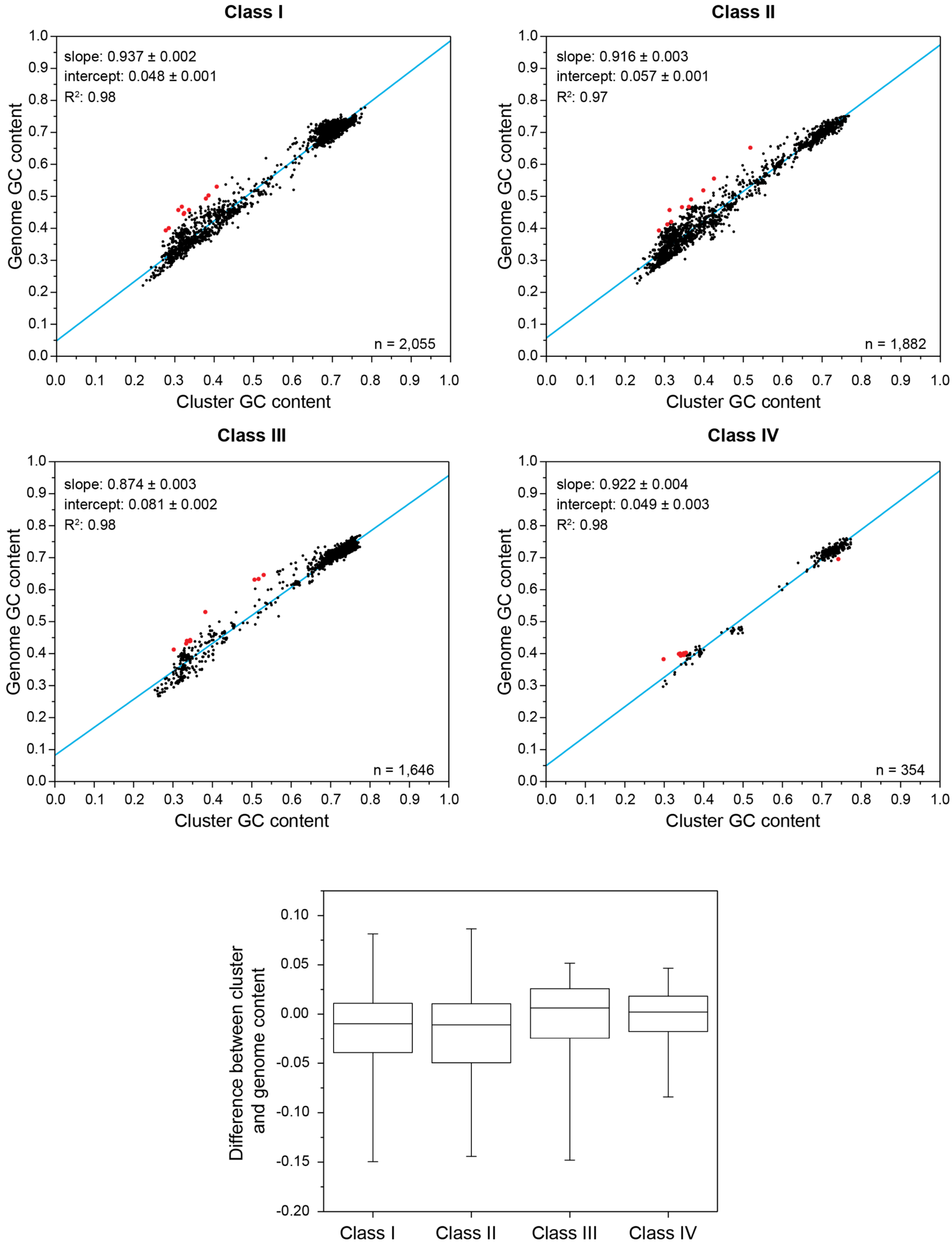

Supplementary Figure S13. ESI MS/MS and bioactivity of birimositide α and β. A+B) Collision induced dissociation fragmentation spectrum of each peptide. The multiply-charged monoisotopic mass is boxed in blue. C) Peptides were screened against *Lactococcus lactis* sp. *cremoris* (left) and *Micrococcus luteus* ATCC 4698 (right). Red arrows indicate zones of growth inhibition.

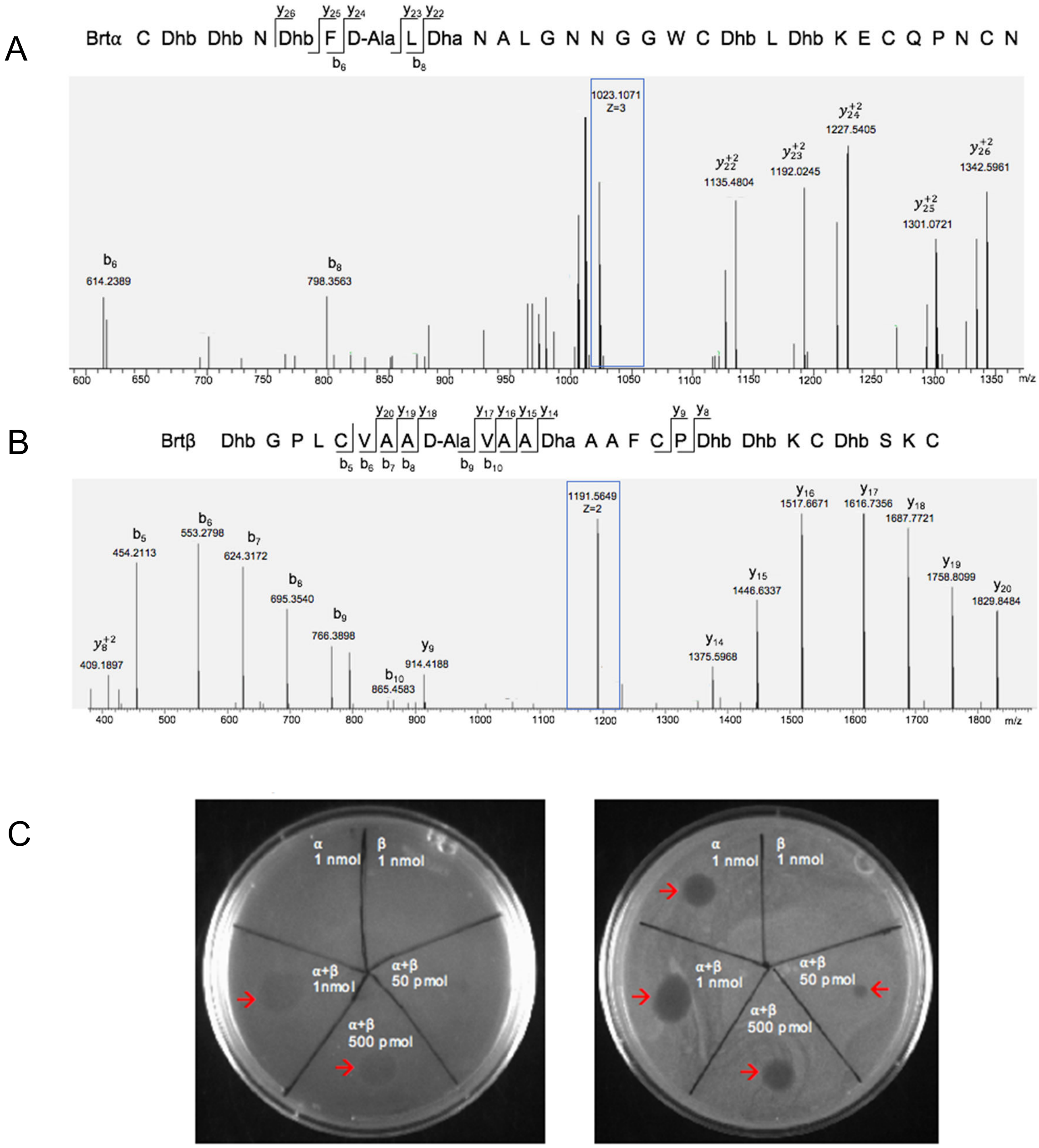

Supplementary Table S12. Expected and observed monoisotopic masses for Brtα and Brtβ using orbitrap ESI MS/MS.

Ion	Predicted (m/z)	Observed (m/z)	Error (ppm)
$(Brt\alpha + 3H)^{3+}$	1023.1071	1023.1071	0.0000
$(Brt\alpha + 3H)^{3+}$ y_{26}^{+2}	1342.5938	1342.5961	-1.7131
$(Brt\alpha + 3H)^{3+}$ y_{25}^{+2}	1301.0752	1301.0721	2.3826
$(Brt\alpha + 3H)^{3+}$ y_{24}^{+2}	1227.5410	1227.5405	0.4073
$(Brt\alpha + 3H)^{3+}$ y_{23}^{+2}	1192.0225	1192.0245	-1.6778
$(Brt\alpha + 3H)^{3+}$ y_{22}^{+2}	1135.4804	1135.4804	0.0000
$(Brt\alpha + 3H)^{3+}$ b_8	798.3603	798.3563	5.0103
$(Brt\alpha + 3H)^{3+}$ b_6	614.2391	614.2389	0.3256

$(Brt\beta + 2H)^{2+}$	1191.5649	1191.5649	0.0000
$(Brt\beta + 2H)^{2+}$ y_{20}	1829.8495	1829.8484	0.6011
$(Brt\beta + 2H)^{2+}$ y_{19}	1758.8124	1758.8099	1.4214
$(Brt\beta + 2H)^{2+}$ y_{18}	1687.7753	1687.7721	1.8960
$(Brt\beta + 2H)^{2+}$ y_{17}	1616.7382	1616.7356	1.6082
$(Brt\beta + 2H)^{2+}$ y_{16}	1517.6698	1517.6671	1.7790
$(Brt\beta + 2H)^{2+}$ y_{15}	1446.6327	1446.6337	-0.6913
$(Brt\beta + 2H)^{2+}$ y_{14}	1375.5956	1375.5968	-0.8723
$(Brt\beta + 2H)^{2+}$ y_9	914.4223	914.4188	3.8276
$(Brt\beta + 2H)^{2+}$ b_{10}	865.4600	865.4583	1.9643
$(Brt\beta + 2H)^{2+}$ b_9	766.3916	766.3898	2.3487
$(Brt\beta + 2H)^{2+}$ b_8	695.3545	695.3540	0.7191
$(Brt\beta + 2H)^{2+}$ b_7	624.3174	624.3172	0.3203
$(Brt\beta + 2H)^{2+}$ b_6	553.2803	553.2798	0.9037
$(Brt\beta + 2H)^{2+}$ b_5	454.2119	454.2113	1.3210
$(Brt\beta + 2H)^{2+}$ y_8^{+2}	409.1884	409.1897	-3.1770

References

1. Grant CE, Bailey TL, Noble WS: **FIMO: Scanning for occurrences of a given motif**. *Bioinformatics* 2011, **27**(7):1017-1018.
2. van der Meer JR, Rollema HS, Siezen RJ, Beerthuyzen MM, Kuipers OP, de Vos WM: **Influence of amino acid substitutions in the nisin leader peptide on biosynthesis and secretion of nisin by *Lactococcus lactis***. *J Biol Chem* 1994, **269**(5):3555-3562.
3. Patton GC, Paul M, Cooper LE, Chatterjee C, van der Donk WA: **The importance of the leader sequence for directing lanthionine formation in lacticin 481**. *Biochemistry* 2008, **47**(28):7342-7351.
4. Müller WM, Ensle P, Krawczyk B, Süssmuth RD: **Leader peptide-directed processing of labyrinthopeptin A2 precursor peptide by the modifying enzyme LabKC**. *Biochemistry* 2011, **50**(39):8362-8373.
5. Hegemann JD, van der Donk WA: **Investigation of substrate recognition and biosynthesis in class IV lanthipeptide systems**. *J Am Chem Soc* 2018, **140**(17):5743-5754.
6. Zallot R, Oberg NO, Gerlt JA: **'Democratized' genomic enzymology web tools for functional assignment**. *Curr Opin Chem Biol* 2018, **47**:77-85.
7. Shannon P, Markiel A, Ozier O, Baliga NS, Wang JT, Ramage D, Amin N, Schwikowski B, Ideker T: **Cytoscape: a software environment for integrated models of biomolecular interaction networks**. *Genome Res* 2003, **13**(11):2498-2504.
8. Medema MH, Kottmann R, Yilmaz P, Cummings M, Biggins JB, Blin K, de Bruijn I, Chooi YH, Claesen J, Coates RC *et al*: **Minimum Information about a Biosynthetic Gene cluster**. *Nat Chem Biol* 2015, **11**(9):625-631.
9. van Heel AJ, de Jong A, Song C, Viel JH, Kok J, Kuipers OP: **BAGEL4: a user-friendly web server to thoroughly mine RiPPs and bacteriocins**. *Nucleic Acids Res* 2018, **46**(W1):W278-w281.
10. Crooks GE, Hon G, Chandonia JM, Brenner SE: **WebLogo: a sequence logo generator**. *Genome Res* 2004, **14**(6):1188-1190.
